# Covalent cell surface recruitment of chemotherapeutic polymers enhances selectivity and activity†

**DOI:** 10.1039/d0sc06580c

**Published:** 2021-02-12

**Authors:** Ruben M. F. Tomás, Matthew I. Gibson

**Affiliations:** Department of Chemistry, University of Warwick Coventry CV4 7AL UK m.i.gibson@warwick.ac.uk; Warwick Medical School, University of Warwick Coventry CV4 7AL UK; MAS CDT, University of Warwick Coventry CV4 7AL UK

## Abstract

Synthetic macromolecular chemotherapeutics inspired by host defence peptides can disrupt cell membranes and are emerging as agents for the treatment of cancer and infections. However, their off-target effects remain a major unmet challenge. Here we introduce a covalent recruitment strategy, whereby metabolic oligosaccharide engineering is used to label targeted cells with azido glycans, to subsequently capture chemotherapeutic polymers by a bio-orthogonal click reaction. This results in up to 10-fold reduction in EC_50_ and widening of the therapeutic window. Cell death is induced by not only membrane leakage, but also by apoptosis due to the conjugated chemotherapeutic being internalised by glycan recycling. Covalent recruitment also lead to increased penetration and significant cell death in a 3-D tumour model in just 3 hours, whereas doxorubicin required 24 hours. This conceptual approach of ‘engineering cells to capture polymers’ rather than ‘engineering polymers to target cells’ will bring new opportunities in non-traditional macromolecular therapeutics.

## Abstract 2

Chemotherapeutic polymers are targeted to cells by introduction of unnatural glycans to their glycocalyx, enhancing their cytotoxic effect.

## Graphical abstract

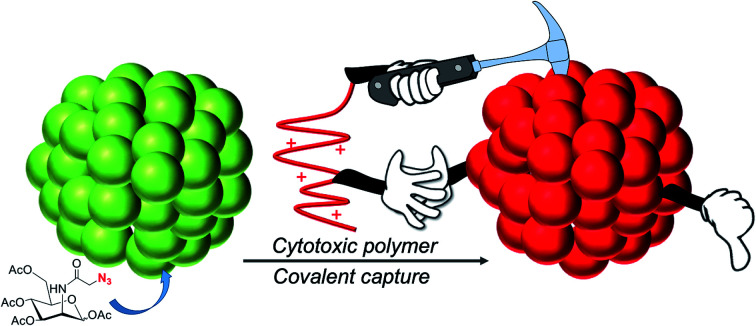

## Introduction

Chemotherapeutic cancer treatments, although widely used, have many outstanding issues limiting their therapeutic performance. Cancer cells often possess intrinsic drug resistance and can develop multidrug resistance (MDR) which is largely believed to be a stable and heritable process.^1^ This has led to the development of several distinct classes of chemotherapeutics including alkylating agents,^2^ anti-metabolites,^3^ topoisomerase inhibitors^4^ and mitotic inhibitors.^5,6^ In addition to aggressive resistance development, low aqueous solubility, insufficient drug accumulation, off-target toxicity and rapid clearance remain major drawbacks.^7^ Advancements in drug delivery carrier systems may in the future mitigate some of these issues but the development of drug resistance mechanisms and cellular barriers still remain a concern.^8^ Drug delivery systems are also limited by burst release, off-target toxicity, limited efficacy of the enhanced permeation and retention (EPR) effect and endogenous target-receptor saturation.^9,10^

Synthetic macromolecular therapeutic agents that mimic host defence peptides (HDPs) and antimicrobial peptides (AMPs) have been widely explored as potent antimicrobial agents.^11–13^ These amphiphilic, cationic therapeutic agents disrupt bacterial cell surfaces through electrostatic interactions with anionic membrane components and have improved pharmacokinetic properties compared to their natural counterparts.^14^ Synthetic and natural HDPs also possess tumouricidal properties by selectively disrupting cancer cell membranes due to the presence of excess anionic constituents including phosphatidylserine, sialic acid, and heparin sulphate.^15–18^ However, for both antimicrobial and anticancer applications, selectivity for diseased cells, verses healthy, is a key obstacle;^19^ the polymers must kill the cancer cells at concentrations far below that of killing healthy cells. Current strategies to improve cationic polymer therapeutic function have focused on altering structural complexity (*e.g.* copolymers with hydrophobic/philic groups) in search of a ‘sweet spot’ where anti-tumoural activity exceeds intrinsic cell toxicity.^16,20–22^ Micelles generated from biodegradable triblock copolymers of PEG, guanidinium-functionalised polycarbonate (PGC) and polylactide (PLL), and also cationic diblock polycarbonates, possess activity against multiple cancerous cell lines.^20,22^ Treatment of 4T1 mouse breast tumour-bearing mice with cationic PGC polymers (20 mg kg^−1^) lead to a ∼3-fold reduction in tumour volume *in vivo*.^22^ However, it is crucial to note that these HDP/AMP mimics only induce necrosis unlike their natural counterparts which trigger additional (non-lytic) apoptotic mechanisms to ensure that resistance development is unlikely and provide broad-spectrum activity.^17^ In addition, the reliance of non-specific electrostatic and hydrophobic interactions as targeting mechanisms limits selectivity, so there is a clear need to explore alternative methods to deliver polymer-based chemotherapeutics to cells.

The above cancer targeting methods focus on the ‘magic bullet’ (Ehrlich) approach, where the carrier is tuned to locate the disease through targeting groups, physicochemical tuning or by following metabolic gradients.^23,24^ As selectivity remains the major issue, we therefore considered the opposite, that cancerous cells themselves can be tuned for bio-orthogonal covalent recruitment of synthetic chemotherapeutic macromolecules by introducing cell surface unnatural target receptors.^25^ Metabolic oligosaccharide engineering (MOE) enables the selective introduction of bio-orthogonal reactive ‘handles’ onto cell surfaces *in vitro* and *in vivo* by hijacking the promiscuous glycan biosynthetic pathways of endogenous sugars, which we have previously used to ‘capture’ abiotic polymeric materials with the correct reactive handle (*e.g.* azide/alkyne).^26–28^ Nanoparticle delivery to tumour-bearing mice has been accomplished *in vivo* utilising MOE for the delivery of imaging agents,^29^ chemotherapeutic drugs^30^ and photodynamic therapy.^31^ In addition, Park *et al.* devised a glycan precursor which is activated by intra-tumoural hydrogen peroxide for the recruitment of antibodies and a photosensitizer (Zn-tetraphenylporphyrin) to induce natural killer cell and reactive oxygen species-mediated cell death, respectively.^32^ Therefore, we hypothesised that tumour cells modified with unnatural glycans would enable the covalent capture of suitably modified cationic AMP/HDP mimetic polymers to enhance the concentration localised at the cancer cell surface whilst minimising treatment dosage to reduce off-target toxicity and increase selectivity. Furthermore, by targeting glycans, the cationic polymers can possibly enter the cell during glycan recycling to access the mitochondria, golgi apparatus and nucleus which are known mechanisms to induce apoptosis, in addition to necrosis from membrane damage, an advantage to current AMP/HDP mimics.^33,34^

Herein, we report an alternative to the current approach of iteratively ‘engineering chemotherapeutic polymers to target cells’ and instead show that ‘engineering cells to capture polymers’ leads to gains in efficacy and selectivity. This is achieved using metabolic oligosaccharide engineering of tumour cells to introduce azides into their glycocalyx, which selectively captures chemotherapeutic polymers by copper-free click reactions. Comparable or superior EC values were achieved compared to structurally complex AMP mimics and small molecule chemotherapeutics. The covalent capture of chemotherapeutic polymers was also shown to induce additional apoptotic pathways compared to non-covalent strategies and enhanced activity was demonstrated in a 3-D tumour model.

## Results and discussion

### Synthesis of macromolecular chemotherapeutic

**Fig. 1:**
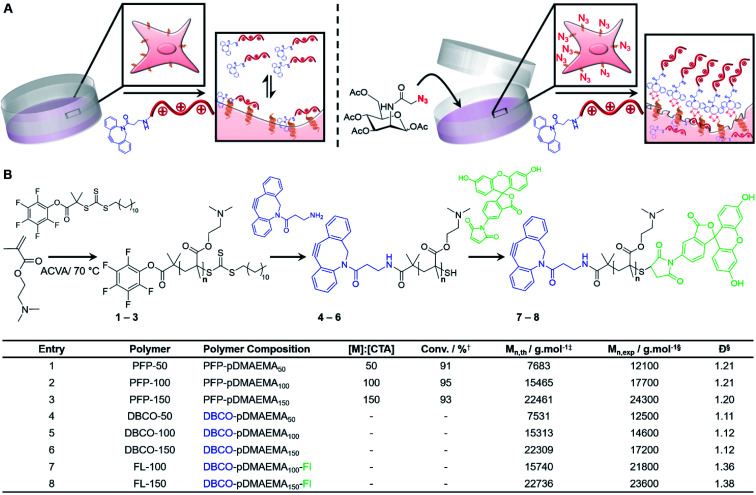
Polymer synthesis and concept. (A) A two-step approach to develop a selective macromolecular chemotherapeutic system was devised relying on initial installation of azido glycans onto the cell surface using MOE followed by treatment with a DBCO-modified polycation; (B) synthesis of DBCO-modified pDMAEMA_*n*_ with SEC characterisation in the table. ^†^ Monomer conversion was determined by ^1^H NMR spectroscopy. ^‡^ Theoretical molecular weight (*M*_n,th_) was calculated based on monomer conversion. ^§^ Molecular weight (*M*_n,exp_) and dispersity (*Đ*) was determined by SEC analysis.

Poly[2-(dimethylamino)ethyl methacrylate] (pDMAEMA) was selected as a model polycation, due to its well-established cytotoxicity/membrane lytic profile,^35–37^ to probe if covalent recruitment onto azido labelled cells can enhance its low-to-moderate cytotoxic properties, Fig. 1A. Well-defined, dibenzocyclooctyne (DBCO) terminated cationic pDMAEMA polymers (DBCO-pDMAEMA_*n*_, **4–6**) were synthesised *via* reversible addition–fragmentation chain-transfer (RAFT) polymerisation of DMAEMA with a pentafluorophenyl 2-(dodecylthiocarbonothioylthio)-2-methylpropionic acid (PFP-DMP) RAFT agent (PFP-pDMAEMA_*n*_, **1–3**), followed by displacement of the PFP with DBCO-NH_2_, Fig. 1B. Polymer characterisation and DBCO conjugation was achieved using ^1^H, ^13^C and ^19^F NMR, IR and SEC (Fig. S4–S15 and Table S1–S3†), showing low dispersity values (1.1–1.2), Fig. 1B. DBCO-pDMAEMA_*n*_ was conjugated with *N*-(5-fluoresceinyl)maleimide (Fl) *via* the *ω*-terminal thiol to provide imaging capabilities (DBCO-DMAEMA_*n*_–Fl, **7–8**), however DBCO-pDMAEMA_*n*_ polymers unlabelled with fluorescein (**4–6**) were used for viability and cytotoxicity assays to prevent colorimetric interference.

### Enhancing chemotherapeutic macromolecules using a two-step MOE approach

**Fig. 2:**
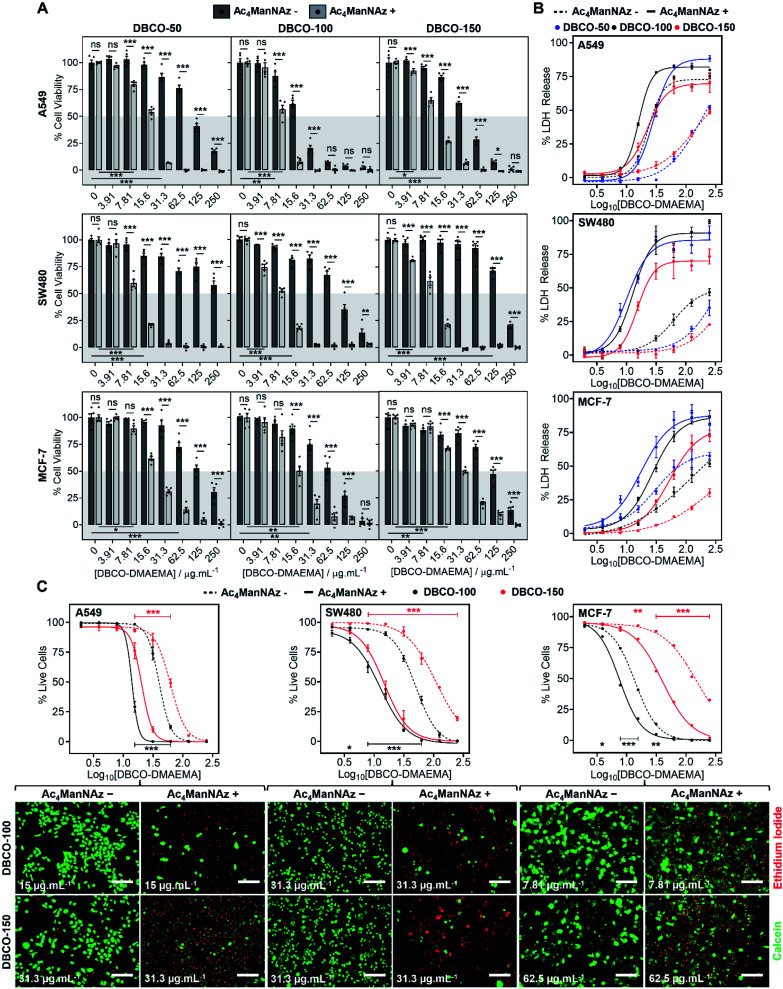
Enhancing polymer cytotoxicity with MOE. (A) Cell viability was monitored by resazurin reduction (*n* = 5), (B) membrane leakage assessed *via* LDH release assay (*n* = 3) and (C) percentage live cells determined using calcein (green) and ethidium iodide (red) staining (*n* = 4) of cells following treatment with (+) and without (−) Ac_4_ManNAz (40 μM, 96 h) and DBCO-pDMAEMA_*n*_. Representative live/dead images are provided at DBCO-pDMAEMA_*n*_ concentrations where Ac_4_ManNAz significantly increased cell death. Data is represented by mean ± SEM of *n* biological repeats (ANOVA, Tukey PostHoc; ns: *p* ≥ 0.05, *p ≤ 0.05, **p ≤ 0.01, ***p ≤ 0.001). Scale bar = 200 μm.

The core hypothesis of this work is that MOE can be used to enhance the chemotherapeutic properties of cationic polymers, by using cell surface installed azido glycans to capture a complementary strained alkyne cationic polymer, localise it at the cell membrane and hence selectively enhance activity. To explore this, three cancerous cell lines (A549, SW480 and MCF-7) were treated with *N*-azidoacetylmannosamine-tetraacylated (Ac_4_ManNAz) (40 μM, 96 h), to introduce azides to the glycocalyx, and subsequently incubated with DBCO-pDMAEMA_50–150_ (0–250 μg mL^−1^) for 2.5 h. Cell viability was measured 24 h post-treatment and compared to cells untreated with Ac_4_ManNAz but still treated with DBCO-pDMAEMA_50–150_ (0–250 μg mL^−1^, 2.5 h) using the resazurin reduction assay (Fig. 2A). In line with our core hypothesis, the chemotherapeutic activity of DBCO-pDMAEMA_*n*_ was significantly increased against azido glycan labelled cells compared to unlabelled cells at equal DBCO-pDMAEMA_*n*_ and a reduction in the concentration required to induce significant cell death was observed; confirming successful enhancement of DBCO-pDMAEMA_*n*_'s chemotherapeutic properties. Dose–response curves (in full in Fig. S17†) were plotted from the resazurin data to calculate EC_50_ values (effective concentration at which cell viability was reduced by 50%) for DBCO-pDMAEMA_50–150_ against cells treated with and without Ac_4_ManNAz and have been summarised in Table 1. For each cell and polymer combination, a drop in EC_50_ values was observed with the incorporation of azido glycans. SW480 cells showed the largest increase in activity (*i.e.* reduced EC_50_) of 92.6%, whereas A549 and MCF-7 cells showed slightly lower decreases in EC_50_ of 71.8% and 78.6%, respectively. The variation between cell type and changes in EC_50_ values can be attributed to differences in glycan composition, growth rates and overall stability of cell type to chemical stress. DBCO-100 possessed the lowest EC_50_ values across all cell types, despite previous reports suggesting that pDMAEMA molecular weight weakly influences cytotoxicity,^35,38^ whereas DBCO-50 and DBCO-150 polymers offered higher selectivity towards azido labelled. Indeed, through careful selection of polymer chain length and concentrations it was possible to identify conditions where unlabelled cells retain >90% cell viability whereas metabolically labelled cells drop to <20% with DBCO-50 and DBCO-150 polymers, Fig. 2A. Compared to the highly potent PEG–PGC_*m*_–PLA_*n*_ triblock polymers tested by Zhong *et al.* on A549 cell lines,^22^ DBCO-pDMAEMA_50–150_ achieved lower EC_50_ values (average 2.5-fold lower) with the incorporation of azido glycans on the same cell line demonstrating that covalent recruitment of chemotherapeutic macromolecules significant enhances activity beyond complex macromolecular engineering.

**Table 1:** Summary EC_50_ values of DBCO-DMAEMA_*n*_ (0–250 μg mL^−1^, 2.5 h) with (+) and without (−) Ac_4_ManNAz pre-treatment determined by resazurin and live/dead assays

Polymer	EC_50_ (μg mL^−1^)					
	A549		SW480		MCF-7	
	−N_3_	+N_3_	−N_3_	+N_3_	−N_3_	+N_3_
DBCO-50	102.0 ± 2.7^a^	15.8 ± 0.6^a^	>250^a^	9.2 ± 0.3^a^	144.6 ± 13.7^a^	21.1 ± 0.7^a^
DBCO-100	19.3 ± 0.9^a^	8.4 ± 0.3^a^	100.7 ± 10.1^a^	8.9 ± 0.4^a^	65.8 ± 6.2^a^	16.6 ± 1.6^a^
	39.9 ± 2.4^b^	13.9 ± 0.4^b^	45.2 ± 2.2^b^	10.9 ± 1.3^b^	12.9 ± 0.4^b^	7.5 ± 0.3^b^
DBCO-150	38.7 ± 1.6^a^	9.8 ± 0.3^a^	166.9 ± 2.0^a^	10.0 ± 0.5^a^	128.0 ± 10.1^a^	31.1 ± 1.0^a^
	58.8 ± 1.0^b^	19.7 ± 1.1^b^	108.8 ± 4.1^b^	14.7 ± 2.2^b^	145.8 ± 20.0^b^	37.1 ± 2.5^b^

a Average EC_50_ value from resazurin dose–response curves ± SEM of 5 biological repeats.

b Average EC_50_ value from calcein and EI dose–response curves ± SEM of 4 biological repeats.

To compare DBCO-pDMAEMA_*n*_ against a conventional chemotherapeutic, azido glycan labelled and unlabelled cells were treated with doxorubicin (0–250 μg mL^−1^) for 2.5 h, 24 h and 48 h and cell viability was measured using the resazurin assay, Fig. S18.† DBCO-pDMAEMA_*n*_ was able to act rapidly, in 2.5 h, whereas doxorubicin required over 24 h incubation to induce significant cell death (with no effect of −/+ Ac_4_ManNAz) showing that covalent recruitment of polycations has a kinetic benefit compared to some small molecule chemotherapeutics. DBCO-pDMAEMA_*n*_ was able to reduce the cell viability of Ac_4_ManNAz labelled SW480 and MCF-7 cells more than doxorubicin even post 24 h incubation. DBCO-pDMAEMA_*n*_ (0–500 μg mL^−1^, 3 h) caused insignificant levels of haemolysis against ovine blood (<3%), Fig. S19,† demonstrating the necessary biocompatibility for *in vivo* translation.

Chemotherapeutic polycationic polymers have been reported to induce cell death *via* induction of necrosis, unprogrammed cell death in response to overwhelming chemical or physical stimuli, resulting in damage to the cell membrane.^20,22^ To probe necrotic mechanisms, the release of lactate dehydrogenase (LDH), a cytosolic enzyme, was measured Fig. 2B. A significant increase in LDH release was observed through the incorporation of an Ac_4_ManNAz pre-treatment compared to unmodified cells. The LDH release curves (Fig. 2B) similarity to the dose–response curves from the cell viability resazurin assays (Fig. S17†) supports the hypothesis that loss of membrane integrity is a primary cause of cell death, as expected.^39,40^ Interestingly, in several cases LDH release for Ac_4_ManNAz untreated cells plateaued at lower levels compared to Ac_4_ManNAz treated cells, even at higher DBCO-pDMAEMA_*n*_ concentrations, supporting our hypothesis that electrostatic interactions alone limit the effective concentration of chemotherapeutic macromolecules at the cell surface compared to those subjected to MOE.

To further probe necrosis, cells treated with or without Ac_4_ManNAz (40 μM, 96 h) and DBCO-pDMAEMA_100/150_ (0–250 μg mL^−1^, 2.5 h) were stained with calcein (green, healthy, intact membrane) and ethidium iodide (EI, red, dead, damaged membrane) and imaged, Fig. 2C (additional images in Fig. S21–S22†). Dose–response curves were generated from the percentage of live cells counted, Fig. 2C, and EC_50_ values were determined, Table 1. A significant increase in EI positive cells was observed with an Ac_4_ManNAz pre-treatment at equal DBCO-DMAEMA_100/150_ concentrations, especially with DBCO-150 which decreased the EC_50_ values by 75.8% on average across all cell types (see Table S5† for all values). Similar to the previous assays, through careful selection of DBCO-150 concentrations the number of EI positive cells across all cell types can be minimised in ‘normal’ untargeted cells to achieve an average of 80% live cells verses just 15% for Ac_4_ManNAz treated cells, revealing targeted cytotoxicity towards azido glycan labelled cells. The similarities between the EC_50_ values from live/dead staining and resazurin assays, Table 1, confirm that cell surface lysis is a major contributor to the enhancement in cell death caused by our chemotherapeutic macromolecular system, a feature that could be exploited for enhanced gene transfection or delivery of small molecule chemotherapeutics (a concept currently being investigated to enhance drug effects in MDR cells).^15^

### Additional mechanism of cell death enhanced by MOE

**Fig. 3:**
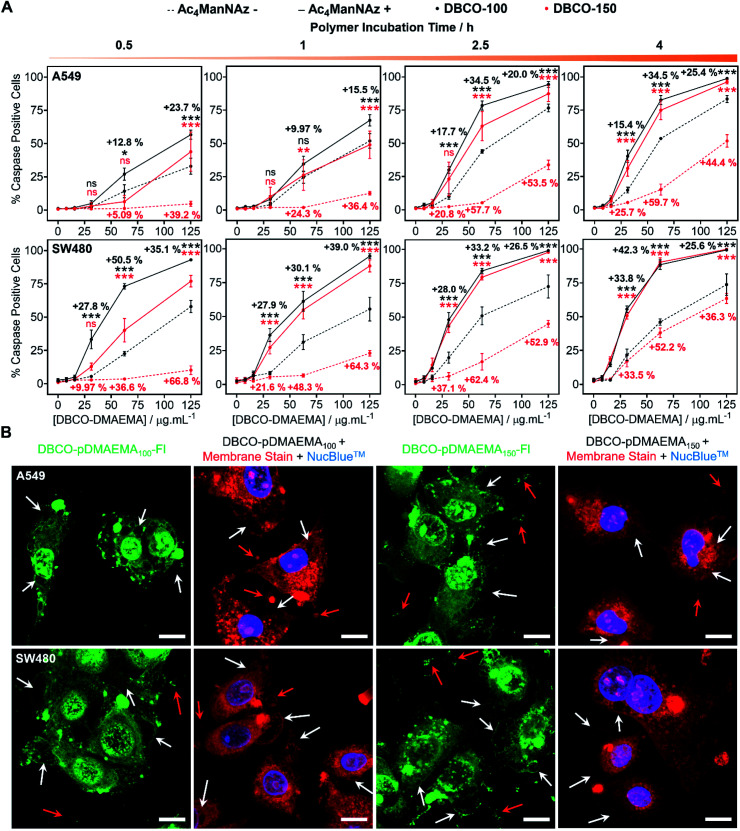
Apoptosis measurements. (A) Caspase activation of A549 and SW480 cells treated with (+) and without (−) Ac_4_ManNAz (40 μM, 96 h) and DBCO-pDMAEMA_*n*_ (0–125 μg mL^−1^, 0.5–4 h) was imaged in real-time and the average percentage of caspase positive cells ± SEM of 4 biological repeats was determined (ANOVA, Tukey PostHoc; ns: *p* ≥ 0.05, *p ≤ 0.05, **p ≤ 0.01, ***p ≤ 0.001). (B) Confocal images of A549 and SW480 cells treated with Ac_4_ManNAz (40 μM, 96 h) and DBCO-pDMAEMA_100/150_–Fl (125 μg mL^−1^, 2.5 h, green) were taken along with cells treated with Ac_4_ManNAz (40 μM, 96 h), DBCO-pDMAEMA_100/150_ (125 μg mL^−1^, 2.5 h), a nuclear stain (NucBlue™, blue) and CellMask™ membrane stain (red). Apoptotic features including blebbing (white arrows), apoptotic bodies and cell debris (red arrows) have been identified. Scale bar = 10 μm.

pDMAEMA has been reported to induce both necrosis and apoptosis (programmed cell death), although this varies between cell type.^38,41^ Therefore, determining if our MOE recruitment approach promotes and/or enhances these pathways must be explored. To probe this, A549, SW480 and MCF-7 cells were treated with or without Ac_4_ManNAz (40 μM, 96 h), incubated with DBCO-pDMAEMA_*n*_ and immediately stained with Annexin V-FITC and propidium iodide (PI), a standardised test for apoptosis (Fig. S24–S26†). A small portion of cells were Annexin V positive, the marker for early apoptosis (externalisation of phosphatidylserine), whereas the vast majority of cells were both Annexin V-FITC and PI positive, indicative of either late apoptosis or necrotic cell death. Whilst this is a standard test for apoptosis, there is a risk of false positive/negative results as PI uptake can be aided by the polycation induced membrane damage, and hence additional tests were conducted. Polycations can induce apoptosis through perturbation of the mitochondria, resulting in release of cytochrome *c* (Cyt c) and subsequent induction of executioner caspases (cysteinyl aspartate-specific proteases), such as caspase-3/7.^42,43^ To investigate this, the activation of caspase-3/7 in A549 and SW480 cell lines pre-treated with and without Ac_4_ManNAz was imaged in real-time whilst incubated with DBCO-pDMAEMA_100/150_ polymers (0–125 μg mL^−1^, 0–4 h) (images in Fig. S27–S30†) and the percentage of caspase positive cells was determined to confirm the presence of apoptosis over time, Fig. 3A. Rapid caspase activation was observed within 30 min at DBCO-pDMAEMA_*n*_ concentrations above 31.3 μg mL^−1^ for A549 cells and 15.6 μg mL^−1^ for SW480 cells, regardless of polymer chain length, which confirms the induction of apoptosis and supports the hypothesis that DBCO-pDMAEMA_100/150_ damages the cell membrane resulting in cytosolic internalisation and damage to membrane-bound organelles such as the mitochondria and potentially lysosomes, endoplasmic reticulum and golgi apparatus.^34^ The percentage of caspase positive cells increased over the 4 h polymer incubation period. Similar to the previous assays (in Fig. 2), caspase activation was significantly enhanced through the incorporation of cell surface azido glycans at all time points measured, but particularly at 2.5 h incubation (same as previous assays) and with DBCO-150 (62.5 μg mL^−1^, 2.5 h) where the average number of caspase positive cells (both A549 and SW480) increased from 11% to 71%.

EC_50_ values calculated from the percentage of caspase positive cells following 2.5 h incubation with DBCO-pDMAEMA_100/150_, Table 2, were higher than those reported for the previous cell viability assay, Table 1. The discrepancies between EC_50_ values of the different assays suggest that both the induction of necrosis, through chemical stress exerted to the cell membrane, and apoptosis-mediated pathways contribute to cell death. In addition, higher concentrations of DBCO-pDMAEMA_100/150_ may also be required to induce apoptosis due to the requirement of higher cytosolic concentrations compared to necrosis which depend on extracellular concentrations. Regardless, the caspase EC_50_ values of both DBCO-100 and DBCO-150 were decreased towards Ac_4_ManNAz treated cells confirming that MOE can be used to enhance multiple cell death pathways induced by DBCO-pDMAEMA_*n*_ polymers and potentially minimise drug resistance development.

**Table 2:** Caspase EC_50_ values of DBCO-DMAEMA_*n*_ with (+) and without (−) Ac_4_ManNAz pre-treatment. Average EC_50_ values from caspase dose–response curves have been reported ± SEM of 4 biological repeats

Polymer	EC_50_ (μg mL^−1^)			
	A549		SW480	
	−N_3_	+N_3_	−N_3_	+N_3_
DBCO-100	72.4 ± 4.7	41.5 ± 3.0	68.0 ± 5.2	32.5 ± 1.6
DBCO-150	160.9 ± 10.7	54.4 ± 4.5	134.4 ± 4.2	34.6 ± 2.6

Confocal imaging of A549 and SW480 cells treated with Ac_4_ManNAz and DBCO-pDMAEMA_100/150_–Fl (125 μg mL^−1^, 2.5 h) was undertaken, Fig. 3B. Although polymer was visualised throughout the whole cell bodies, higher regions of fluorescence intensity was found co-localised with undamaged cell membrane remains and apoptosis-associated morphological changes such as blebs, apoptotic bodies and cell debris; additional confocal images can be found in the ESI† of lower DBCO-pDMAEMA_100/150_–Fl concentrations, Fig. S33 and S34.† CellMask™ Deep Red plasma membrane staining was used to confirm the presence of membrane damage and the previous morphological changes. Polymer untreated cells showed highly localised membrane staining of the peripheral membrane, Fig. S35,† however polymer incubation resulted in extensive membrane damage leading to intracellular dye uptake, Fig. 3B. Thus, these findings further confirm that DBCO-pDMAEMA_100/150_ induces both necrotic and apoptotic pathways and can be modified to produce a dual functional imaging and cytotoxic agent which is crucial in the further development of trackable polycationic chemotherapeutic agents.

### Two-step MOE chemotherapeutic approach on spheroids

**Fig. 4:**
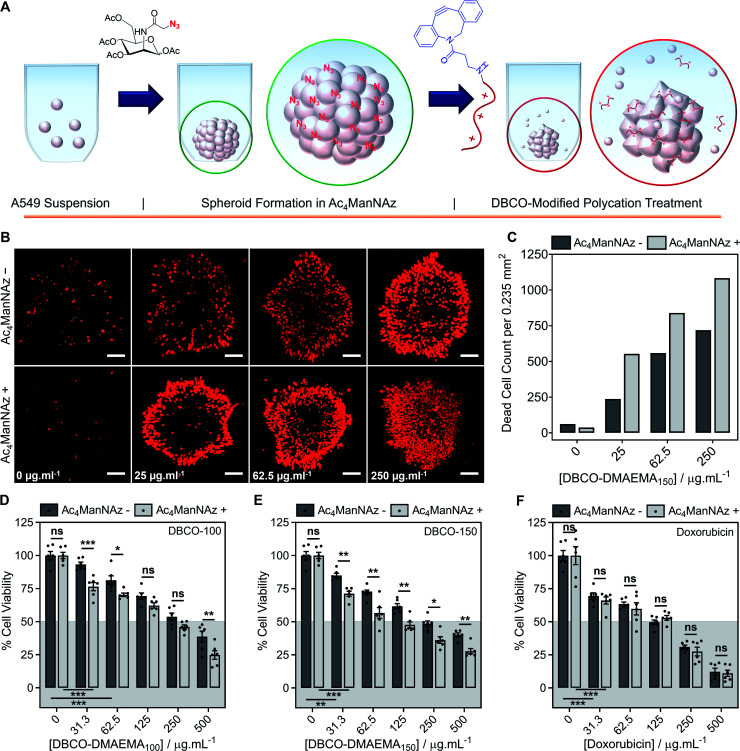
Application of chemotherapeutic macromolecules to a 3-D tumour model azido-labelled during spheroid formation. (A) A549 spheroids were formed in the presence (+) or absence (−) of Ac_4_ManNAz (40 μM, 96 h) and treated with single dose of DBCO-pDMAEMA_150_ (3 h). (B) Dead cells were stained with CellTox™ (Red) and imaged. (C) The number of dead cells per average spheroid area (0.235 mm^2^) was calculated from the confocal images. Spheroid cell viability was determined by an ATP-based assay (CellTiter-Glo®) of spheroids formed in the presence (+) and absence (−) of Ac_4_ManNAz (40 μM, 96 h) followed by either (D) DBCO-100 (3 h), (E) DBCO-150 (3 h) or (F) doxorubicin (24 h) treatment (0–500 μg mL^−1^). The data is represented by mean ± SEM of 6 biological repeats (ANOVA, Tukey PostHoc; ns: *p* ≥ 0.05, *p ≤ 0.05, **p ≤ 0.01). Scale bar = 100 μm.

Encouraged by the above observations that covalent recruitment of chemotherapeutic polycations to cell surface glycans leads to enhanced chemotherapeutic activity, *via* both membrolytic and non-membrolytic processes, a more complex 3-D cellular system was explored. Cell monolayers do not sufficiently recapitulate tumour microenvironments, which alters cellular behaviours, nor the additional challenge of permeation. A549 spheroids were formed in the presence and absence of Ac_4_ManNAz (40 μM, 96 h), Fig. 4A, with an average diameter of 595 ± 23 μm, which is within the size range capable of mimicking various properties of human solid tumours including hypoxic and acidic environments, which in turn influences cell cycle arrest, energy metabolism and the mechanism of small molecule chemotherapeutics that rely on oxygen species as their dominant mechanism of cell death.^44,45^ Confirmation of successful labelling of spheroids with azido glycans was achieved by confocal microscopy with a DBCO-Cy3 dye, Fig. S44.† Spheroids formed in the presence and absence of Ac_4_ManNAz were subsequently treated with a single dose of DBCO-150 (0–250 μg mL^−1^, 3 h), stained with CellTox™ reagent, imaged and counted, Fig. 4B and C; spheroid treatments with DBCO-100 and lysis controls can be found in the ESI (Fig. S46–S48†). [Images were recoloured to show dead cells as red. Original images are in Fig. S48†]. Confocal imaging revealed a clear increase in the number of dead cells in spheroids treated with Ac_4_ManNAz with the use of both DBCO-100 and DBCO-150. Spheroid cell death counts increased by an average of ∼1.8-fold when MOE recruitment of DBCO-150 was used and the largest increase (2.3-fold) was noted at the lowest concentration tested, 25 μg mL^−1^. As expected from a single-dose treatment, the spheroid periphery was more damaged than the interior due to the size of the spheroid and absence of flux and additional fluid recycling mechanisms; however, this is expected to improve with multiple polymer treatment dosages and with the addition of *in vivo* circulatory networks. Conventional small molecule chemotherapeutics are also unable to damage the entirety of spheroids of these dimensions through single treatments,^46,47^ thus these findings are highly promising and comparable to clinically-relevant pharmaceutical compounds.

An ATP cell viability assay was employed to determine the overall cell death of spheroids formed in Ac_4_ManNAz supplemented media or unsupplemented media and subjected to a single dose of DBCO-100 and DBCO-150 (0–500 μg mL^−1^, 3 h) compared to untreated ‘healthy’ spheroids, Fig. 4D and E. A significant decrease in spheroid viability was observed at all DBCO-150 concentrations by incorporating an Ac_4_ManNAz pre-treatment, however high concentrations (62.5 μg mL^−1^ or above) were required to penetrate the core and reduce cell viability below 55%; confirming that low concentrations of DBCO-pDMAEMA_*n*_ results in cell death localised to the spheroid surface in the absence of flux mechanisms. Spheroids produced with and without Ac_4_ManNAz were also treated with doxorubicin (0–500 μg mL^−1^) for 24 h to compare our chemotherapeutic system against a conventional chemotherapeutic small molecule, Fig. 4F. DBCO-100 and DBCO-150 polymers were able to decrease the cell viability of spheroids to the same extent as doxorubicin between 62.5 and 250 μg mL^−1^ in far less time (from 24 h to 3 h) once azido glycans were incorporated to the cell surface for covalent recruitment. DBCO-pDMAEMA_100/150_'s ability to exploit both necrosis- and apoptosis-mediated cell death pathways contributed to the enhanced spheroid penetration depth at low incubation times and could be exploited in combination with small molecule chemotherapeutics to aid in increasing spheroid core delivery.^20^ Thus, we have demonstrated that our two-step MOE and chemotherapeutic macromolecular system enhances the cytotoxic effects of polycationic cytotoxic agents towards, not only target 2-D labelled cells but also 3-D tumour models.

**Fig. 5:**
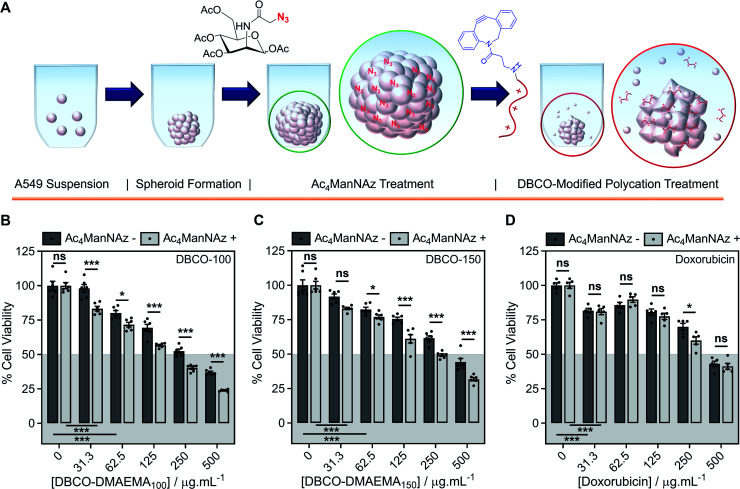
Application of chemotherapeutic macromolecules to a 3-D tumour model azido-labelled after spheroid formation. (A) A549 spheroids were pre-formed for 72 h before treatment with (+) and (−) without Ac_4_ManNAz (40 μM, 96 h), followed by a single dose of chemotherapeutic macromolecule. Spheroid cell viability was determined by an ATP-based assay (CellTiter-Glo®) of A549 spheroids treated with (+) and without (−) Ac_4_ManNAz followed by either (B) DBCO-100 (3 h), (C) DBCO-150 (3 h) or (D) doxorubicin (24 h) treatment (0–500 μg mL^−1^). The data is represented by mean ± SEM of 6 biological repeats (ANOVA, Tukey PostHoc; ns: *p* ≥ 0.05, *p ≤ 0.05, **p ≤ 0.01). Scale bar = 100 μm.

To further recapitulate a tumour and *in vivo* microenvironment, and remove concerns over azido glycan incorporation potentially influencing spheroid packing due to alterations in cell–cell contact, pre-formed spheroids (72 h) were treated with Ac_4_ManNAz (40 μM, 96 h) and subjected to a single dose of DBCO-100 and DBCO-150 (0–500 μg mL^−1^, 3 h), Fig. 5A. Despite the increase in spheroid size and contraction, treatment of pre-formed spheroids with Ac_4_ManNAz resulted in azido glycan incorporation throughout the spheroid, confirmed by confocal microscopy with a DBCO-Cy3 dye, Fig. S45.† As before, an ATP cell viability assay was used to determine overall spheroid cell death following combinatorial treatment of Ac_4_ManNAz and DBCO-100 or DBCO-150, Fig. 5B–D. In comparison to Ac_4_ManNAz untreated spheroids, a significant increase in spheroid cell death was observed using both polymers at almost all concentrations tested. Despite the increased size of the spheroid, the DBCO-modified polycations exhibited minimal decrease in activity at higher concentrations, ≥62.5 μg mL^−1^ for DBCO-100 and ≥125 μg mL^−1^ for DBCO-150, whereas doxorubicin's performance diminished substantially compared to the previous spheroids. Thus, treatment of spheroids with Ac_4_ManNAz post-formation does not appear to impede MOE labelling capabilities and chemotherapeutic macromolecular functionality. We anticipate multiple doses of polymer would lead to even larger effects.

## Discussion

A strategy to significantly enhance the therapeutic potential of chemotherapeutic macromolecules has been established by exploiting metabolic oligosaccharide engineering (MOE) of cancerous cells, to covalently recruit chemotherapeutic polymers *via* bio-orthogonal reactions. The covalent recruitment strategy was designed to overcome the intrinsic cytotoxicity of AMP/HDP polymer mimics by ensuring that, at non-toxic concentrations, they selectively kill labelled cells by triggering and enhancing multiple mechanisms of cell death. This is significant as current approaches to increase the therapeutic window of macromolecular chemotherapeutics have focussed on iterative tuning of polymer hydrophilicity/phobicity, rather than chemically tuning the cellular target.

Strained alkyne-terminated poly(dimethylaminoethyl methacrylate) (DBCO-pDMAEMA) was used as the prototypical cationic macromolecular therapeutic. DBCO-pDMAEMA was covalently recruited, by strain-promoted click, onto azido-glycans on tumour cell surfaces, selectively introduced by MOE using Ac_4_ManNAz. Against a panel of MOE treated cell lines, DBCO-pDMAEMA's activity increased up to 10-fold (*i.e.* decrease in EC_50_ value) compared to untreated cells. Conditions were identified where >90% of unlabelled cells remained viable whereas only <20% of labelled cells did; thus, the ‘therapeutic window’ (*i.e.* selectivity) of the polycationic chemotherapeutic was increased without changing the nature of the therapeutic itself. Traditional macromolecular chemotherapeutics (polycations) typically kill cells by necrosis caused by electrostatic interactions/disruption of anionic membrane components. The covalent recruitment strategy employed induced necrotic and apoptotic mechanisms of cell death associated with organelle damage, hypothesised to be promoted by polycation internalisation following membrane damage and/or *via* glycan recycling pathways. Targeting multiple cell death pathways may help avoid future resistance development.

Finally, our two-step MOE and chemotherapeutic system was applied to 3-D spheroids models intended to more accurately reproduce an *in vivo* tumour environment. Spheroids formed from pre-labelled cells lead to significantly enhanced cell death, compared to untreated cells, when DBCO-pDMAEMA was applied demonstrating the potential of this covalent capture approach. Secondly, spheroids were initial formed in the absence of Ac_4_ManNAz and then metabolically labelled to take into account any defects in spheroid packing due to cell–cell contact alterations and assess whether spheroids can be labelled with azido-glycans post formation, which is closer to what might be applied *in vivo*. Again, enhanced cell death was observed in MOE treated pre-formed spheroids from a single dose of polymer. The increased size of the Ac_4_ManNAz treated pre-formed spheroid did not impede polycationic activity at higher concentrations, whereas doxorubicin's performance was diminished. Thus, DBCO-pDMAEMA's ability to exploit both necrosis- and apoptosis-mediated cell death pathways could be exploited in combination with small molecule chemotherapeutics to aid in increasing spheroid core delivery.

The universal nature of this platform may overcome the long-standing selectivity issues associated with synthetic chemotherapeutic macromolecules derived from host defence peptides. Engineering the target cells to recruit chemotherapeutic polycations, rather than just engineering of polymers, offers a conceptually new approach to improve their efficacy and will help advance the discovery and application of polymeric therapeutics as active agents, rather than just as carriers.

## Methods

For complete experimental methods see the ESI.†

### Cell culture

Human Caucasian lung carcinoma cells (A549), human Caucasian Dukes' type B colorectal adenocarcinoma cells (SW480), and human Caucasian breast adenocarcinoma derived from metastatic site (MCF-7) were grown in Ham's F-12K (Kaighn's) medium (F-12K), advanced Dulbecco's Modified Eagle's Medium (advanced DMEM) and Dulbecco's Modified Eagle's Medium-high glucose (DMEM-high glucose), respectively, supplemented with 10% fetal bovine serum (FBS) and 100 units per mL penicillin, 100 μg mL^−1^ streptomycin, and 250 ng mL^−1^ amphotericin B (PSA). Cells were incubated at 37 °C and 5% CO_2_ and passaged every 3–4 days, before reaching 70–80% confluency. Cells were dissociated using a balanced salt solution containing trypsin (0.25%) and EDTA (1 mM).

### General protocol for metabolic glycan labelling

A549, SW480 and MCF-7 cells were seeded in 100 × 20 mm TC-treated cell culture dishes in complete cell media, as described above, supplemented with *N*-azidoacetylmannosamine-tetraacylated (Ac_4_ManNAz, 40 μM) in a humidified atmosphere of 95% air and 5% CO_2_ at 37 °C for 72 h. Azido modified cells were dissociated using Accutase® solution for subsequent experiments.

### Polymer synthesis

#### Synthesis of PFP-50–PFP-150

2-(Dimethylamino)ethyl methacrylate (DMAEMA) (2.00 g, 12.7 mmol), 2-(dodecylthiocarbonothioylthio)-2-methylpropionic acid pentafluorophenyl ester (PFP-DMP) and 4,4′-azobis(4-cyanovaleric acid) (ACVA) were dissolved in dioxane (10 mL) at ratios presented in Table S1† to obtain 3 degrees of polymerization (DP). Mesitylene (150 μL) was used as an internal reference and an aliquot was taken in CDCl_3_ for NMR analysis. The reaction mixture was stirred under N_2_ for 30 min at RT and a further 16 h at 70 °C. An aliquot of the post-reaction mixture was taken for NMR analysis in CDCl_3_, allowing percentage conversion calculations. The polymer was reprecipitated into hexane from THF three times yielding a yellow polymer product, PFP-DMAEMA_*n*_ (PFP-50–PFP-150). The resulting product was dried under vacuum and DMF SEC and ^1^H, ^13^C and ^19^F NMR (CDCl_3_) analysis was completed.

#### Synthesis of DBCO-50–DBCO-150

PFP-p(DMAEMA)_*n*_ (0.20 g, 1 eq.), and dibenzocyclooctyne-amine (DBCO-NH_2_; 2 eq.) were stirred in THF (3 mL) for 16 h. Subsequent addition of propyl amine (1.5 eq.) for 2 h was used to ensure complete reduction of the thiocarbonate moiety to a thiol group. The polymer was reprecipitated into cold hexane from THF three times yielding a white polymer product, DBCO-DMAEMA_*n*_ (DBCO-50–DBCO-150). The resulting product was dried under vacuum and DMF SEC and ^1^H, ^13^C and ^19^F NMR (CDCl_3_) analysis was completed.

#### Synthesis of FL-100 and FL-150

DBCO-p(DMAEMA)_*n*_ (0.10 g, 1 eq.), and *N*-(5-fluoresceinyl)maleimide (1.5 eq.) were dissolved in DMF (2 mL), degassed and left to stir for 24 h. The yellow mixture was reprecipitated into cold hexane from THF (×3), yielding a yellow fluorescent polymer product. DMF SEC analysis was completed with the UV-Vis detector set at 494 nm to demonstrate size separation and absorbance overlap. All characterisation data can be found in the ESI.†

### Polymer chemotherapeutic treatment and viability studies

A549 and SW480 cells, treated and untreated with Ac_4_ManNAz (as described above), were seeded in a 96 well plate at a density of 5k cells per well and MCF-7 cells at a density of 15k cells per well, all in the presence and absence of Ac_4_ManNAz (40 μM) for 24 h. Following DPBS washes (×3), all cells were incubated with complete media supplemented with DBCO-p(DMAEMA)_50–150_ (0–250 μg mL^−1^) for 2.5 h. Chemotherapeutic polymer solutions were replaced with fresh complete media, following DPBS washes (×3), and cells were incubated for 24 h. Cell viability was determined by replacing all solutions with alamarBlue® reagent (10% v/v in cell media). Absorbance measurements were obtained at 570 nm and 600 nm every 30 min/1 h for 4 h to monitor the reduction of resazurin to resorufin by viable cells. Control cells untreated and treated with Ac_4_ManNAz were also treated with alamarBlue® solution to provide a maximum resazurin reduction value of viable cells, along with background measurements of alamarBlue® reagent alone (10% v/v in cell media). Percentage cell viability was reported relative to the respective viable cell controls, either cells untreated or treated with Ac_4_ManNAz. Five biological repeats were completed.

### Membrane permeability and leakage assay

A549 and SW480 cells, treated and untreated with Ac_4_ManNAz (as described above), were seeded in a 96 well plate at a density of 10k cells per well and MCF-7 cells at a density of 15k cells per well, all in the presence and absence of Ac_4_ManNAz (40 μM) for 24 h. Following DPBS washes (×3), all cells were incubated with complete media supplemented with DBCO-p(DMAEMA)_100/150_ (0–250 μg mL^−1^) for 2.5 h. Lysis controls were also prepared (see ESI†) for Ac_4_ManNAz treated and untreated cells to determine maximum lactate dehydrogenase (LDH) release. The supernatants of polymer treated cells, the lysis controls and untreated negative controls were transferred to a separate 96 well plate (50 μL) and LDH reaction mixture (50 μL) was added to the supernatants (50 μL) for 30 min at RT. Stop solution (50 μL), provided by the Thermo Scientific™ Pierce™ LDH Cytotoxicity Assay Kit, was added and absorbance readings were recorded at 490 nm and 680 nm to monitor active LDH release from plasma membrane damage and as a background subtraction measurement, respectively. Three biological repeats were completed.

### Live/dead staining

A549 and SW480 cells, treated and untreated with Ac_4_ManNAz (as described above), were seeded in a 96 well plate at a density of 10k cells per well and MCF-7 cells at a density of 15k cells per well, all in the presence and absence of Ac_4_ManNAz (40 μM) for 24 h. Following DPBS washes (×3), all cells were incubated with complete media supplemented with DBCO-p(DMAEMA)_50–150_ (0–250 μg mL^−1^) for 2.5 h. The polymer solutions were removed, cells washed with DPBS (×3) and incubated in complete media for 24 h. Cells were stained with ethidium iodide (2 μM) and calcein (2 μM) in DPBS (100 μL) for 40 min at RT. Ac_4_ManNAz untreated and treated cells were also stained as control viable cell samples and all cells were imaged using phase contrast and blue (calcein) and green (ethidium) excitation lasers. Cells were counted using ImageJ and values were reported as percentage live cells relative to the total number of cells (four biological repeats were completed).

### Caspase-3/7 kinetics apoptosis assay

A549 and SW480 cells, treated and untreated with Ac_4_ManNAz (as described above), were seeded in a 96 well plate at a density of 10k cells per well, in the presence and absence of Ac_4_ManNAz (40 μM) for 24 h. Following DPBS washes (×3), the cells were incubated with complete media supplemented with CellEvent Caspase-3/7 Detection Reagent (5 μM) and DBCO-p(DMAEMA)_100/150_ (0–125 μg mL^−1^). Images were taken at 0.5, 1, 2.5 and 4 h time intervals with a phase contrast channel and a blue excitation laser. Ac_4_ManNAz untreated and treated cells were also incubated with CellEvent Caspase-3/7 Detection Reagent (5 μM) as control viable cells. Cells were counted using ImageJ and values were reported as percentage caspase positive cells relative to the total number of cells. Four biological repeats were completed.

### Fluorescent chemotherapeutic polymer and membrane stain imaging

Ac_4_ManNAz treated and untreated A549 and SW480 cells (5k cells) were seeded in CELLview™ Culture dishes in the presence and absence of Ac_4_ManNAz (40 μM) and allowed to attach for 24 h. Cells were washed with DPBS (×3) and subsequently incubated with DBCO-p(DMAEMA)_100/150_–Fl for 2.5 h. Polymer solutions were removed and replaced with Leibovitz's L-15 Medium with no phenol red, following ×3 DPBS washes. Z-stack confocal imaging was completed immediately afterwards with a 63× objective lens, 488 nm excitation laser and emission filters set to 493–634 nm; see ESI† for complete confocal parameters. Cells were also imaged following treatment with DBCO-p(DMAEMA)_100/150_ (2.5 h) and staining with CellMask™ Deep Red Plasma membrane Stain (25 μg mL^−1^, 100 μL, 30 min, RT) and NucBlue® Live Cell ReadyProbes® Reagent (5 min at RT) in L15 media with no phenol red, with DPBS washes (×3) between steps. Z-stack confocal imaging was completed with a 63× objective lens, 405 nm and 633 nm excitation laser and emission filters set to 410–483 nm and 638–747 nm. Maximum intensity projection images and 3D reconstructions were produced using Zen 2.3.

### Spheroid formation and metabolic oligosaccharide engineering

MOE of A549 spheroids was achieved using 2 approaches, (1) formation in Ac_4_ManNAz and (2) Ac_4_ManNAz treatment of pre-formed spheroids. For approach (1), A549 cells were seeded (2k cells per well) in an ultralow attachment U-bottom plate in the presence and absence of Ac_4_ManNAz (40 μM) supplemented F-12K complete media. The plate was centrifuged at 2k RPM for 10 min and spheroids were allowed to grow for 96 h in a humidified atmosphere of 95% air and 5% CO_2_ at 37 °C. For approach (2), A549 cells were seeded (1k cells per well) in an ultralow attachment U-bottom plate, in F-12K complete media, centrifuged at 2k RPM for 10 min and allowed to grow for 72 h. Following 72 h, the media was replaced with either complete fresh media (no Ac_4_ManNAz) or complete fresh media supplemented with Ac_4_ManNAz (40 μM) and incubated for another 96 h in a humidified atmosphere.

### Spheroid polymer treatment and viability assay

Azido glycan labelled and unlabelled spheroids, using both approaches described above, were incubated with either DBCO-p(DMAEMA)_100/150_ (0–500 μg mL^−1^, 100 μL) for 3 h or doxorubicin (0–500 μg mL^−1^, 100 μL) for 24 h. Following DPBS washes (×3), the chemotherapeutic solutions were replaced with fresh media and an equal volume of CellTiter-Glo® (100 μL) was subsequently added. The U-bottom plate was placed on a plate shaker for 10 min to ensure lysis and allowed to equilibrate for 25 min at RT. The resulting solutions were transferred into an opaque white plate to record luminescence measurements. Six biological repeats were completed.

### Spheroid cytoxicity imaging

Spheroids formed in the presence and absence of Ac_4_ManNAz (approach (1) above) were incubated with DBCO-p(DMAEMA)_100/150_ (31.3, 62.5 and 250 μg mL^−1^) for 3 h. Following DPBS washes (×3), the polymer solutions were replaced with fresh media and an equal volume of CellTox™ Green reagent (100 μL, 2× concentration) for 15 min at RT. Spheroids were centrifuged at 2k RPM for 5 min and Z-stack confocal imaging was completed using a 10× objective lens, a 488 nm excitation laser and emission filters set to 493–634 nm. Spheroids untreated and treated with Ac_4_ManNAz, but untreated with chemotherapeutic polymer solutions, were also stained with CellTox™ Green reagent as negative controls and lysis controls were prepared as positive controls; see ESI.† Maximum intensity projection images and 3D reconstructions were produced using Zen 2.3. Note: dead cells were converted from green to red.

## Conflicts of interest

There are no conflicts to declare.

## Supplementary Material

SC-012-D0SC06580C-s001
