# Supplementary material for: Covalent cell surface recruitment of chemotherapeutic polymers enhances selectivity and activity
Source: Chem Sci. 2021 Feb 12;12(12):4557–69. doi: 10.1039/d0sc06580c (PMC8179505; doi:10.1039/d0sc06580c)
Supplement: SC-012-D0SC06580C-s001 [file SC-012-D0SC06580C-s001.pdf]

Electronic Supplementary Material (ESI) for Chemical Science.
This journal is © The Royal Society of Chemistry 2021

Supporting Information

Covalent Cell Surface Recruitment of Chemotherapeutic Polymers Enhances Selectivity and Activity

Ruben M. F. Tomás[a,c] and Matthew I. Gibson[a,b*]

[a] *Department of Chemistry, University of Warwick, Gibbet Hill Road, Coventry, CV4 7AL, UK;*

[b] *Warwick Medical School, University of Warwick, Gibbet Hill Road, Coventry, CV4 7AL, UK;*

[c]*MAS CDT, Senate House, University of Warwick, Coventry CV4 7AL*

*Corresponding Author m.i.gibson@warwick.ac.uk

1 Experimental

1.1 Materials

2-(Dimethylamino)ethyl methacrylate (DMAEMA), 4,4′-azobis(4-cyanovaleric acid) (ACVA), dioxane, hexane, tetrahydrofuran (THF), mesitylene, dibenzocyclooctyne-amine ($DBCO-NH_2$), propyl amine, N-(5-fluoresceinyl)maleimide (≥90%, HPLC grade), chloroform-d ($CDCl_3$, 99.8%), doxorubicin hydrochloride (98%), Triton X-100 and sucrose were purchased from Sigma Aldrich Co Ltd (Gillingham, UK). Dulbecco phosphate buffered saline (DPBS), N-azidoacetylmannosamine-tetraacylated ($Ac_4ManNAz$), NucBlue® Live Cell ReadyProbes® Reagent, alamarBlue™ Cell Viability Reagent, LIVE/DEAD™ Viability/Cytotoxicity Kit for mammalian cells, CellEvent Caspase-3/7 Detection Reagent, CellMask™ Deep Red Plasma membrane stain, Molecular Probes™ Dead Cell Apoptosis Kit with Annexin V FITC and PI, Thermo Scientific™ Pierce™ LDH Cytotoxicity Assay Kit, sodium hydroxide, magnesium chloride and Tris-HCl were purchased from Fisher Scientific (Loughborough, UK). CellTox™ Green Cytotoxicity Assay and CellTiter-Glo® 3D Luminescent Cell Viability Assay were purchased from Promega (Wisconsin, USA). CELLview™ Culture dish, 35 mm, TC treated, 4 compartments were purchased from Greiner Bio-One (Stonehouse, UK). Ovine blood was purchased from TCS Biosciences (Buckingham, UK). All reagents were used as purchased, without further purification.

1.2 Physical and Analytical Methods

Size Exclusion Chromatography (SEC). An Agilent Infinity II MDS instrument equipped with differential refractive index (DRI), viscometry (VS), dual-angle light scatter (LS) and variable wavelength UV-Vis detectors (set at 494 nm) was used for all SEC analysis. The system was fitted with 2 x PLgel Mixed D columns (300 x 7.5 mm) and a PLgel 5 µm guard column. The

eluent utilised was DMF with 5 mmol NH_4BF_4 additive at a flow rate of 1.0 mL.min^{-1} at 50 °C. Poly(methyl methacrylate) standards (Agilent EasyVials) were used for calibration between 955,000 – 550 g.mol^{-1}. Samples were prepared in the mobile phase and passed through a nylon membrane with 0.22 μm pore size prior to injection. Agilent GPC/SEC Software was used to determine experimental molar mass ($M_{n,exp}$), experimental molecular weight ($M_{w,exp}$) and dispersity (Đ) of synthesized polymers.

Nuclear Magnetic Resonance (NMR) Spectroscopy. ^{1}H and ^{19}F NMR spectra were recorded on a Bruker HD-300 spectrometer and ^{13}C NMR spectra on a Bruker HD-400 spectrometer, both operating at 293 K using deuterated solvents purchased from Sigma-Aldrich. Chemical shifts were reported as δ in parts per million (ppm) relative to residual non-deuterated solvent resonances ($CDCl_3$ ^{1}H: δ = 7.26 ppm; ^{13}C δ = 77.16 ppm). Polymer compositions and monomer conversions were determined using spectra obtained. Bruker Topspin 3.5 Software was used to process and export spectra.

Infrared (IR) Spectroscopy. FTIR spectra were acquired using an Agilent Cary 630 FTIR (Agilent Technologies, Connecticut, USA) spectrometer equipped with a single reflection diamond ATR accessory with a 45° angle of incidence, a 1 mm diameter sampling surface (200 μm active area) and a rotating pressure clamp (applying maximum pressure). Scans (128) were obtained of dried, crushed samples between 4000 - 650 cm^{-1} with a spectral resolution: < 2 cm^{-1}, wavenumber accuracy of 0.05 cm^{-1} and wavenumber reproducibility of 0.005 cm^{-1}. Gain, aperture, scan speed and filter were all set to auto. Agilent MicroLab Software, version B.05.4, was used to process and export spectra.

Confocal Microscopy. Confocal imaging was completed using a Zeiss LSM 880 inverted microscope with 100x, 63x, 40x and 20x oil immersion objective lenses, equipped with three photomultiplier detectors (GaAsP, multialkali and BiG.2) and multichannel spectral imaging

with an ultra-sensitive GASP detector. The UV and VIS Laser Modules allowed selection of six lasers with wavelengths of 633, 594, 561, 543, 514, 488, 458 and 405 nm. Zeiss ZEN (black edition) 2.3 lite was utilised for image collection and processing. All other imaging was completed using an Olympus CX41 microscope equipped with a UIS-2 20x/0.45/∞/0−2/FN22 lens (Olympus Ltd., Southend-on-Sea, U.K.) and a Canon EOS 500D SLR digital camera.

UV-Vis Spectroscopy. An Agilent Technologies Cary 60 Variable Temperature UV-Vis spectrometer was used to record absorbance measurements of DBCO-DMAEMA$_{100/150}$-Fl polymers, for polymer: dye ratio calculations, between 300 and 800 nm at a scan rate of 600 nm.min^{-1} and 1 nm data interval. Dual beam mode was used and the system was operated using Agilent software.

Statistical Analysis. Data was analysed with a one-way analysis of variance (ANOVA) on ranks followed by a comparison of experimental groups with the appropriate control group (Tukey's post hoc test). GraphPad Prism 8 (La Jolla, California, USA) was used for all statistical analysis including the plotting and determination of EC_{50} and EC_{90} values.

1.3 General Cell Culture

Cell Lines. Human Caucasian lung carcinoma cells (A549, Cat. No. 86012804), human Caucasian Dukes' type B, colorectal adenocarcinoma cells (SW480, Cat. No. 87092801), and human Caucasian breast adenocarcinoma derived from metastatic site (MCF-7, Cat. No. 86012803) were obtained from European Collection of Authenticated Cell Cultures (Public Health England, UK).

Cell Culture. A549 cells were grown in 175 cm^2 Nunc cell culture flasks (ThermoFisher, Rugby, UK) with Ham's F-12K (Kaighn's) Medium (F-12K) (Gibco, Paisley, UK) supplemented with 10% USA-origin fetal bovine serum (FBS) purchased from Sigma Aldrich (Dorset, UK), 100 units.mL^{-1} penicillin, 100 µg.mL^{-1} streptomycin, and 250 ng.mL^{-1}

amphotericin B (PSA) (HyClone, Cramlington, UK). SW480 cells were grown in 75 cm^2 Nunc cell culture flasks (ThermoFisher, Rugby, UK) with Gibco™ Advanced Dulbecco's Modified Eagle's Medium (Gibco, Paisley, UK) supplemented with 10% FBS, 100 units.mL^{-1} penicillin, 100 µg.mL^{-1} streptomycin, and 250 ng.mL^{-1} amphotericin B. MCF-7 cells were grown in 75 cm^2 Nunc cell culture flasks with Dulbecco's Modified Eagle's Medium - high glucose (Gibco, Paisley, UK) supplemented with 10% FBS, 100 units.mL^{-1} penicillin, 100 µg.mL^{-1} streptomycin, and 250 ng.mL^{-1} amphotericin B. General maintenance of all cell lines was completed by passaging every 3 – 4 days, before reaching 70 – 80% confluency. Cells were dissociated using a balanced salt solution containing trypsin (0.25%) and EDTA (1 mM) (Gibsco) and reseeded in 175/75 cm^2 cell culture flasks at appropriate seeding densities. All azido sugar and polymer solutions were prepared in the respective cell media.

1.4 Synthesis of Azide-Reactive Chemotherapeutic Polymers

General Procedure for Polymerisation of 2-(Dimethylamino)ethyl Methacrylate

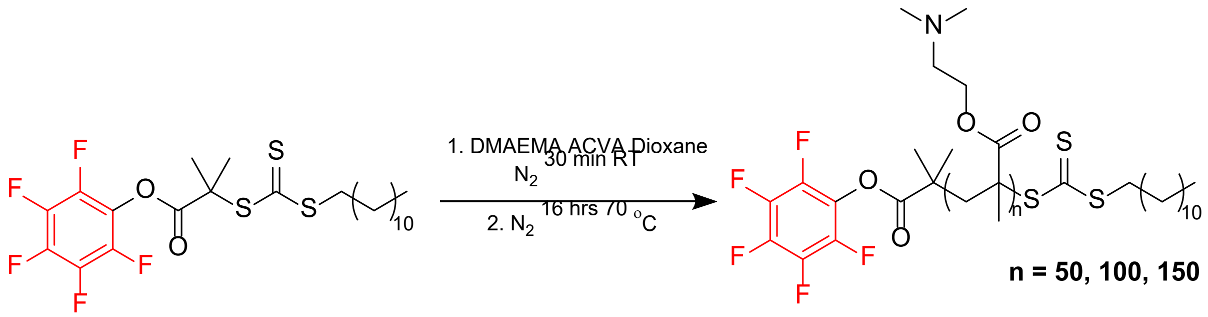

Figure S1. *Polymerisation of 2-(dimethylamino)ethyl methacrylate using PFP-DMP.*

2-(Dodecylthiocarbonothioylthio)-2-methylpropionic acid pentafluorophenyl ester (PFP-DMP) was synthesised as previously described.[1–3] 2-(Dimethylamino)ethyl methacrylate DMAEMA (2.00 g, 12.7 mmol), PFP-DMP and 4,4′-azobis(4-cyanovaleric acid) (ACVA) were dissolved in dioxane (10 mL) at ratios presented in Table S1 to obtain 3 degrees of polymerisation (DP). Mesitylene (150 µL) was used as an internal reference and an aliquot was

taken in $CDCl_3$ for NMR analysis. The reaction mixture was stirred under N_2 for 30 min at RT and a further 16 h at 70 °C. An aliquot of the post-reaction mixture was taken for NMR analysis in $CDCl_3$, allowing percentage conversion calculations. The polymer was reprecipitated into hexane from THF three times, yielding a yellow polymer product. The resulting product was dried under vacuum and an aliquot was taken for NMR analysis in $CDCl_3$. NMR percentage conversion and SEC results are presented in Table S1.

Table S1. *SEC results of PFP-pDMAEMA$_n$.*

Polymer	[M]:[CTA] (−)	%Conv. [a]	$M_{n,th}$ [b] (g.mol^{-1})	$M_{n,exp}$ [c] (g.mol^{-1})	$M_{W,exp}$ [c] (g.mol^{-1})	$Đ$ [c]
PFP-pDMAEMA$_{50}$	50	91	7683	12100	14600	1.21
PFP-pDMAEMA$_{100}$	100	95	15465	17700	21500	1.21
PFP-pDMAEMA$_{150}$	150	93	22461	24300	29200	1.20

[a] Determined by ^{1}H NMR against an internal mesitylene standard.
[b] Determined by the [M]:[CTA] ratio and conversion, assuming 100% CTA efficiency.
[c] Determined by SEC in DMF against PMMA standards.

PFP-pDMAEMA$_{50}$: ^{1}H NMR (300 MHz, $CDCl_3$) δ_{ppm}: 4.06 ppm (100H, s, **CH_2**-O); 2.57 ppm (100H, br. s, **CH_2**-N); 2.28 ppm (302H, s, N-**$(CH_3)_2$**, **H^{12}**); 2.08-1.70 ppm (106H, multiple br. s, **backbone CH_2** and **H^{13-14}**); 1.40-1.20 ppm (20H, br. s, **H^{2-11}**); 1.13-0.774 ppm (153H, **backbone CH_3**, **H^1**). Solvent peaks: 3.75 ppm and 1.83 ppm (m, THF) solvent peak and 7.26 ppm (s, $CDCl_3$). *^{19}F NMR (300 MHz, $CDCl_3$) δ_{ppm}:* -152.5 ppm (2F, d, $J_{1\text{-}2}$ = 18.6 Hz, **F^1, ortho**); -158.0 ppm (1F, t, $J_{1\text{-}2}$ = 22.8 Hz, **F^3, para**); -162.3 ppm (2F, t, $J_{1\text{-}2}$ = 20.5 Hz, **F^2, meta**). *^{13}C NMR (400 MHz, $CDCl_3$) δ_{ppm}:* 63.1 ppm (**CH_2**-O), 57.2 ppm (**CH_2**-N), 45.7 ppm (N-**$(CH_3)_2$**) and 44.8 ppm (**backbone quaternary C**). Solvent peaks: 77.04 ppm (t, $CDCl_3$). *IR ν /* cm^{-1}*:* 3025 - 2689 cm^{-1} (w, C-H stretch); 2820 cm^{-1} (w, asym. C-H stretch (N-CH_3)); 2768 cm^{-1} (w, sym. C-H stretch (N-CH_3)); 1724 cm^{-1} (s, C=O stretch); 1522 cm^{-1} (w, C-F stretch); 1455 cm^{-1} (m, C-H bend (CH_2)), 1171 cm^{-1} (s, C-O stretch); 1144 cm^{-1} (s, C-N stretch), and 1062 cm^{-1} (w, S-(C=S)-S stretch).

PFP-pDMAEMA$_{100}$: ^{1}H NMR (300 MHz, $CDCl_3$) δ_{ppm}: ^{1}H NMR (300 MHz, $CDCl_3$) δ_{ppm}: 4.05 ppm (200H, s, **CH_2**-O); 2.56 ppm (200H, br. s, **CH_2**-N,); 2.28 ppm (602H, s, N-**$(CH_3)_2$**, **H^{12}**); 2.09-1.70 ppm (206H, multiple br. s, **backbone CH_2** and **H^{13-14}**); 1.40-1.19 ppm (20H, br. s, **H^{2-11}**); 1.14-0.774 ppm (303H, **backbone CH_3**, **H^1**). Solvent peaks: 3.75 ppm and 1.85 ppm (m, THF) solvent peak and 7.27 ppm (s, $CDCl_3$). *^{13}C NMR (400 MHz, $CDCl_3$) δ_{ppm}:* 63.1 ppm (**CH_2**-O), 57.2 ppm (**CH_2**-N), 45.7 ppm (N-**$(CH_3)_2$**) and 44.8 ppm (**backbone quaternary C**). Solvent peaks: 77.04 ppm (t, $CDCl_3$). *^{19}F NMR (300 MHz, $CDCl_3$) δ_{ppm}:* -152.5 ppm (2F, d, J_{1-2} = 17.4 Hz, **F^1, ortho**); -158.0 ppm (1F, t, J_{1-2} = 22.5 Hz, **F^3, para**); -162.3 ppm (2F, t, J_{1-2} = 20.1 Hz, **F^2, meta**). *IR ν* / cm^{-1}*:* 3027 - 2693 cm^{-1} (w, C-H stretch); 2821 cm^{-1} (w, asym. C-H stretch (N-CH_3)); 2770 cm^{-1} (w, sym. C-H stretch (N-CH_3)); 1724 cm^{-1} (s, C=O stretch); 1522 cm^{-1} (w, C-F stretch); 1456 cm^{-1} (m, C-H bend (CH_2)). 1172 cm^{-1} (s, C-O stretch); 1145 cm^{-1} (s, C-N stretch), 1063 cm^{-1} (w, S-(C=S)-S stretch), 915 cm^{-1} (w, C_5F_6 associated vibration).

PFP-pDMAEMA$_{150}$: ^{1}H NMR (300 MHz, $CDCl_3$) δ_{ppm}: ^{1}H NMR (300 MHz, MeOD) δ_{ppm}: ^{1}H NMR (300 MHz, $CDCl_3$) δ_{ppm}: 4.06 ppm (300H, s, **CH_2**-O); 2.56 ppm (300H, br. s, **CH_2**-N,); 2.28 ppm (902H, s, N-**$(CH_3)_2$**, **H^{12}**); 2.09-1.72 ppm (306H, multiple br. s, **backbone CH_2** and **H^{13-14}**); 1.38-1.20 ppm (20H, br. s, **H^{2-11}**); 1.14-0.772 ppm (453H, **backbone CH_3**, **H^1**). Solvent peaks: 3.75 ppm and 1.85 ppm (m, THF) solvent peak and 7.27 ppm (s, $CDCl_3$). *^{19}F NMR (300 MHz, $CDCl_3$) δ_{ppm}:* -152.5 ppm (2F, d, J_{1-2} = 18.4 Hz, **F^1, ortho**); -158.0 ppm (1F, t, J_{1-2} = 20.7 Hz, **F^3, para**); -162.3 ppm (2F, t, J_{1-2} = 20.5 Hz, **F^2, meta**). *^{13}C NMR (400 MHz, $CDCl_3$) δ_{ppm}:* 63.0 ppm (**CH_2**-O), 57.2 ppm (**CH_2**-N), 45.8 ppm (N-**$(CH_3)_2$**) and 44.7 ppm (**backbone quaternary C**). Solvent peaks: 77.04 ppm (t, $CDCl_3$). *IR ν* / cm^{-1}*:* 2972 - 2651 cm^{-1} (w, C-H stretch); 2821 cm^{-1} (w, asym. C-H stretch (N-CH_3)); 2770 cm^{-1} (w, sym. C-H stretch (N-CH_3)); 1722 cm^{-1} (s, C=O stretch); 1522 cm^{-1} (w, C-F stretch); 1456 cm^{-1} (m, C-H bend (CH_2)). 1172 cm^{-1} (s, C-O stretch); 1144 cm^{-1} (s, C-N stretch), 1063 cm^{-1} (w, S-(C=S)-S stretch), 915 cm^{-1} (w, C_5F_6 associated vibration).

Functionalisation of PFP-pDMAEMA$_n$ with DBCO-NH$_2$ and Thiocarbonate Reduction

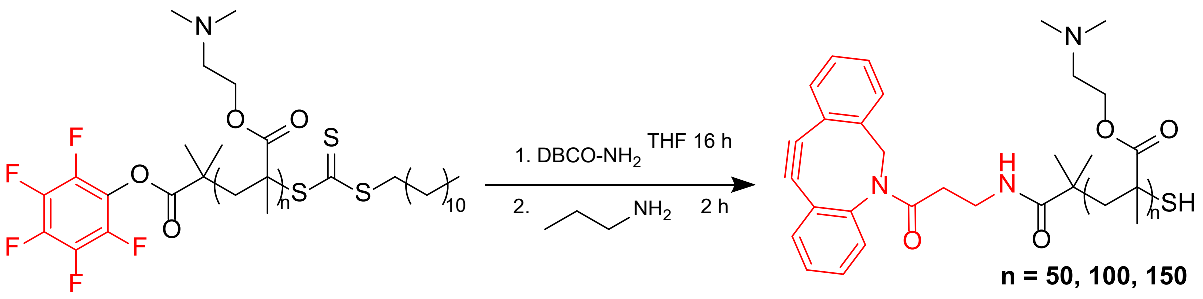

Figure S2. *Functionalisation of PFP-pDMAEMA$_n$ with DBCO-NH$_2$.*

PFP-pDMAEMA$_n$ (0.20 g, 1 Eq), and dibenzocyclooctyne-amine (DBCO-NH$_2$; 2 Eq) were stirred in THF (3 mL) for 16 h. Subsequent addition of propyl amine (1.5 eq) for 2 h was used to ensure complete reduction of the thiocarbonate moiety to a thiol group. The polymer was reprecipitated into cold hexane from THF three times, yielding a white polymer product. The resulting product was dried under vacuum and DMF SEC analysis was completed, Table S2. An aliquot was also taken for NMR analysis in CDCl$_3$.

Table S2. *SEC results of DBCO-pDMAEMA$_n$.*

Polymer	$M_{n,th}$ [a] (g.mol^{-1})	$M_{n,exp}$ [b] (g.mol^{-1})	$M_{W,exp}$ [b] (g.mol^{-1})	$Đ$[b]
DBCO-pDMAEMA$_{50}$	7531	12500	13900	1.11
DBCO-pDMAEMA$_{100}$	15313	14600	16400	1.12
DBCO-pDMAEMA$_{150}$	22309	17200	19300	1.12

[a] Determined assuming 100% successful DBCO functionalisation.
[b] Determined by SEC in DMF against PMMA standards.

DBCO-pDMAEMA$_{50}$: ^{1}H NMR (300 MHz, CDCl$_3$) δ_{ppm}: 7.73-7.31 ppm (8H, br. m, **aromatic DBCO CH**)) 5.19-5.11 ppm (DBCO **CH$_2$**-N), 4.06 ppm (100H, s, **CH$_2$**-O); 2.56 ppm (100H, br. s, **CH$_2$**-N,); 2.28 ppm (300H, s, N-**(CH$_3$)$_2$**); 2.12-1.75 ppm (100H, multiple br. s, **backbone CH$_2$**); 1.15-0.772 ppm (150H, **backbone CH$_3$**). Solvent peaks: 3.75 ppm and 1.83 ppm (m, THF) solvent peak and 7.26 ppm (s, CDCl$_3$). *^{19}F NMR (300 MHz, CDCl$_3$) δ_{ppm}:* Peaks have

disappeared. *^{13}C NMR (400 MHz, $CDCl_3$) δ_{ppm}:* 63.0 ppm (**CH_2**-O), 57.1 ppm (**CH_2**-N), 45.8 ppm (N-**$(CH_3)_2$**) and 44.8 ppm (**backbone quaternary C**). Solvent peaks: 77.04 ppm (t, $CDCl_3$). *IR v /* cm^{-1}*:* 3043 - 2693 cm^{-1} (w, multiple C-H stretch); 2819 cm^{-1} (w, asym. C-H stretch (N-CH_3)); 2776 cm^{-1} (w, sym. C-H stretch (N-CH_3)); 1719 cm^{-1} (s, C=O stretch); 1676-1620 cm^{-1} (w, aromatic C-H stretch overtone), 1452 cm^{-1} (m, C-H bend (CH_2)), 1173 cm^{-1} (s, C-O stretch); 1143 cm^{-1} (s, C-N stretch).

DBCO-pDMAEMA$_{100}$: ^{1}H NMR (300 MHz, $CDCl_3$) δ_{ppm}: 7.72-7.29 ppm (8H, br. m, **aromatic DBCO CH)**) 5.20-5.13 ppm (DBCO **CH_2**-N), 4.06 ppm (200H, s, **CH_2**-O); 2.56 ppm (200H, br. s, **CH_2**-N,); 2.28 ppm (600H, s, N-**$(CH_3)_2$**); 2.11-1.75 ppm (200H, multiple br. s, **backbone CH_2**); 1.17-0.771 ppm (300H, **backbone CH_3**). Solvent peaks: 3.75 ppm and 1.83 ppm (m, THF) solvent peak and 7.26 ppm (s, $CDCl_3$). *^{19}F NMR (300 MHz, $CDCl_3$) δ_{ppm}:* Peaks have disappeared. *^{13}C NMR (400 MHz, $CDCl_3$) δ_{ppm}:* 63.0 ppm (**CH_2**-O), 57.3 ppm (**CH_2**-N), 45.9 ppm (N-**$(CH_3)_2$**) and 44.9 ppm (**backbone quaternary C**). Solvent peaks: 77.04 ppm (t, $CDCl_3$). *IR v /* cm^{-1}*:* 3033 - 2693 cm^{-1} (w, multiple C-H stretch); 2819 cm^{-1} (w, asym. C-H stretch (N-CH_3)); 2776 cm^{-1} (w, sym. C-H stretch (N-CH_3)); 1720 cm^{-1} (s, C=O stretch); 1672-1618 cm^{-1} (w, aromatic C-H stretch overtone), 1455 cm^{-1} (m, C-H bend (CH_2)), 1171 cm^{-1} (s, C-O stretch); 1143 cm^{-1} (s, C-N stretch).

DBCO-pDMAEMA$_{150}$: ^{1}H NMR (300 MHz, $CDCl_3$) δ_{ppm}: 7.74-7.29 ppm (8H, br. m, **aromatic DBCO CH)**) 5.21-5.12 ppm (DBCO **CH_2**-N), 4.06 ppm (300H, s, **CH_2**-O); 2.56 ppm (300H, br. s, **CH_2**-N,); 2.28 ppm (900H, s, N-**$(CH_3)_2$**); 2.08-1.74 ppm (300H, multiple br. s, **backbone CH_2**); 1.12-0.779 ppm (450H, **backbone CH_3**). Solvent peaks: 3.75 ppm and 1.83 ppm (m, THF) solvent peak and 7.26 ppm (s, $CDCl_3$). *^{19}F NMR (300 MHz, $CDCl_3$) δ_{ppm}:* Peaks have disappeared. *^{13}C NMR (400 MHz, $CDCl_3$) δ_{ppm}:* 63.0 ppm (**CH_2**-O), 57.3 ppm (**CH_2**-N), 45.9 ppm (N-**$(CH_3)_2$**) and 44.9 ppm (**backbone quaternary C**). Solvent peaks: 77.04 ppm (t, $CDCl_3$). *IR v /* cm^{-1}*:* 3040 - 2693 cm^{-1} (w, C-H stretch); 2821 cm^{-1} (w, asym. C-H stretch (N-

CH_3)); 2770 cm^{-1} (w, sym. C-H stretch (N-CH_3)); 1722 cm^{-1} (s, C=O stretch); 1676-1626 cm^{-1} (w, aromatic C-H stretch overtone), 1454 cm^{-1} (m, C-H bend (CH_2)), 1172 cm^{-1} (s, C-O stretch); 1144 cm^{-1} (s, C-N stretch).

Fluorophore labelled DBCO-pDMAEMA$_n$

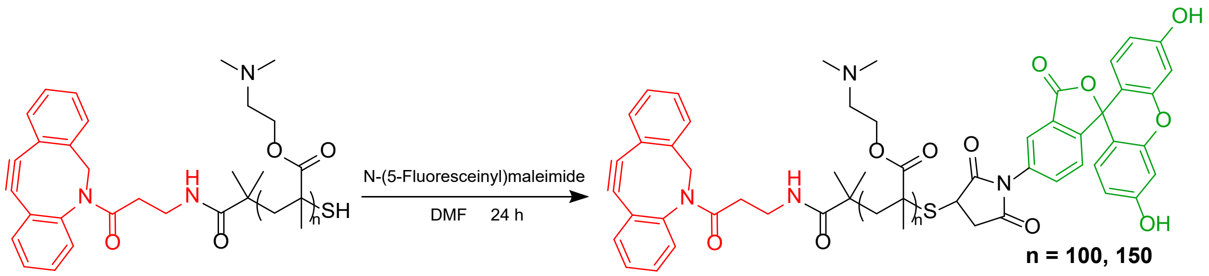

Figure S3. *Labelling DBCO-pDMAEMA$_n$ with fluorescein (DBCO-pDMAEMA$_n$-Fl).*

DBCO-pDMAEMA$_n$ (0.10 g, 1 Eq), and *N*-(5-fluoresceinyl)maleimide (1.5 Eq) were dissolved in DMF (2 mL), degassed and left to stir for 24 h. The yellow mixture was reprecipitated into cold hexane from THF three times, yielding a yellow fluorescent polymer product. DMF SEC analysis was completed with the UV-Vis detector set at 494 nm to demonstrate size separation and absorbance overlap, Table S3 and Fig. S12. Polymer: dye ratios were calculated using UV-Vis.

Table S3. *SEC results of DBCO-pDMAEMA$_n$-Fl*

Polymer	$M_{n,th}$[a] (g.mol^{-1})	$M_{n,exp}$[b] (g.mol^{-1})	$M_{W,exp}$[b] (g.mol^{-1})	$Đ$[b]	*Polymer:Dye*[c]
DBCO-pDMAEMA$_{100}$-Fl	15740	21800	29700	1.36	0.96
DBCO-pDMAEMA$_{150}$-Fl	22736	23600	32600	1.38	1.03

[a] Determined assuming 100% successful DBCO functionalisation.
[b] Determined by SEC in DMF against PMMA standards.
[c] Calculated using UV-Vis spectroscopy and $M_{n,exp}$ (Fluorescein ε = 73,000 cm^{-1}M^{-1}).

1.5 General Protocol for Metabolic Labelling of Cell Lines

A549 were seeded in Falcon 100 x 20 mm TC-treated cell culture dishes at a density of 25k cell.mL^{-1} (250k cells per well) in Ham's F-12K (Kaighn's) Medium (F-12K) supplemented with 10% FBS, 1% PSA (complete media) and Ac_4ManNAz (40 µM) in a humidified atmosphere of 95% air and 5% CO_2 at 37 °C for 72 h. SW480 were seeded in Falcon 100 x 20 mm TC-treated cell culture dishes at a density of 50k cell.mL^{-1} (500k cells per well) in Advanced Dulbecco's Modified Eagle's Medium supplemented with 10% FBS, 1% PSA and Ac_4ManNAz (40 µM) for 72 h in a humidified atmosphere of 95% air and 5% CO_2 at 37 °C. MCF-7 were seeded in Falcon 100 x 20 mm TC-treated cell culture dishes at a density of 100k cell.mL^{-1} (1 million cells per well) in Dulbecco's Modified Eagle's Medium - high glucose supplemented with 10% FBS, 1% PSA and Ac_4ManNAz (40 µM) in a humidified atmosphere of 95% air and 5% CO_2 at 37 °C for 72 h.

1.6 DBCO-pDMAEMA$_n$ and Doxorubicin Cytotoxicity

Resazurin Assay. Ac_4ManNAz treated and untreated A549 and SW480 cells (40 µM, 72 h) were seeded in a 96 well plate at a density of 25k cell.mL^{-1} (5k cells per well) and MCF-7 cells at a density 75k cell.mL^{-1} (15k cells per well), in the presence and absence of Ac_4ManNAz (40 µM), and were allowed to attach for 24 h. Following DPBS washes (x3), all cells were incubated with complete media supplemented with varying concentrations of DBCO-pDMAEMA$_n$ (0 – 250 µg.ml^{-1}) or doxorubicin (0 – 250 µg.ml^{-1}) for 2.5 h. Following three washes with DPBS, cell viability was determined by adding alamarBlue® reagent (10% v/v in cell media) to cells 24 h post-treatment. Cells were also incubated with doxorubicin (0 – 250 µg.ml^{-1}) for 24 h and 48h (at half-cell density), immediately washed with DPBS and alamarBlue® reagent was added. Absorbance measurements were obtained at 570 nm and 600 nm using a BioTek Synergy HT microplate reader to monitor the reduction of resazurin to

resorufin by viable cells. Each sample was incubated for 4 h (or until resazurin reduction reached max 70%) with alamarBlue® solution at 37 ºC and 5% CO_2 with readings obtained every 30 min / 1 h. Negative control cells untreated and treated with $Ac_4ManNAz$ (40 µM) were treated with alamarBlue® solution to provide a maximum resazurin reduction value of viable cells. A solution containing media alone and alamarBlue® solution was also measured for subtraction of percentage resazurin reduction values contributed from phenol red in media. Percentage cell viability was reported relative to either cells grown solely in cell culture media alone or $Ac_4ManNAz$. Five biological repeats were completed.

Calculating Percentage Resazurin Reduction:

$$\frac{(\varepsilon_{OX})\lambda_2 A\lambda_1 - (\varepsilon_{OX})\lambda_1 A\lambda_2}{(\varepsilon_{RED})\lambda_1 A'\lambda_2 - (\varepsilon_{RED})\lambda_2 A'\lambda_1} \text{x } 100$$

$\lambda_1 = 570\ nm, \lambda_2 = 600\ nm$

$A\lambda_1 and\ A\lambda_2 = Absorbance\ of\ test\ sample, control\ or\ media\ alone\ at\ 570\ nm\ and\ 600\ nm$

$A'\lambda_1 and\ A'\lambda_2 = Absorbance\ of\ media\ alone\ at\ 570\ nm\ and\ 600\ nm$

$(\varepsilon_{OX})\ and\ (\varepsilon_{RED}) = Molar\ extinction\ coefficient\ of\ resazurin\ at\ respective\ wavelengths$

Calculating Percentage Viability Relative to Control Cells:

$$\frac{\%\ Resazurin\ Reduction\ of\ Test\ Sample - \%\ Resazurin\ Reduction\ of\ Media\ Alone}{\%\ Resazurin\ Reduction\ of\ Cell\ Control - \%\ Resazurin\ Reduction\ of\ Media\ alone} \text{x } 100$$

LDH Assay. $Ac_4ManNAz$ treated and untreated A549 and SW480 cells (40 µM, 72 h) were seeded in a 96 well plate at a density of 50k cell.mL^{-1} (10k cells per well) and MCF-7 cells at a density 75k cell.mL^{-1} (15k cells per well), in the presence and absence of $Ac_4ManNAz$ (40 µM), and were allowed to attach for 24 h. Following this, cells were washed with DPBS (x3) and incubated with complete media supplemented with varying concentrations of DBCO-$pDMAEMA_n$ (0 − 250 µg.ml^{-1}, 100 µL) for 2.5 h. Lysis controls were also prepared for $Ac_4ManNAz$ treated and untreated cells by adding lysis solution provided by the Thermo

Scientific™ Pierce™ LDH Cytotoxicity Assay Kit to cells. Briefly, $Ac_4ManNAz$ treated and untreated cells were washed with DPBS (x3) and incubated with 100 µL of complete media containing lysis solution (10 µL prepared in ultrapure H_2O) for 45 min. Ultrapure water (10 µL) was also added to polymer treated and untreated cells, following the 2.5 h incubation, to maintain the same conditions as lysis controls. The supernatants of polymer treated cells, the lysis controls and polymer untreated cell controls were transferred to a separate 96 well plate (50 µL) and the LDH assay was completed immediately afterwards. LDH reaction mixture (50 µL) was added to the sample and control supernatants (50 µL), incubated at RT for 30 min and stop solution was subsequently added (50 µL), provided by the Thermo Scientific™ Pierce™ LDH Cytotoxicity Assay Kit. Absorbance readings were recorded at 490 nm and 680 nm using a BioTek Synergy HT microplate reader to monitor active LDH release from plasma membrane damage and as a background subtraction measurement, respectively. Three biological repeats were completed.

LDH Activity and % LDH Release/Cytotoxicity Calculations:

$$LDH\ Activity = A_{490\ nm} - A_{680\ nm}$$

$$\%\ LDH\ Release/Cytotoxicity = \frac{Polymer\ Treated\ LDH\ Activity - Cell\ Alone\ LDH\ Activity}{Lysate\ Control\ LDH\ Activity - Cell\ Alone\ LDH\ Activity} \text{ x } 100$$

Live/ Dead. $Ac_4ManNAz$ treated and untreated A549 and SW480 cells (40 µM, 72 h) were seeded in a 96 well plate at a density of 50k cell.mL^{-1} (10k cells per well) and MCF-7 cells at a density 75k cell.mL^{-1} (15k cells per well), in the presence and absence of $Ac_4ManNAz$ (40 µM), and were allowed to attach for 24 h. Following this, cells were washed with DPBS (x3) and incubated with complete media supplemented with varying concentrations of DBCO-pDMAEMA$_{100/150}$ (2 – 250 µg.ml^{-1}, 100 µL) for 2.5 h. The polymer solutions were removed, cells washed with DPBS (x3) and incubated in complete media for 24 h in a humidified atmosphere of 95% air and 5% CO_2 at 37 °C for 96 h. Following removal of media and DPBS

washes (x3), cells were stained with ethidium iodide (2 μM) and calcein (2 μM) in DPBS (100 μL) for 40 mins at RT. $Ac_4ManNAz$ untreated and treated cells were also stained as control viable cell samples. Cells were imaged on an Olympus CX41 microscope equipped with a UIS-2 20x/0.45/∞/0−2/FN22 lens using a phase contrast channel and with blue (calcein) and green (ethidium) excitation lasers. Cells were counted using ImageJ. Values were reported as percentage live cells relative to the total number of cells (four biological repeats were completed).

1.7 DBCO-pDMAEMA$_n$ Haemolysis

Ovine blood (TCS Biosciences, UK) was centrifuged at 2000 rpm for 5 min and re-suspended in DPBS to generate a 30% haematocrit solution. The ovine blood solution (100 μL) was transferred to a 96 well plate and incubated with DBCO-pDMAEMA$_{100/150}$ (100 μL, 3h). Blood incubated with either lysis buffer (0.32 M sucrose, 5 mM magnesium chloride, 1% triton X-100, 10 mM Tris HCl pH 7.8) or DPBS, for 3 h, was completed to provide a positive and negative control, respectively. Wells filled with DPBS alone (200 μL) was provided as a vehicle control for background subtraction. After three hours, the cells were centrifuged at 3700 rpm for 5 min and the alkaline haematin D-575 (AHD575) assay was conducted. Briefly, 8 μL of the supernatant from each well was added to 100 μL of AHD solution (2.5 w/v% triton-X100 and 0.1 M NaOH in water) and absorbance measurements were recorded at 580 nm. Values were reported as %haemolysis (four biological repeats were completed).

$$\%haemolysis = \frac{Sample\ Absorbance_{580} - Vehicle\ Control\ Absorbance_{580}}{Positive\ Control\ Absorbance_{580} - Vehicle\ Control\ Absorbance_{580}} \text{ x } 100$$

1.7 Additional Mechanistic Studies

Annexin/ PI. $Ac_4ManNAz$ treated and untreated A549 and SW480 cells (40 μM, 72 h) were seeded in a 96 well plate at a density of 50k cell.mL^{-1} (10k cells per well) and MCF-7 cells at

a density 75k cell.mL^{-1} (15k cells per well), in the presence and absence of Ac4ManNAz (40 μM), and allowed to attach for 24 h. Following this, all cells were incubated with complete media supplemented with DBCO-pDMAEMA$_{100/150}$ (EC$_{90}$ concentration determined by cell viability data, 2.5 h), washed with DPBS and treated with FITC Annexin V/Dead Cell Apoptosis Kit (Invitrogen). Briefly, DPBS was replaced with a solution consisting of Annexin-Binding buffer (75 μL), FITC Annexin (25 μL) and Propidium Iodide (PI) (2 μL of 100 μg.mL^{-1}) and allowed to incubate at RT for 15 min. Cells were subsequently washed with Annexin-Binding Buffer (x3) and fresh buffer was added. Cells were imaged on an Olympus CX41 microscope equipped with a UIS-2 20x/0.45/∞/0−2/FN22 lens using a phase contrast channel and with blue (FITC Annexin) and green (PI) excitation lasers.

Caspase-3/7 Kinetics Apoptosis Assay. Ac$_4$ManNAz treated and untreated A549 and SW480 cells (40 μM, 72 h) were seeded in a 96 well plate at a density of 50k cell.mL^{-1} (10k cells per well) in the presence and absence of Ac$_4$ManNAz (40 μM) and allowed to attach for 24 h. Following this, all cells were washed with DPBS (x3) and incubated with complete media supplemented with CellEvent Caspase-3/7 Detection Reagent (5 μM) and DBCO-pDMAEMA$_{100/150}$ (0 – 125 μg.ml^{-1}). Images were taken at 0.5, 1, 2.5 and 4 h time intervals on an Olympus CX41 microscope using a phase contrast channel and a blue excitation laser. Ac$_4$ManNAz untreated and treated cells were also stained as 'negative' control cells. Cells were counted using ImageJ and values were reported as percentage caspase positive cells relative to the total number of cells. Four biological repeats were completed.

Polymer Degrafting Post-Treatment Fluorescence. Ac$_4$ManNAz treated and untreated A549 and SW480 cells (40 μM, 72 h) were seeded in a black opaque 96 well plate at a density of 50k cell.mL^{-1} (10k cells per well) in the presence and absence of Ac$_4$ManNAz (40 μM) and allowed to attach for 24 h. Following this, cells were washed with DPBS (x3) and incubated with complete media supplemented with varying concentrations of DBCO-pDMAEMA$_{100/150}$-Fl (0

– 250 μg.ml^{-1}, 100 μL) for 2.5 h. The polymer solutions were removed, cells washed with DPBS (x3) and replaced with complete media. Fluorescence measurements were recorded using a BioTek Synergy HT microplate reader using a 488 nm excitation later and 528 filter immediately, 0.5 h, 1 h, 2 h and 4 h post-treatment. At each time point, supernatants were collected, replaced with fresh media and the fluorescence of both cells and supernatants were recorded. Four biological repeats were completed.

Polymer Grafting and Membrane Damage Confocal Microscopy. Ac_4ManNAz treated and untreated A549 and SW480 cells were seeded in the small compartment of CELLview™ Culture dishes (Greiner Bio-One) at a density of 25k cell.mL^{-1} (5k cells) in the presence and absence of Ac_4ManNAz (40 μM) and allowed to attach for 24 h. Cells were washed with DPBS (x3) and subsequently incubated with DBCO-pDMAEMA$_{100/150}$-Fl for 2.5 h. Polymer solutions were removed and replaced with Leibovitz's L-15 Medium with no phenol red (Gibco), following x3 DPBS washes. Confocal imaging was completed immediately afterwards using a Plan-Apochromat 63x/1.40 Oil DIC M27 objective lens, 488 nm excitation laser (2.0% power), filters set to 493 – 634 nm, a master gain of 750, digital gain of 1.00 and no digital offset. MBS: MBS 488, MBS_InVis: Plate and DBS1: Mirror beam splitters were selected and the pinhole size was kept at 1 AU (54 μm). Z-stacks were taken at 2.0 zoom every z = 0.41 μm until the whole cell was imaged. Images collected possessed dimensions of x: 792, y: 792, z: 10 – 12, channels: 2 (FITC and DIC), 16-bit. Averaging was kept to line 8 and pixel dwell 0.67 μs. Proceeding imaging, cells treated with DBCO-p(DMAEMA)$_{100/150}$ for 2.5 h were washed with DPBS (x3) and stained with CellMask™ Deep Red Plasma membrane Stain (25 μg.mL^{-1}, 100 μL) for 30 min at RT. Cells were washed, stained with NucBlue® Live Cell ReadyProbes® Reagent (5 min at RT) in L15 media with no phenol red and imaged. Control viable cells treated and untreated with Ac_4ManNAz but not treated with polymer solutions were also stained with the membrane and nuclear stain for comparison. Confocal imaging was completed using a

Plan-Apochromat 63x/1.40 Oil DIC M27 objective lens; Ch1: 405 nm, ChS1: 488 nm, Ch2: 633 nm excitation lasers all set to 2.0% power; filters set to Ch1: 410 – 483 nm, ChS1: 490 – 632 nm and Ch2: 638 – 747 nm; master gain of Ch1: 650, ChS1: 700 and Ch2: 700 – 1000; digital gain of 1.0 and no digital offset. MBS: MBS 488/561/633, MBS_InVis: MBS -405, DBS1: Mirror beam splitters were selected and the pinhole size was kept at 1 AU (42 μm). Z-stacks were taken of 2.0 zoom every z = 0.41 μm until the whole cell was imaged. Images collected possessed dimensions of x: 956, y: 956, z: 10 – 12, channels: 4 (DAPI, FITC, Deep Red, DIC), 16-bit. Averaging was kept to line 8 and pixel dwell 0.55 μs. Maximum intensity projection images and 3D reconstructions were produced using Zen 2.3 (blue edition).

1.8 General Protocol for Metabolic Glycan Labelling of Spheroids

A549 spheroids were metabolically labelled using two approaches: (1) spheroid formation in $Ac_4ManNAz$ (4 days) and (2) pre-formed spheroids (for 3 days) were subsequently treated with $Ac_4ManNAz$ (4 days). For method (1), A549 cells were seeded in an ultralow attachment U-bottom plate at a seeding density of (2k cells per well) in the presence and absence of $Ac_4ManNAz$ (40 μM). The plate was centrifuged at 2k RPM for 10 min and spheroids were allowed to grow for 96 h in a humidified atmosphere of 95% air and 5% CO_2 at 37 °C. For method (2), A549 cells were seeded in an ultralow attachment U-bottom plate at a seeding density of (1k cells per well) centrifuged at 2k RPM for 10 min and allowed to form for 72 h. Following removal of media, the preformed spheroids were treated with or without $Ac_4ManNAz$ (40 μM) supplemented media for 96 h. Spheroids made using both approaches were subsequently treated with polymer solutions, or doxorubicin, and subjected to cell viability and cytotoxicity assays (see section 1.9).

To confirm $Ac_4ManNAz$ recruitment with both methods, spheroids were stained with DBCO-Cy3 (50 μM, 2 h) and NucBlue® Live Cell ReadyProbes® Reagent (15 min). Confocal Z-stack

imaging was completed on a Zeiss LSM 880 microscope equipped with a EC Plan-Neofluar 10x/0.30 M27 objective lens, Ch1: 405 and Ch2: 514 nm excitation lasers (2.0% power), filters set to Ch1: 411 – 531 nm and Ch2: 538 – 680 nm, a master gain of Ch1: 750 and Ch2: 1000, digital gain of Ch1: 1.00 and Ch2: 2.00 and no digital offset. MBS: MBS 458/514, MBS_InVis: MBS -405 and DBS1: Mirror beam splitters were selected and the pinhole size was kept at 1 AU (43 μm). Images collected possessed dimensions of x: 2048, y: 2048, z: 33, channels: 3 (DAPI, Cy3, DIC), 16-bit. Z-stacks were taken every 5 μm, averaging was kept to line 8 and pixel dwell 0.52 μs. Spheroids untreated with $Ac_4ManNAz$ were also incubated with DBCO-Cy3, stained with NucBlue® Live Cell ReadyProbes® Reagent and imaged using the above conditions as a negative control. Maximum intensity projection images and 3D reconstructions were produced using Zen 2.3 (blue edition).

1.9 DBCO-pDMAEMA$_n$ Spheroid Cytotoxicity and Viability Assays

Spheroid Cytotoxicity Assay. A549 spheroids formed in the presence and absence of $Ac_4ManNAz$ (Method (1) from section 1.8) were treated with DBCO-pDMAEMA$_{100/150}$ at an appropriate set of concentrations (25, 62.5 and 250 μg.ml^{-1}) for 3 h. Polymer solutions were subsequently removed, spheroids were washed with DPBS (2x) and cell media was added (100 μL). CellTox^{TM} Green reagent (100 μL, 2X concentration) was subsequently added and cells were allowed to incubate at RT for 15 min. Spheroids were centrifuged at 2k RPM for 5 min to ensure homogeneity of assay solutions and to collect spheroids at the bottom of U bottom 96 well plates. Z-stack confocal imaging was completed using an EC Plan-Neofluar 10x/0.30 M27 objective lens, a 488 nm excitation laser (2.0% power), filter set to 493 – 634 nm, master gain of 550, digital gain of 1.00 and no digital offset. MBS: MBS 488, MBS_InVis: Plate, DBS1: Mirror beam splitters were selected and the pinhole size was kept at 1 AU. Images collected possessed dimensions of x: 2048, y: 2048, z: 30, channels: 2 (FITC and DIC), 16-bit.

Z-stacks were taken every 5 μm, averaging was kept to line 8 and pixel dwell 0.52 μs. $Ac_4ManNAz$ treated and untreated spheroids that were untreated with polymer solutions were analysed as above as negative controls. A lysis control was also prepared by adding lysis solution provided by the CellTox™ Green Cytotoxicity Assay (4 μL per 100 μL) for 30 min, as a positive control. Maximum intensity projection images and 3D reconstructions were produced using Zen 2.3 (blue edition).

Spheroid Viability Assay. A549 spheroids produced using both protocols from section 1.8 were incubated with DBCO-$pDMAEMA_n$ (31.3 – 500 $\mu g.mL^{-1}$, 100 μL) for 3 h in a humidified atmosphere of 95% air and 5% CO_2 at 37 °C. The polymer solutions were removed and spheroids were washed with DPBS (x3). Following replacement of media with fresh media (100 μL), an equal volume of CellTiter-Glo® was subsequently added and the plate was placed on a plate shaker for 10 min to ensure lysis. The plate was allowed to equilibrate for 25 min at RT and the solutions were transferred into an opaque white plate suitable for luminescence measurements collected using a BioTek Synergy HT microplate reader. A solution containing fresh media (100 μL) and CellTiter-Glo® was measured for background subtraction and percentage cell viability was calculated relative to polymer untreated $Ac_4ManNAz$ treated and untreated A549 spheroids. Five/six biological repeats were completed.

$$\%Spheroid\ Viability = \frac{Polmer\ treated\ Luminescence - Background\ Luminescence}{Viable\ Spheroid\ Luminescence - Background\ Luminescence} \text{ x } 100$$

1.10 Doxorubicin Spheroid Viability Assay

A549 spheroids produced using both protocols from section 1.8 were incubated with doxorubicin (31.3 – 500 $\mu g.mL^{-1}$, 100 μL) for 24 h. The solutions were removed and spheroids were washed with DPBS (x3). Following replacement of media with fresh media (100 μL), an equal volume of CellTiter-Glo® was subsequently added and the protocol was completed as described above (section 1.9).

2 Additional Results

2.1 Polymer Characterisation

2.1.1 PFP-pDMAEMA$_n$ and DBCO-pDMAEMA$_n$

PFP-pDMAEMA$_n$ and DBCO-pDMAEMA$_n$ polymers were fully characterised using ^{1}H and ^{19}F NMR spectroscopy (Fig. S4 – S6 and Fig. S7 – S9), IR spectroscopy (Fig. S13) and SEC (Fig. S14). SEC confirmed the synthesis of polymers of three different chain lengths, with sizes corroborating well with the theoretical M_n values (Figure 1, see manuscript). Any discrepancies between the experimental and theoretical M_n was attributed to the difference in molecular structure of the polymer products and the linear PMMA standards used in the GPC studies. Thiocarbonate conversion to thiol was evident by removal of ^{1}H NMR peaks associated to the connected $(CH_2)_{11}CH_3$ chain (~1.30 ppm (16H, t, H^{2-9}). Removal of a S-(C=S)-S stretch (1030 cm^{-1}, s) in IR spectra of DBCO-p(HEA)$_n$ provided further confirmation. Removal of PFP was established via the absence of peaks in ^{19}F NMR and C-F stretch (1520 cm^{-1} and 915 cm^{-1}) in IR spectra. Addition of a DBCO moiety was confirmed by the presence of aromatic DBCO CH peaks (7.80 ppm – 7.00 ppm) and peaks corresponding to DBCO CH_2-N (5.21-5.12 ppm) in ^{1}H NMR and DBCO aromatic overtone peaks (1676-1626 cm^{-1}) in IR spectra. The hydrophobic nature of DBCO resulted in increases to MW values obtained by SEC and dispersity readings due to non-specific hydrophobic secondary interaction with the SEC stationary phase, providing further confirmation of functionalisation. Finally, all integration values were in good agreement with expectations with minimal discrepancies due to polymer dispersity and residual solvent.

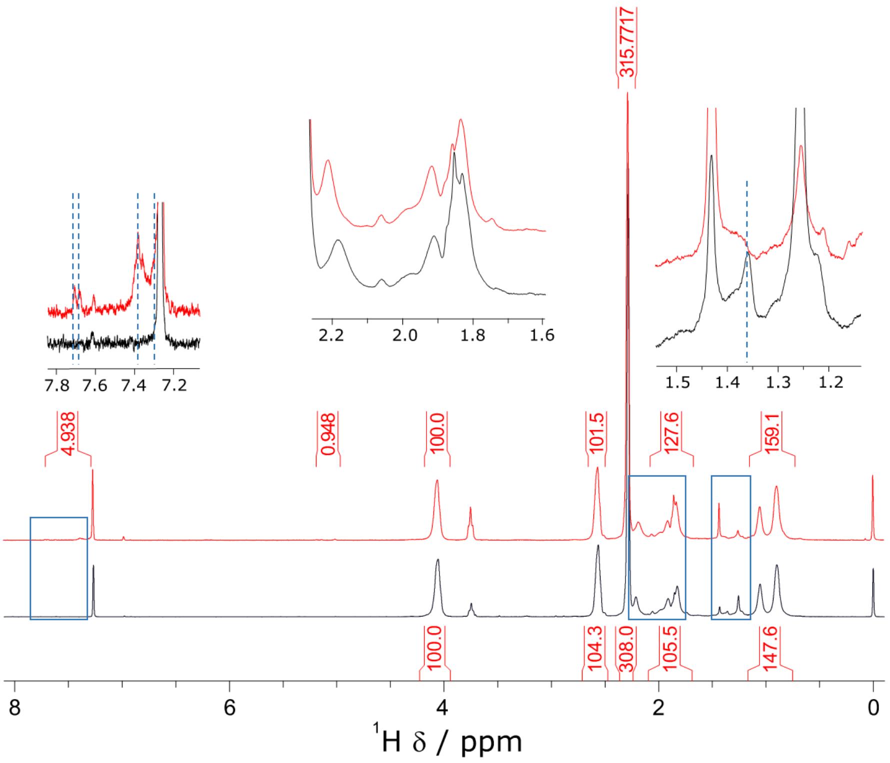

Figure S4. *^{1}H NMR of pDMAEMA$_{50}$ before (black) and after (red) functionalisation with DBCO. Peak alterations following functionalisation have been highlighted in blue.*

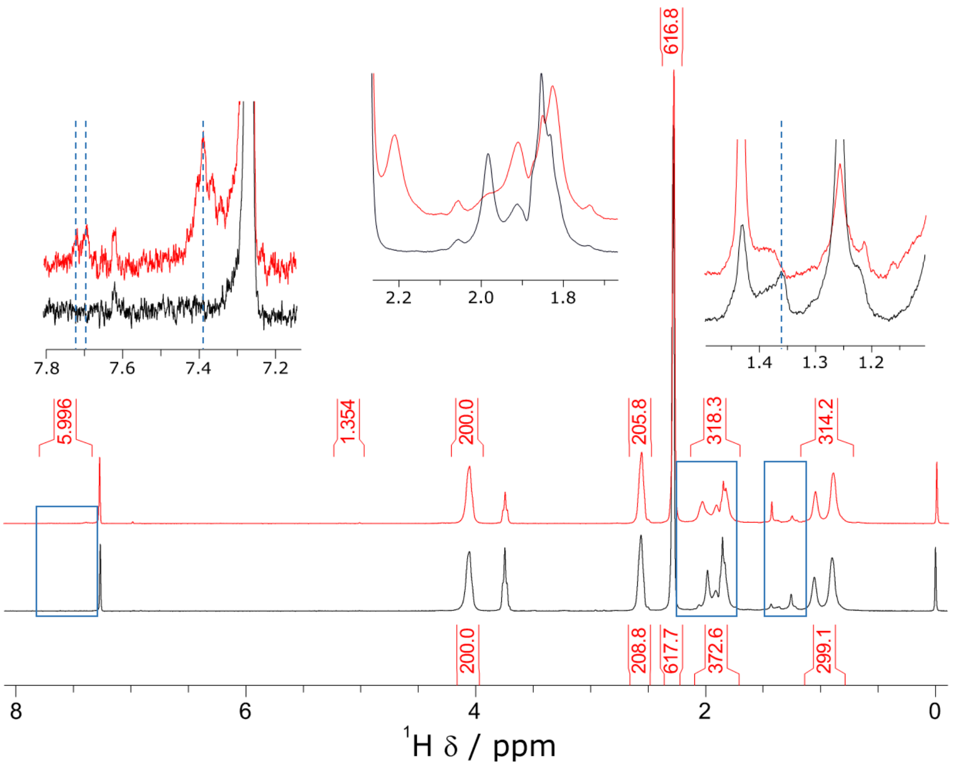

Figure S5. *^{1}H NMR of pDMAEMA$_{100}$ before (black) and after (red) functionalisation with DBCO. Peak alterations following functionalisation have been highlighted in blue.*

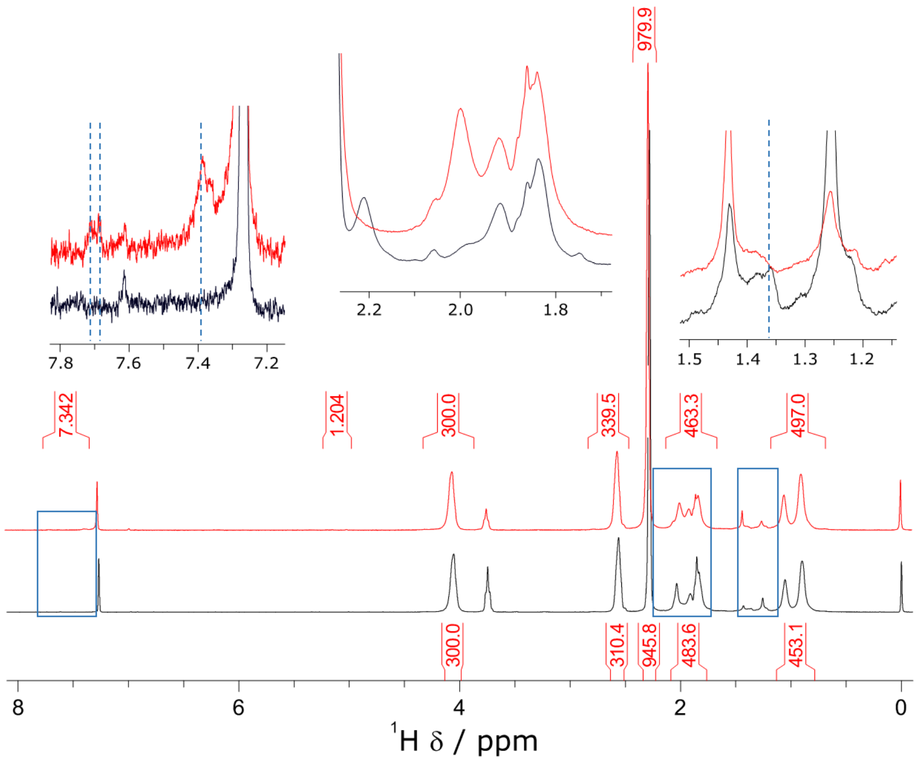

Figure S6. *^{1}H NMR of pDMAEMA$_{150}$ before (black) and after (red) functionalisation with DBCO. Peak alterations following functionalisation have been highlighted in blue.*

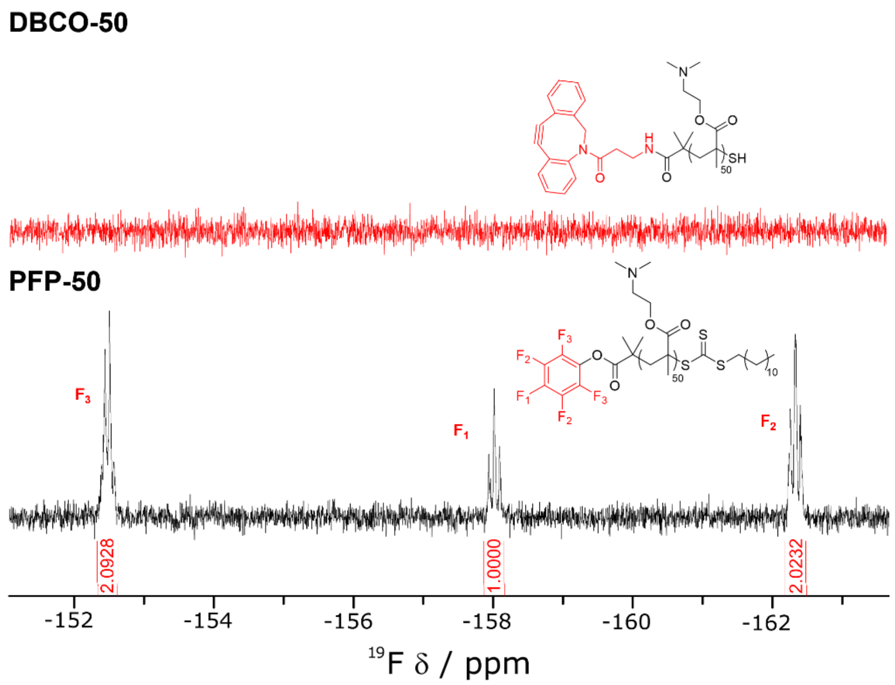

Figure S7. *^{19}F NMR of pDMAEMA$_{50}$ before (black) and after (red) functionalisation with DBCO. The peak representing the para fluorine of the PFP ester group (F$_1$), at ~158 ppm, has been calibrated to 1 to demonstrate correct integration values of all peaks. The disappearance of fluorine peaks confirms cleavage of the PFP ester and substitution with DBCO.*

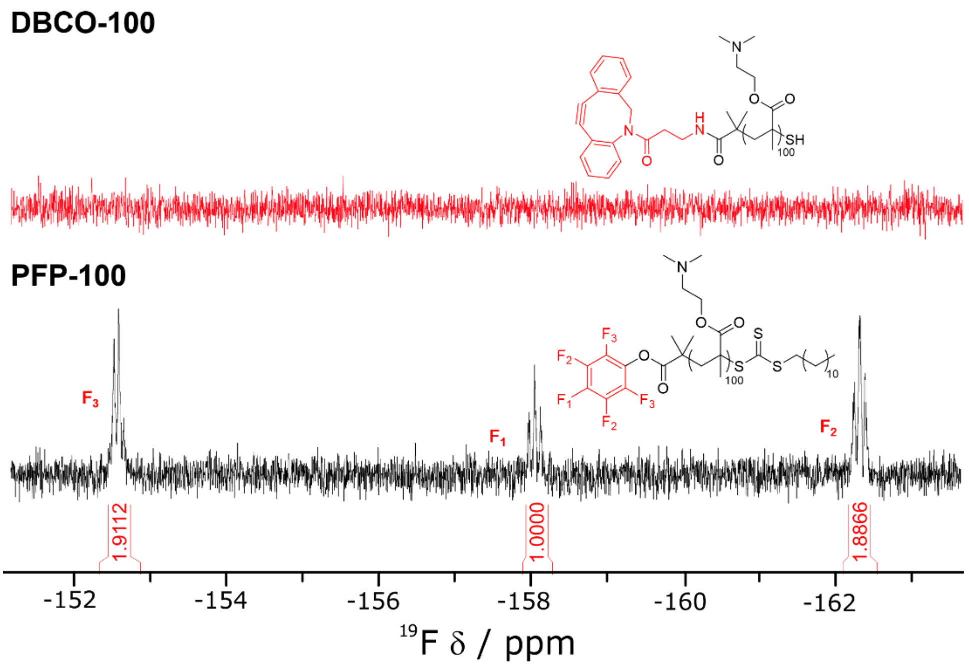

Figure S8. *^{19}F NMR of pDMAEMA100 before (black) and after (red) functionalisation with DBCO. The peak representing the para fluorine of the PFP ester group (F1), at ~158 ppm, has been calibrated to 1 to demonstrate correct integration values of all peaks. The disappearance of fluorine peaks confirms cleavage of the PFP ester and substitution with DBCO.*

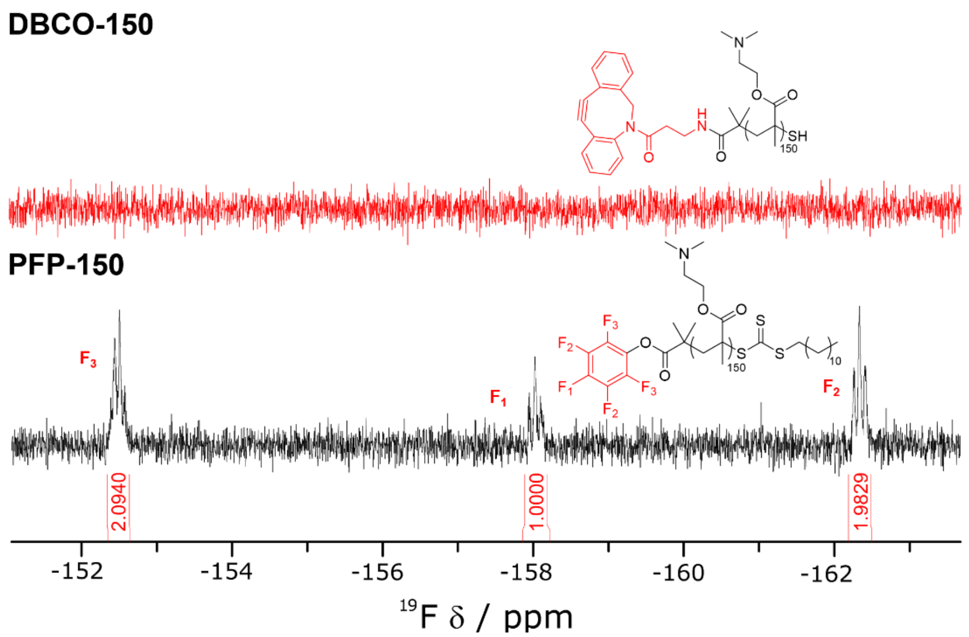

Figure S9. *^{19}F NMR of pDMAEMA150 before (black) and after (red) functionalisation with DBCO. The peak representing the para fluorine of the PFP ester group (F1), at ~158 ppm, has been calibrated to 1 to demonstrate correct integration values of all peaks. The disappearance of fluorine peaks confirms cleavage of the PFP ester and substitution with DBCO.*

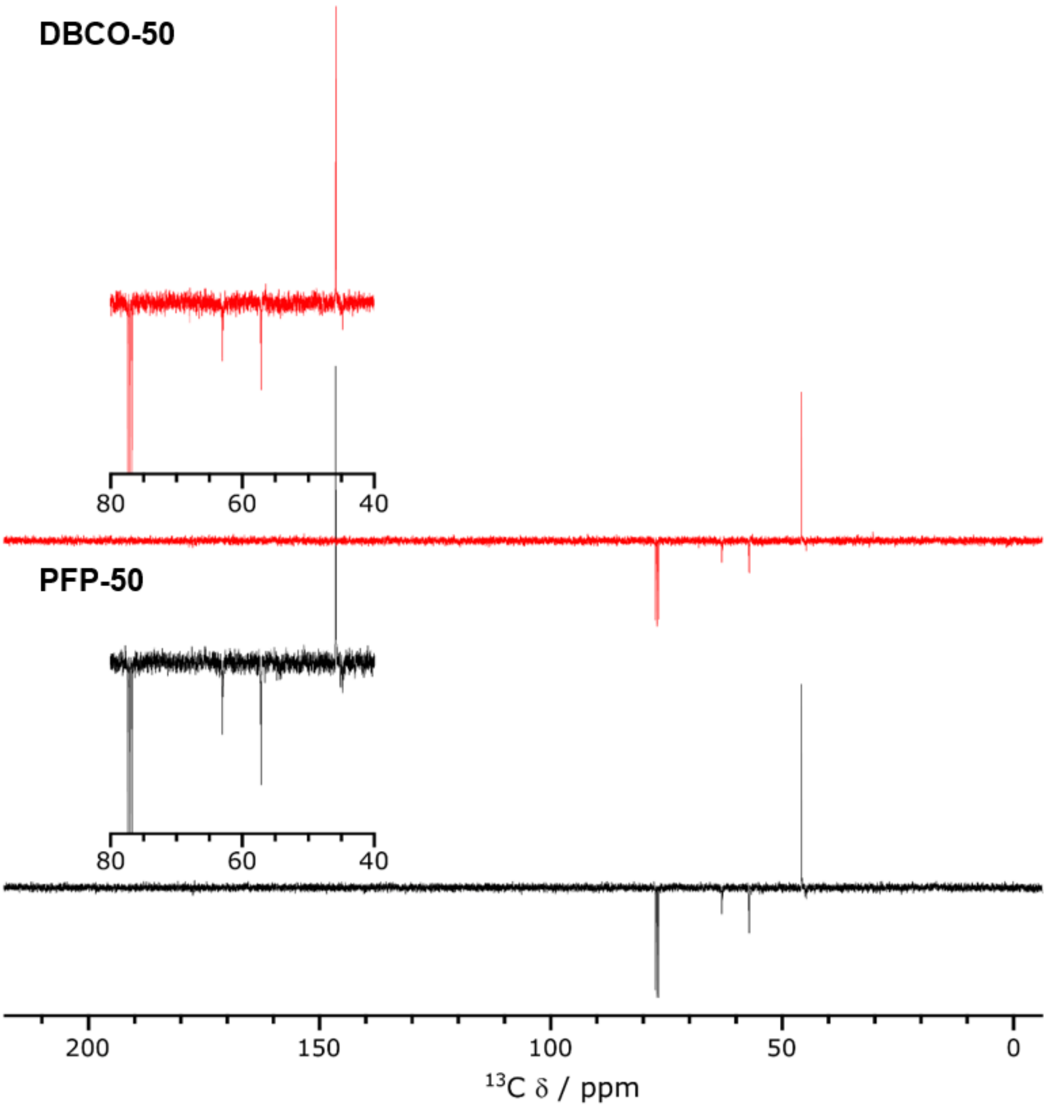

Figure S10. *^{13}C NMR of pDMAEMA$_{50}$ before (black) and after (red) functionalisation with DBCO.*

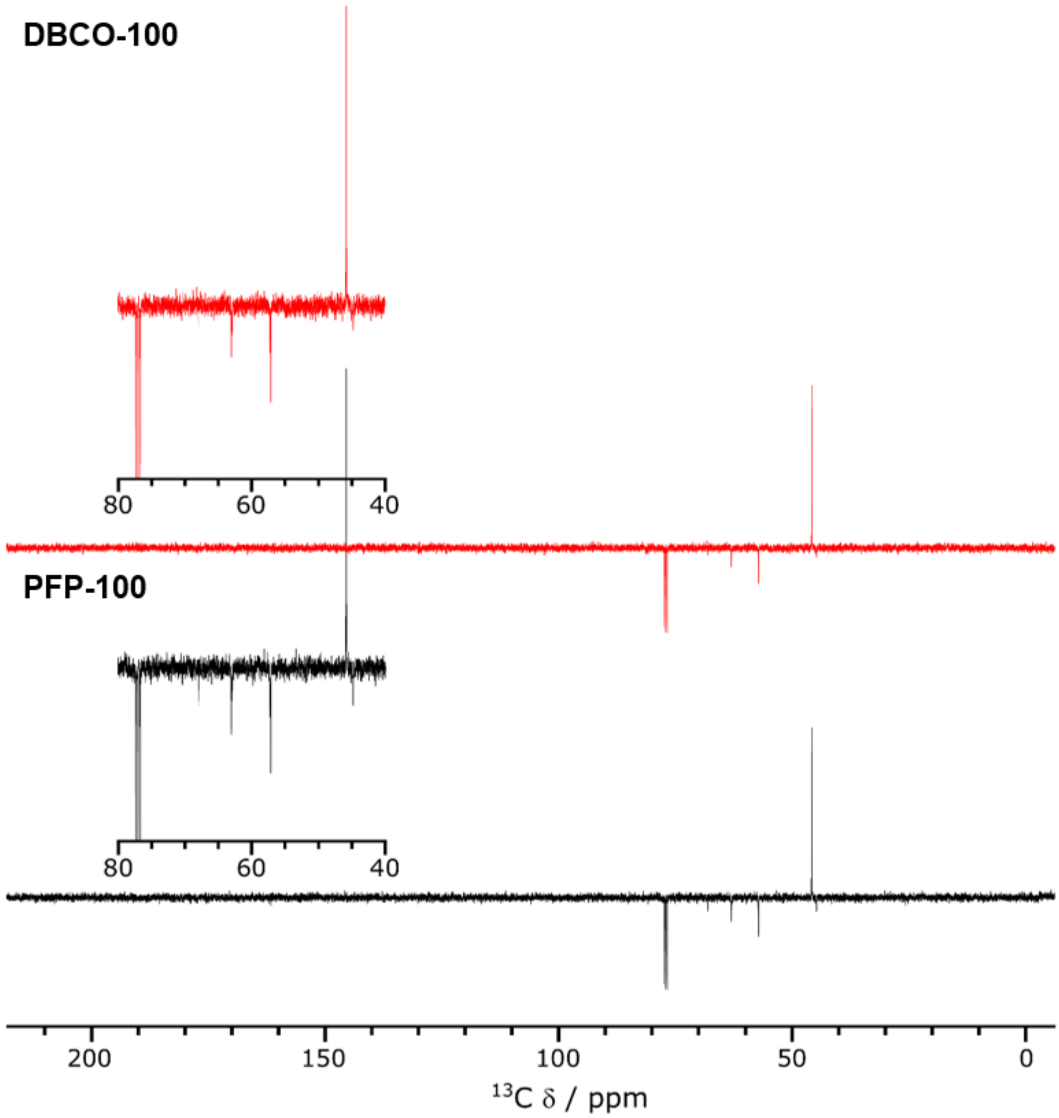

Figure S11. *^{13}C NMR of pDMAEMA$_{100}$ before (black) and after (red) functionalisation with DBCO.*

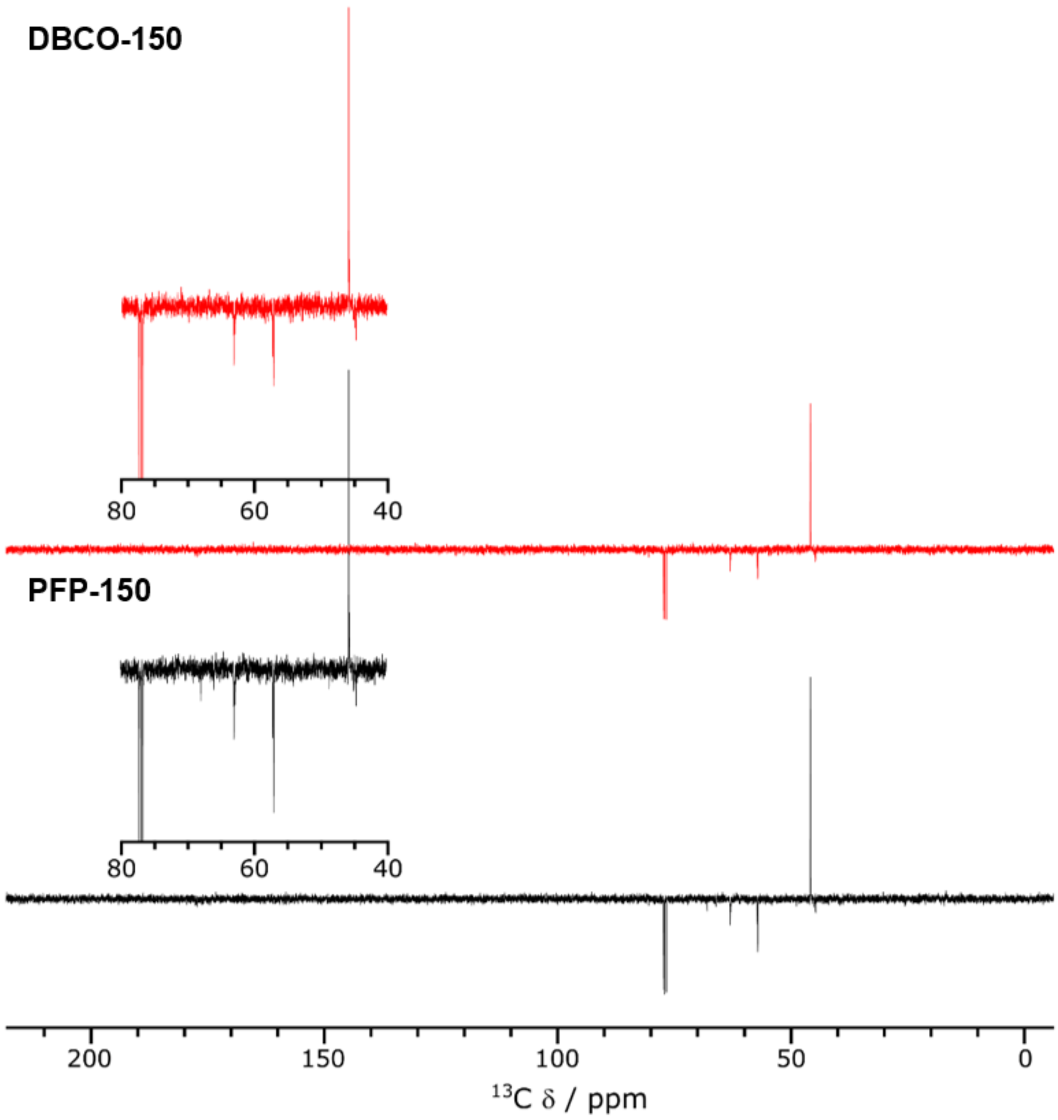

Figure S12. *^{13}C NMR of pDMAEMA$_{150}$ before (black) and after (red) functionalisation with DBCO.*

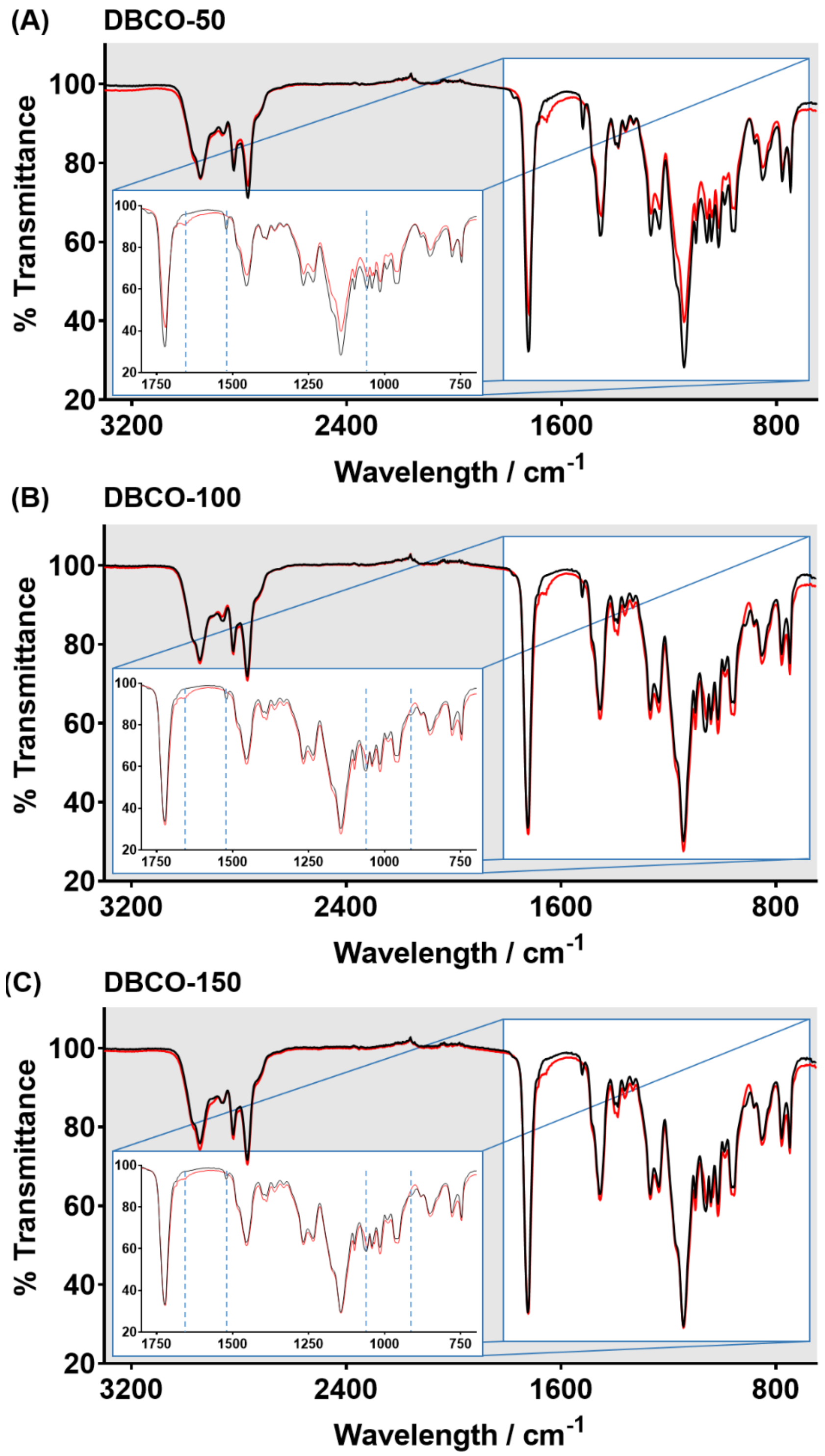

Figure S13. *IR spectrum of pDMAEMA$_n$ before (black) and after (red) functionalisation with DBCO. Peak alterations following functionalisation have been highlighted in blue. Confirmation of PFP cleavage and DBCO substitution was evident by the loss of peaks associated with C-F stretch (1522 cm^{-1} and 915 cm^{-1}) and the trithiocarbonate end group (1063 cm^{-1}). Addition of DBCO aromatic was also noted by the overtone peaks at 1676-1626 cm^{-1}.*

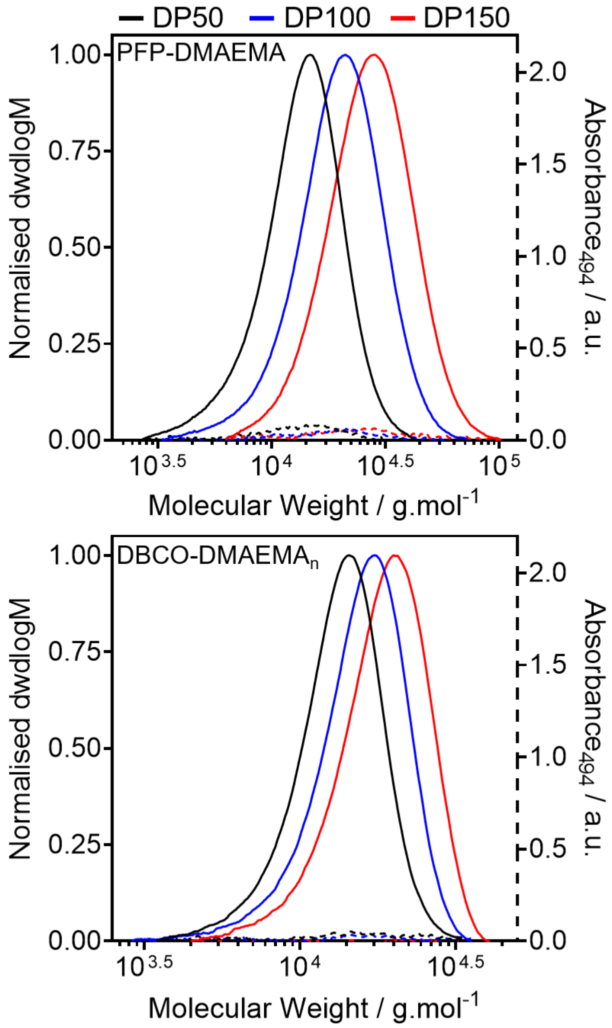

Figure S14. *SEC measurements of PFP-DMAEMA$_n$ and DBCO-DMAEMA$_n$ polymers were recorded using an RI detector (solid line), for size determination, and a UV detector (dashed line), to illustrate overlap with absorbance readings at 494 nm.*

2.1.2 DBCO-pDMAEMA$_n$-Fl

Previously, SEC results of PFP-pDMAEMA$_n$ and DBCO-pDMAEMA$_n$ demonstrated no absorbance at 494 nm (Fig. S14). Following fluorescein conjugation to the thiol terminated region of DBCO-pDMAEMA$_n$, SEC results demonstrated a dramatic increase in absorbance readings at 494 nm which also overlapped the size distribution profile (Fig. S12), providing confirmation of successful fluorescein attachment. Polymer:dye ratios (Table S3) were calculated using UV-Vis spectroscopy, Fig. S15.

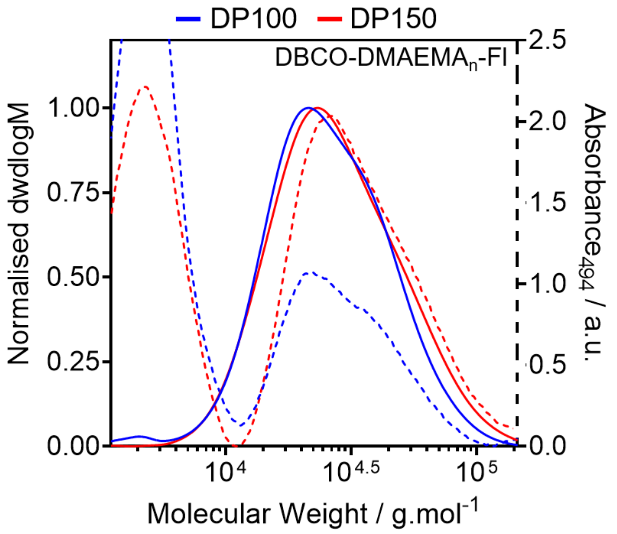

Figure S15. *SEC measurements of DBCO-DMAEMA$_n$-Fl polymers were recorded using an RI detector (solid line), for size determination, and a UV detector (dashed line), to illustrate overlap with absorbance readings at 494 nm.*

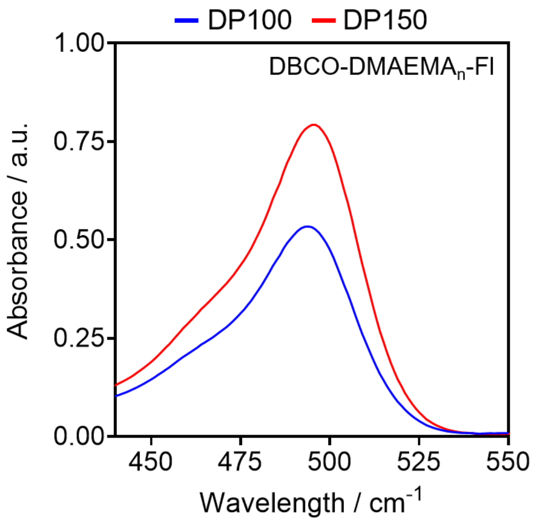

Figure S16. *UV-Vis spectrum of FL-100 (0.17 mg.mL^{-1}) and FL-150 (0.26 mg.mL^{-1}) used to calculate polymer:dye ratios.*

2.2 Resazurin Assays

2.2.1 DBCO-pDMAEMA$_n$ Resazurin Assays

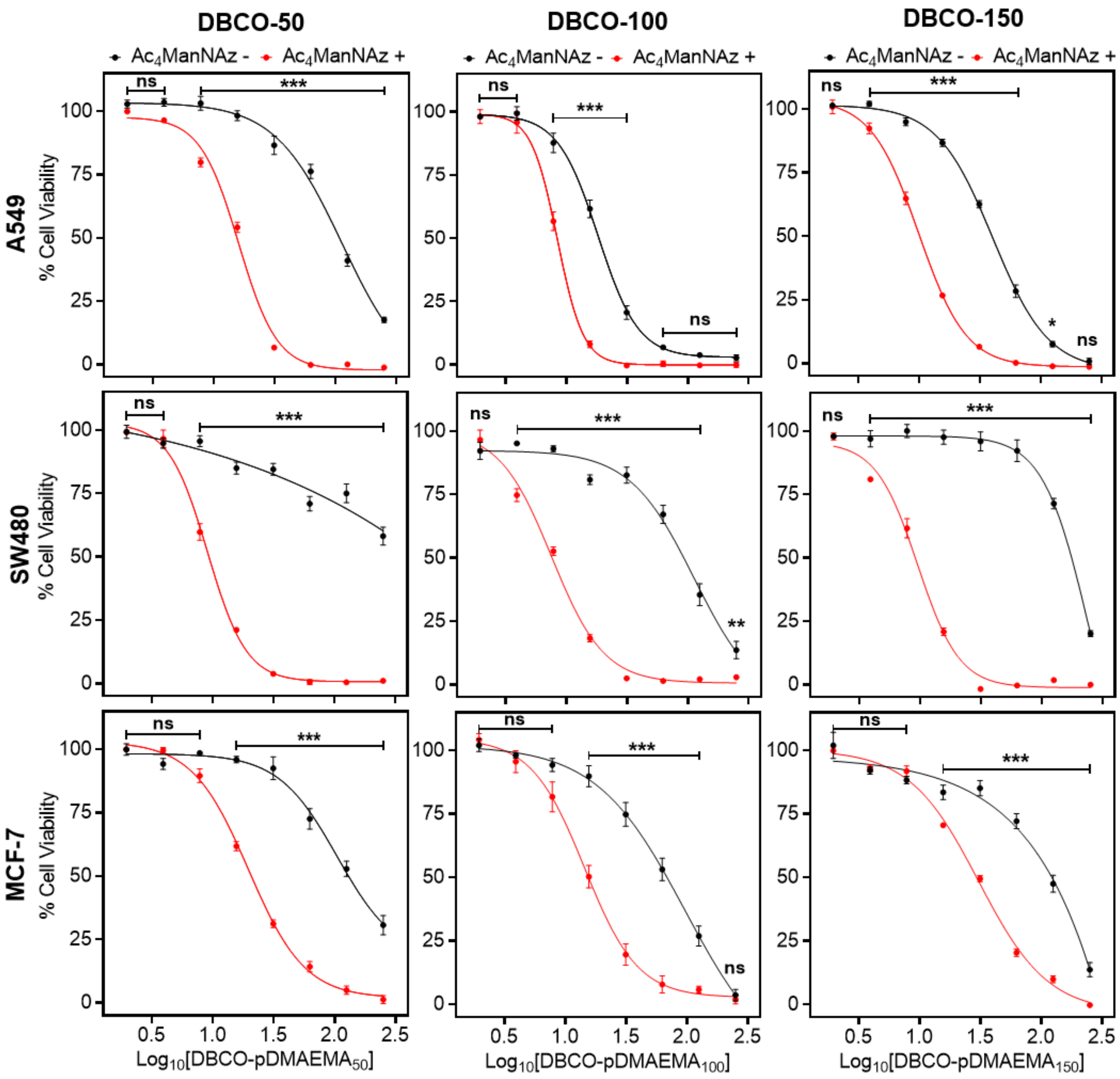

Figure S17. *Dose-response curves for the percentage cell viability of A549, SW480 and MCF-7 cells untreated* ***(black)*** *or treated* ***(red)*** *with Ac$_4$ManNAz (40 µM, 96 h) and treated with DBCO-pDMAEMA$_n$ (2 – 250 µg.ml^{-1}, 2.5 h) ranging in chain lengths (**DBCO-50**, **DBCO-100** and **DBCO-150**). Cell viability was determined by resazurin assays and data is presented as mean % cell viability relative to control cells ± SEM (n = 5) from five independent repeats. Statistical analysis was performed comparing % cell viability of Ac$_4$ManNAz untreated and treated cells at equal DBCO-pDMAEMA$_n$ concentrations. ns: $p \geq 0.05$, ** $p \leq 0.01$, *** $p \leq 0.001$.*

2.2.2 Percentage Change in Resazurin EC_{50} Values

Table S4. *Percentage change in EC_{50} values of DBCO-pDMAEMA$_n$ with an Ac_4ManNAz pretreatment. Values have been reported ± SEM (n = 5) from five independent repeats. EC_{50} values were determined from resazurin dose-response curves (Fig. S13) and have been reported in Table 1 (see article). The average % change and fold have been calculated across all polymer chain lengths.*

	A549		SW480		MCF-7	
	%	Fold	%	Fold	%	Fold
DBCO-50	84.5 ± 3.6	6.45 ± 0.29	> 100	> 100	85.4 ± 12.5	6.86 ± 0.69
DBCO-100	56.4 ± 5.6	2.29 ± 0.13	91.2 ± 13.7	11.3 ± 1.2	74.8 ± 12.0	3.97 ± 0.54
DBCO-150	74.6 ± 5.3	3.94 ± 0.21	94.0 ± 1.6	16.7 ± 0.8	75.7 ± 10.0	4.11 ± 0.35
Average	71.8 ± 8.2	4.23 ± 1.21	92.6 ± 1.1	14.0 ± 2.2	78.6 ± 3.4	4.98 ± 0.94

2.2.3 Doxorubicin Resazurin Assays

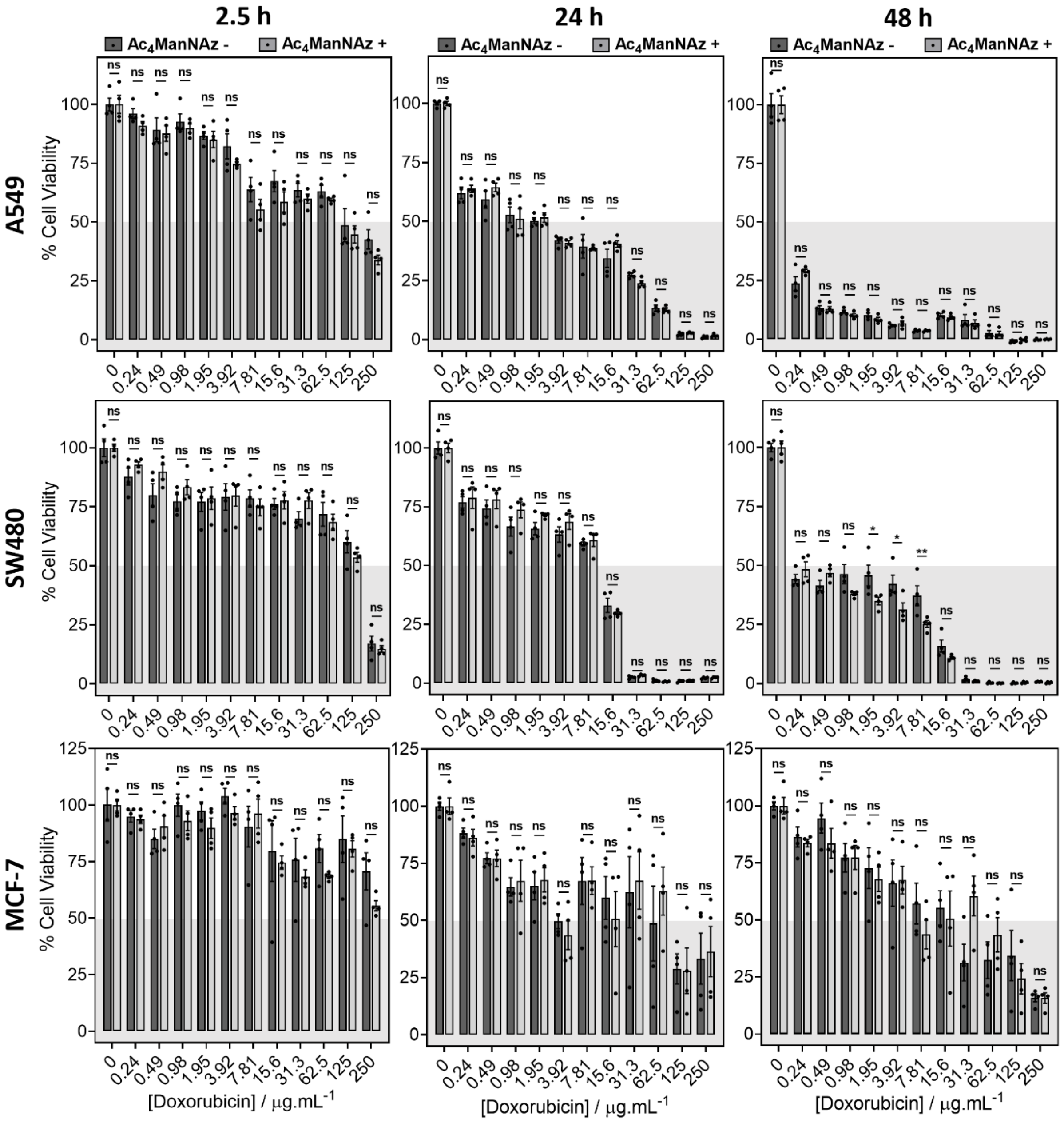

Figure S18. *Percentage cell viability of A549, SW480 and MCF-7 cells untreated (**dark grey**) or treated (**light grey**) with Ac4ManNAz (40 µM, 96 h) and treated with doxorubicin (0 – 250 µg.ml^{-1}, 2.5 h). Cell viability was determined by resazurin assays and data is presented as mean % cell viability relative to viable control cells ± SEM (n = 4) from four independent repeats. Statistical analysis was performed comparing % cell viability of Ac4ManNAz untreated and treated cells at equal doxorubicin concentrations. ns: $p \geq 0.05$, ** $p \leq 0.01$.*

2.3 Haemolysis Assays

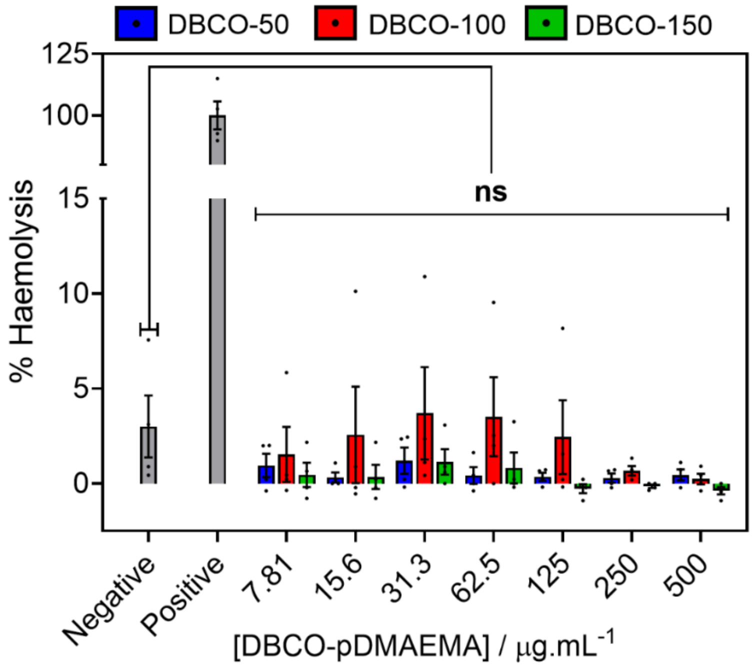

Figure S19. *Haemolysis studies of DBCO-pDMAEMA$_n$ (0 – 500 µg.ml^{-1}, 3 h) ranging in chain length (**DBCO-50**, **DBCO-100** and **DBCO-150**) against ovine blood determined by an AHD575 assay. A negative control of ovine blood incubated in DPBS and a positive control of ovine blood incubated in lysis buffer (3 h) have been provided. The data is presented as average percentage haemolysis relative to a lysis control ± SEM (n = 4) from four independent repeats. Statistical analysis was performed comparing all polymer treatments against blood incubated in DPBS alone (3 h). ns: $p \geq 0.05$.*

2.4 LDH Assay

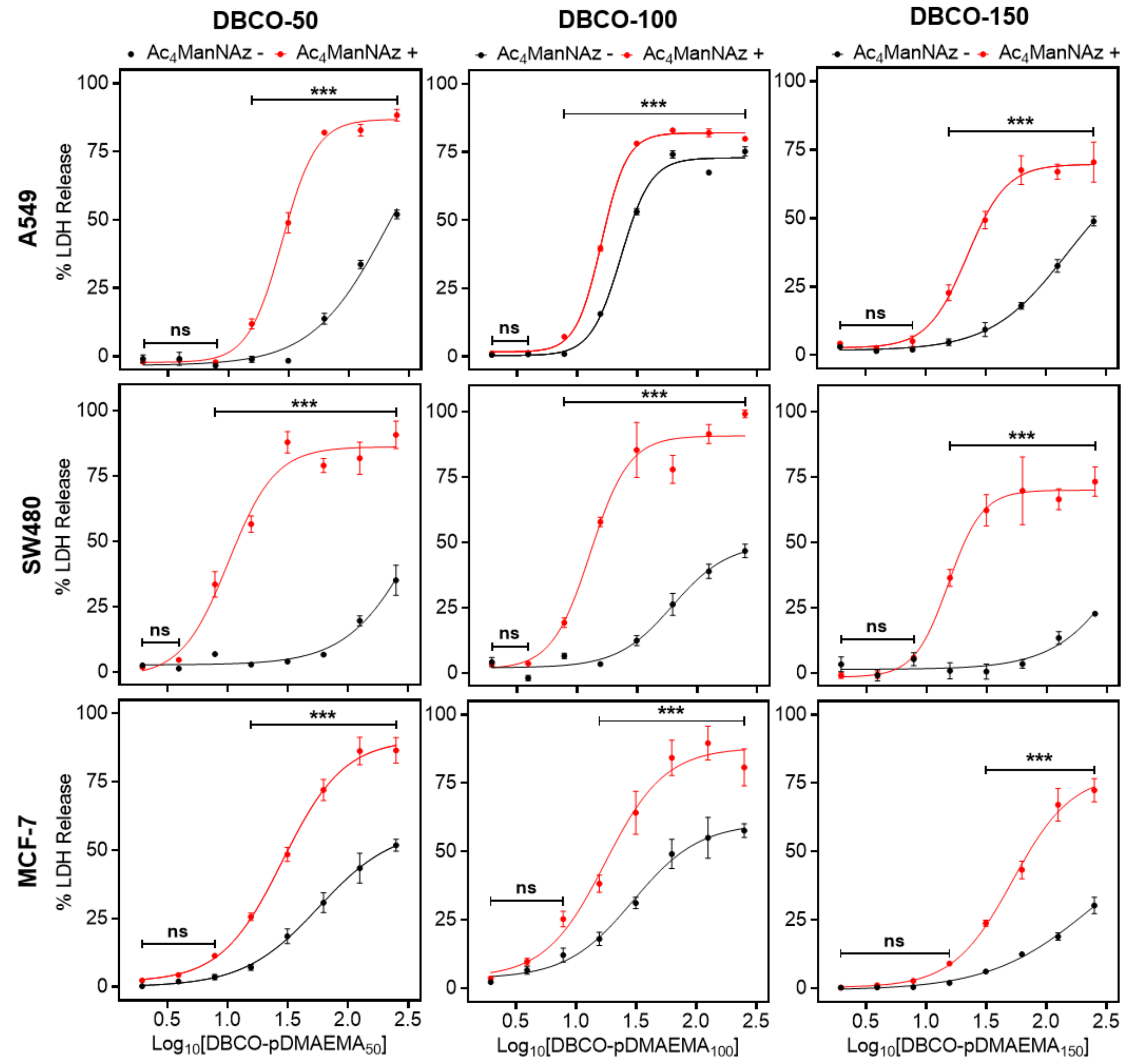

Figure S20. *Percentage lactate dehydrogenase (% LDH) release of cells (A549, SW480 and MCF-7) either untreated **(black)** or treated **(red)** with Ac4ManNAz (40 µM, 96 h) and subsequently treated with DBCO-pDMAEMA$_n$ (2 – 250 µg.ml^{-1}, 2.5 h) ranging in length **(DBCO-50**, **DBCO-100** and **DBCO-150**). Mean % LDH release was reported relative to a positive lysis control ± SEM (n = 3) from three independent repeats. Statistical analysis was performed comparing % LDH release of Ac4ManNAz untreated and treated cells at equal DBCO-pDMAEMA$_n$ concentrations. ns: $p \geq 0.05$, *** $p \leq 0.001$.*

2.5 Live/ Dead Assays

2.5.1 Live/ Dead Example Images

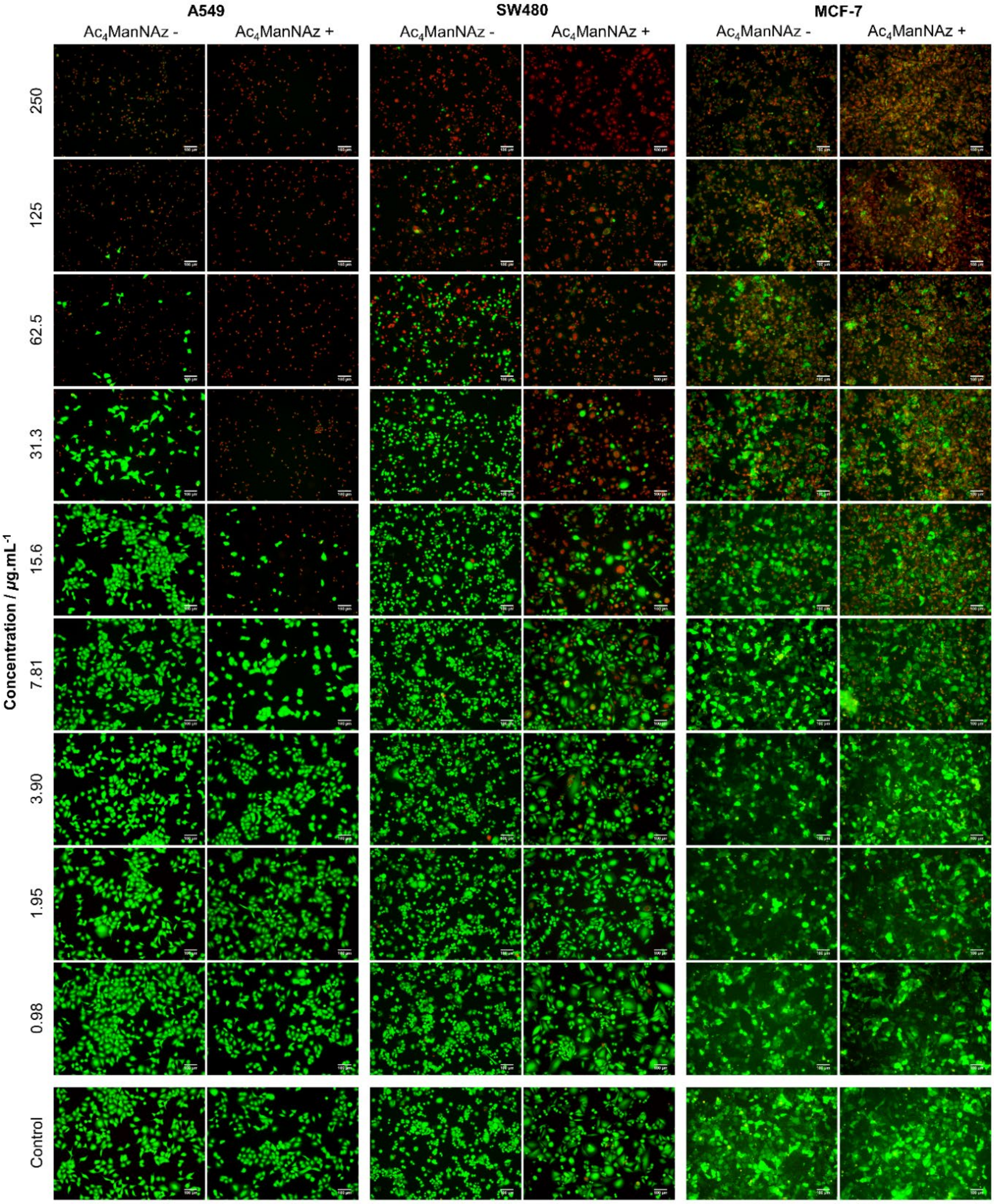

Figure S21. *Sample merged Live/ Dead images of 3 cell types (A549, SW480 and MCF-7) either untreated (-) or treated (+) with Ac4ManNAz (40 µM, 96 h) and subsequently treated with DBCO-pDMAEMA100 (**DBCO-100**, 1 – 250 µg.ml^{-1}, 2.5 h). Scale bar = 100 µm. Green = calcein (healthy). Red = ethidium iodide (dead).*

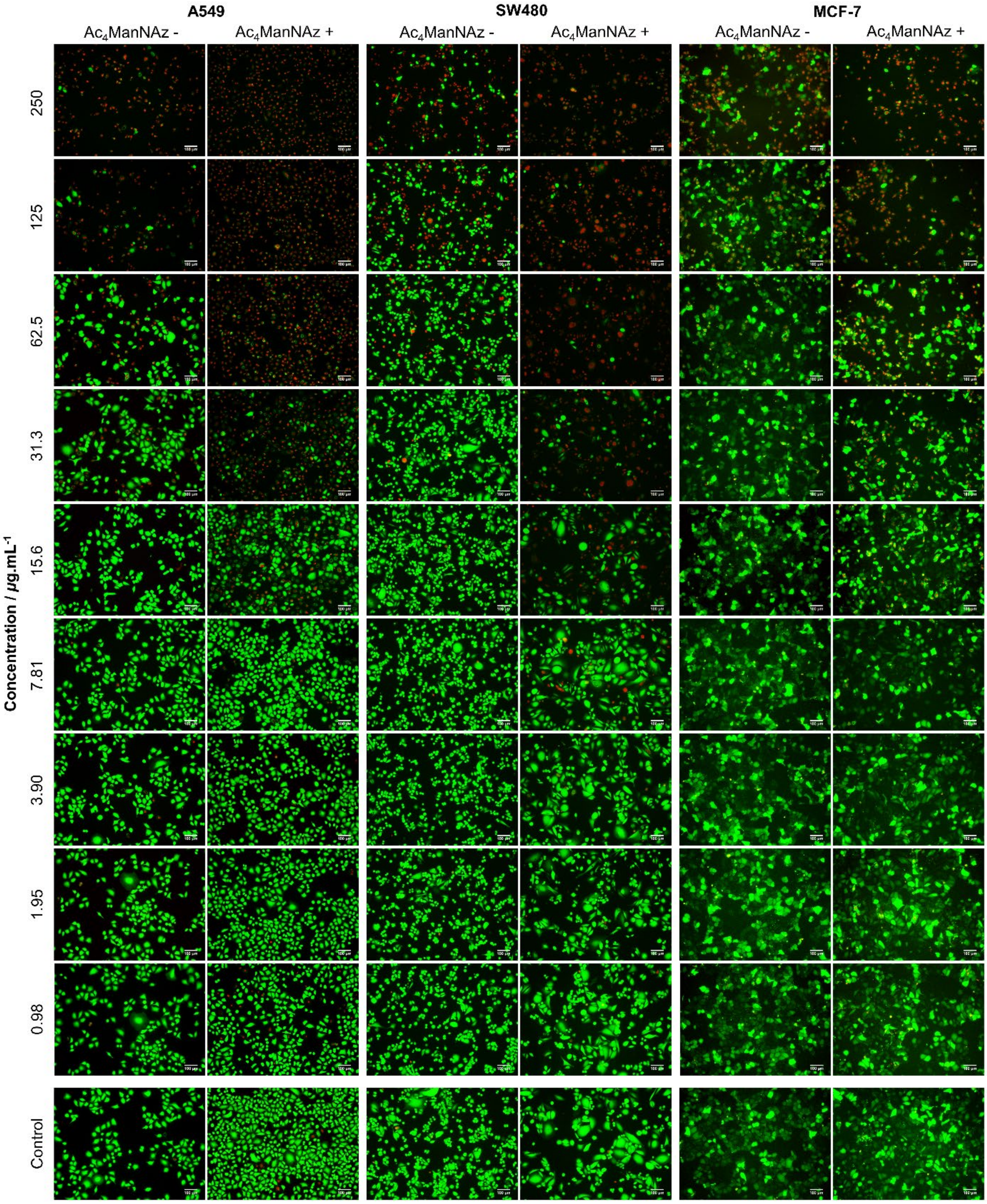

Figure S22. *Sample merged Live/ Dead images of 3 cell types (A549, SW480 and MCF-7) either untreated (-) or treated (+) with $Ac_4ManNAz$ (40 µM, 96 h) and subsequently treated with DBCO-pDMAEMA$_{150}$ (**DBCO-150**, 1 – 250 µg.ml^{-1}, 2.5 h). Scale bar = 100 µm. Green = calcein (healthy). Red = ethidium iodide (dead).*

2.5.2 Live/ Dead Percentage Live Cells

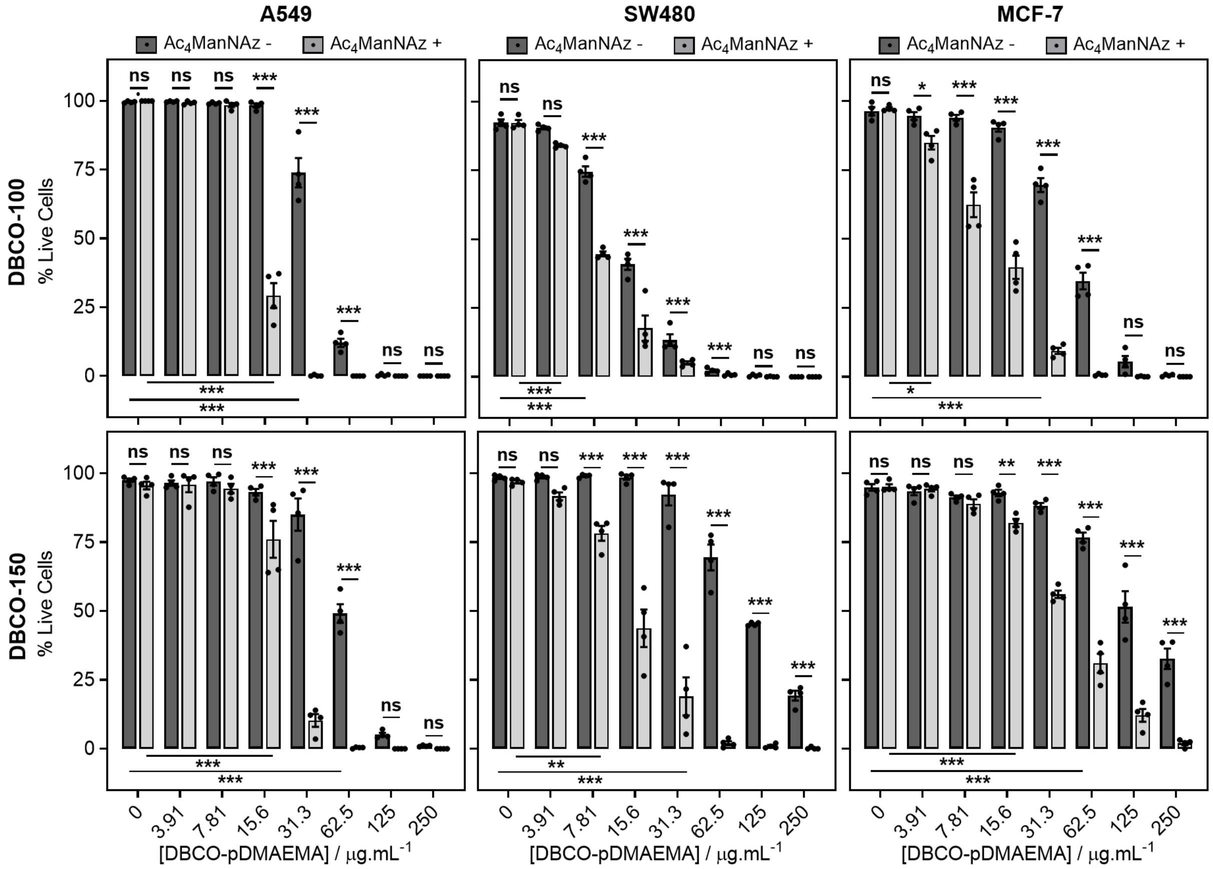

Figure S23. *Percentage live cell count of 3 cell types (A549, SW480 and MCF-7) either untreated (**dark grey**) or treated (**light grey**) with $Ac_4ManNAz$ (40 µM, 96 h) and subsequently treated with DBCO-pDMAEMA$_n$ (0 – 250 µg.ml^{-1}, 2.5 h) ranging in length (**DBCO-100** and **DBCO-150**). Percentage live cells was determined through live/ dead staining and reported relative to the total number of live and dead cells counts ± SEM (n = 4) from four independent repeats. Statistical analysis was performed by comparing each DBCO-pDMAEMA$_n$ concentration treatment with the respective negative control (**below bars**) and also comparing $Ac_4ManNAz$ treated and untreated cells at each individual polymer concentration treatment (**above bars**). ns: $p \geq 0.05$, * $p \leq 0.05$, ** $p \leq 0.01$, *** $p \leq 0.001$.*

2.5.3 Percentage Change in Live/ Dead EC_{50} Values

Table S5. *Percentage change in EC_{50} values of DBCO-pDMAEMA$_n$ with an Ac_4ManNAz pretreatment. EC_{50} values were determined from Live/ Dead dose-response curves (Fig. 2C, see article) and have been reported in Table 1 (see article).*

	A549		SW480		MCF-7	
	%	Fold	%	Fold	%	Fold
DBCO-100	65.1 ± 5.7	2.86 ± 0.15	75.9 ± 5.2	4.15 ± 0.41	42.0 ± 3.2	1.72 ± 0.07
DBCO-150	66.5 ± 2.1	2.99 ± 0.13	86.5 ± 4.2	7.38 ± 0.87	74.5 ± 13.3	3.93 ± 0.47

2.6 Annexin/ PI

2.6.1 Annexin/ PI Images

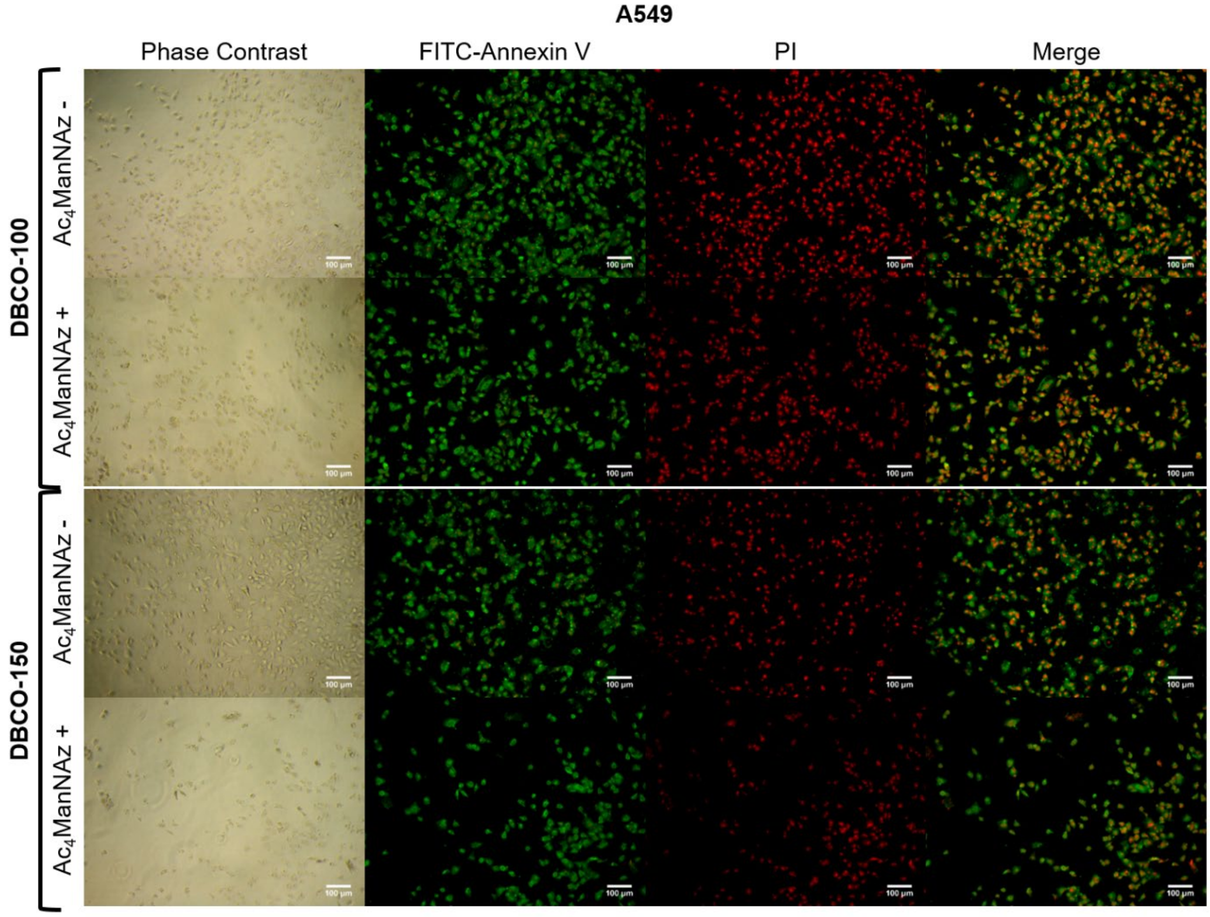

Figure S24. *A549 untreated (-) and treated (+) with Ac$_4$ManNAz (40 µM, 96 h) and treated with DBCO-pDMAEMA$_{100/150}$ (**DBCO-100** or **DBCO-150**, 2.5 h) were stained with FITC Annexin V and PI immediately after the treatments. The concentrations used were determined using the resazurin assay (Fig. 2A) at the point where cell viability was reduced by 90% (EC$_{90}$). Scale bar = 100 µm. Green = FITC Annexin V. Red = propidium iodide (PI).*

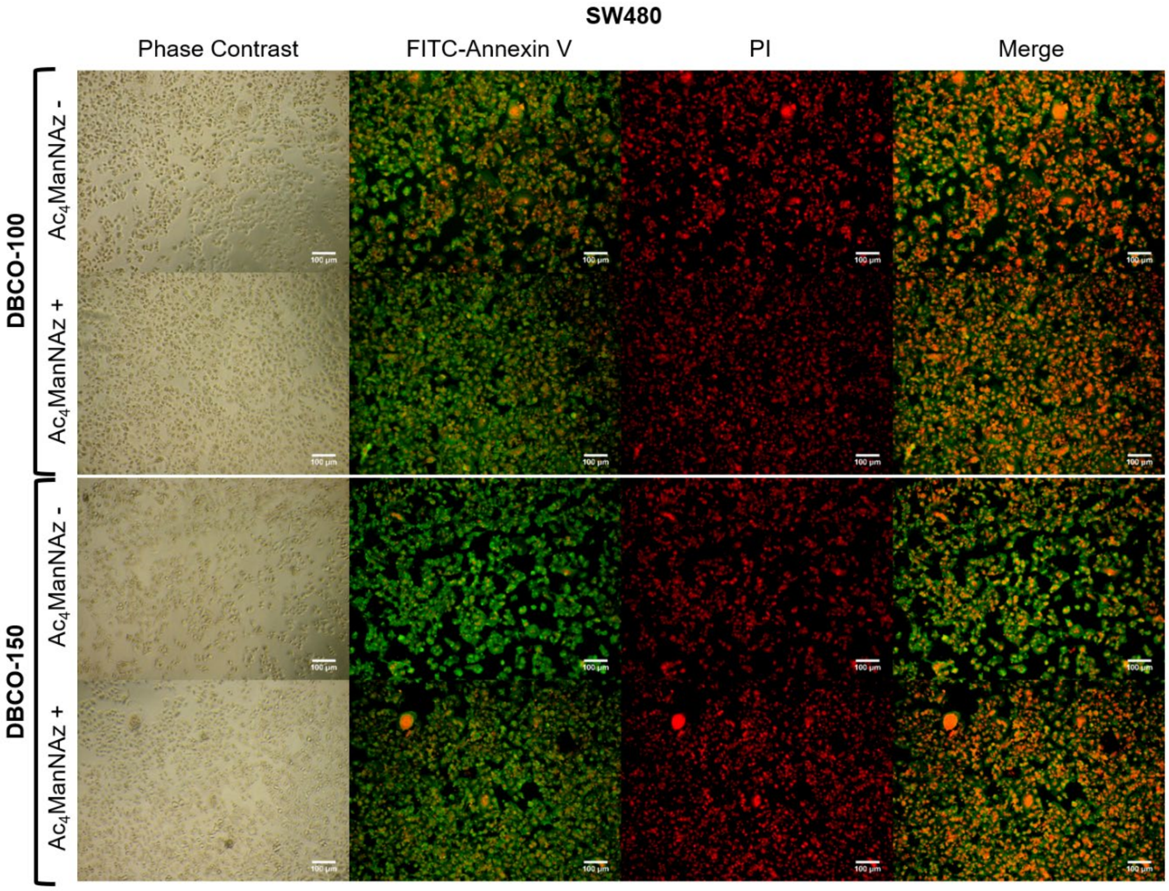

Figure S25. *SW480 untreated (-) and treated (+) with Ac$_4$ManNAz (40 µM, 96 h) and treated with DBCO-pDMAEMA$_{100/150}$ (**DBCO-100** or **DBCO-150**, 2.5 h) were stained with FITC Annexin V and PI immediately after the treatments. The concentrations used were determined using the resazurin assay (Fig. 2A) at the point where cell viability was reduced by 90% (EC$_{90}$). Scale bar = 100 µm. Green = FITC Annexin V. Red = propidium iodide (PI).*

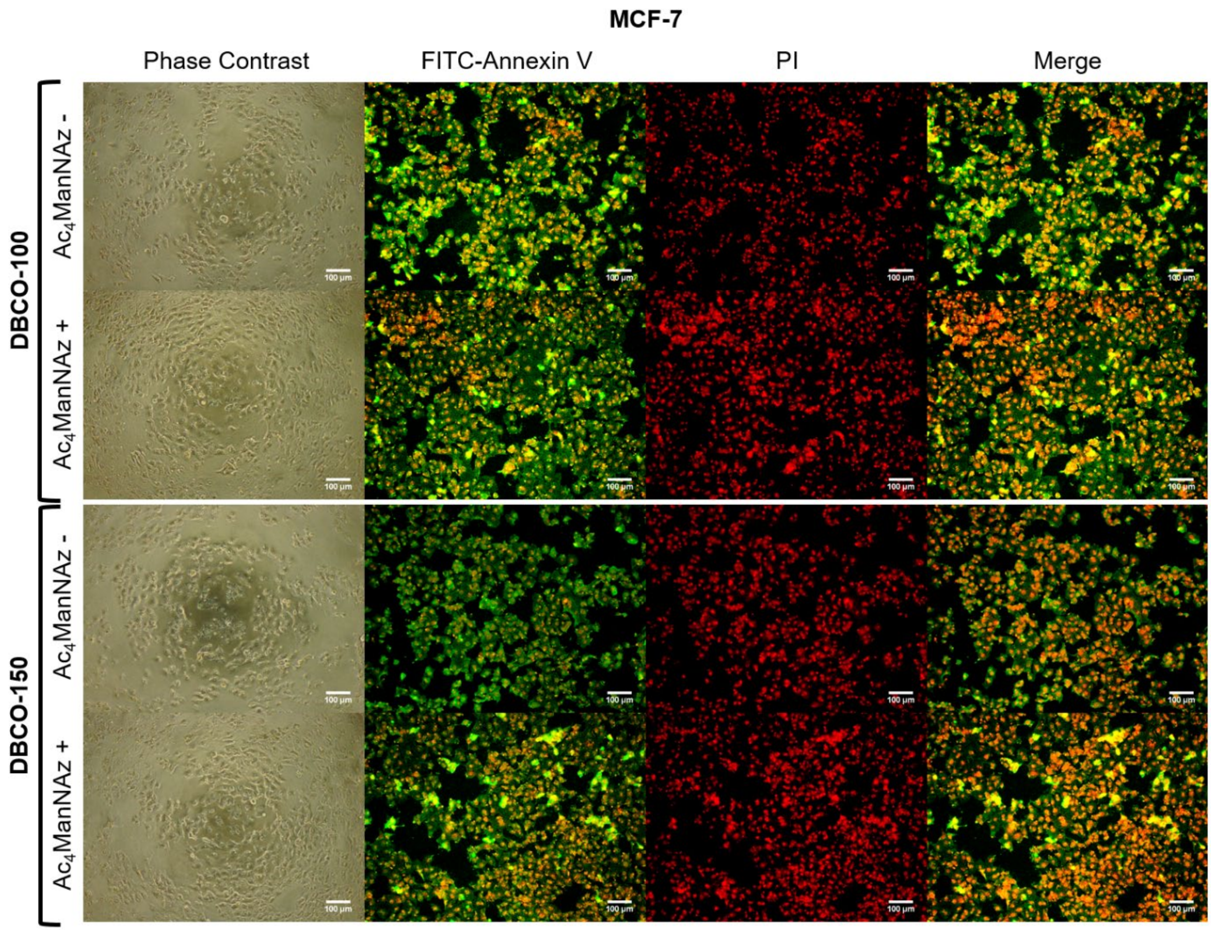

Figure S26. *MCF-7 untreated (-) and treated (+) with Ac$_4$ManNAz (40 µM, 96 h) and treated with DBCO-pDMAEMA$_{100/150}$ (**DBCO-100** or **DBCO-150**, 2.5 h) were stained with FITC Annexin V and PI immediately after the treatments. The concentrations used were determined using the resazurin assay (Fig. 2A) at the point where cell viability was reduced by 90% (EC$_{90}$). Scale bar = 100 µm. Green = FITC Annexin V. Red = propidium iodide (PI).*

2.7 Caspase-3/7 Kinetics Apoptosis Assay

2.7.1 Caspase-3/7 example images

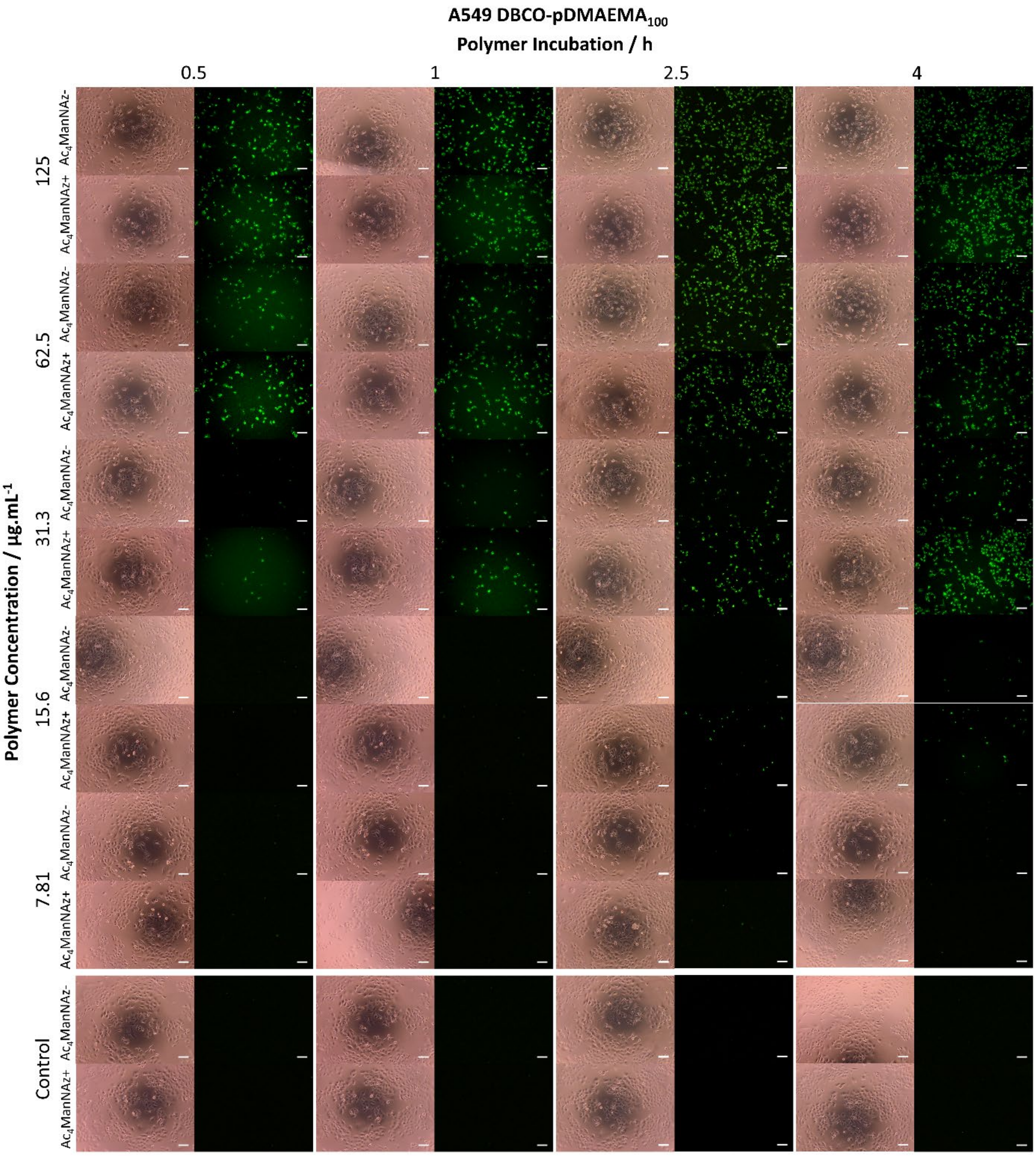

Figure S27. *Sample images of A549 cells treated (+) or untreated (-) with Ac4ManNAz (40 μM, 96 h) and incubated with DBCO-pDMAEMA100 (**DBCO-100**, 0 – 125 μg.ml^{-1}) and CellEvent Caspase-3/7 Detection Reagent for 4h. Images were taken at 0.5, 1, 2.5 and 4 h time points. Images of cells incubated with CellEvent Caspase-3/7 Detection Reagent alone in cell media were provided as control viable cells. Scale bar = 100 μm. Green = caspase positive cells.*

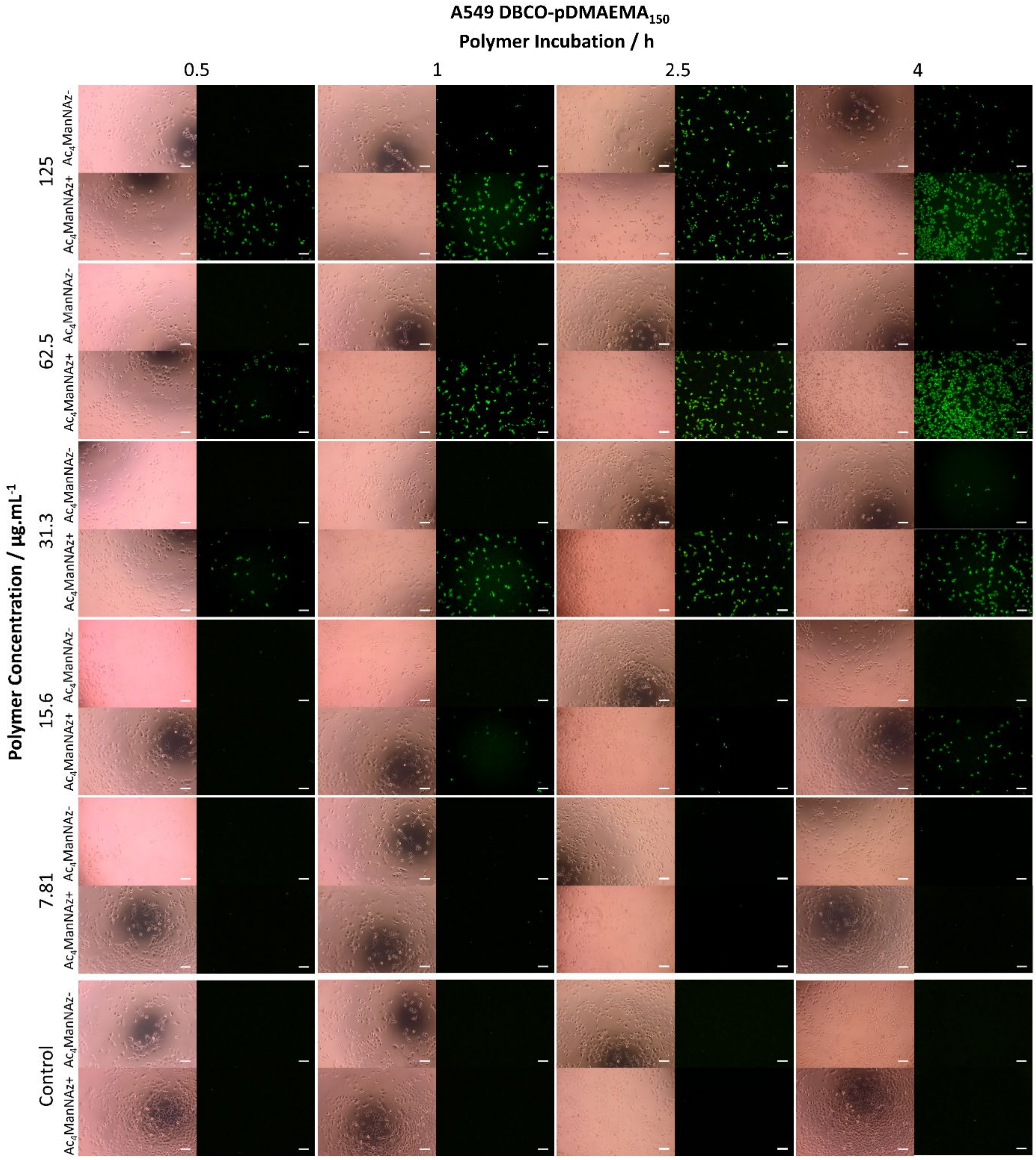

Figure S28. *Sample images of A549 cells treated (+) or untreated (-) with Ac4ManNAz (40 µM, 96 h) and incubated with DBCO-pDMAEMA150 (**DBCO-150**, 0 – 125 µg.ml^{-1}) and CellEvent Caspase-3/7 Detection Reagent for 4h. Images were taken at 0.5, 1, 2.5 and 4 h time points. Images of cells incubated with CellEvent Caspase-3/7 Detection Reagent alone in cell media were provided as control viable cells. Scale bar = 100 µm. Green = caspase positive cells.*

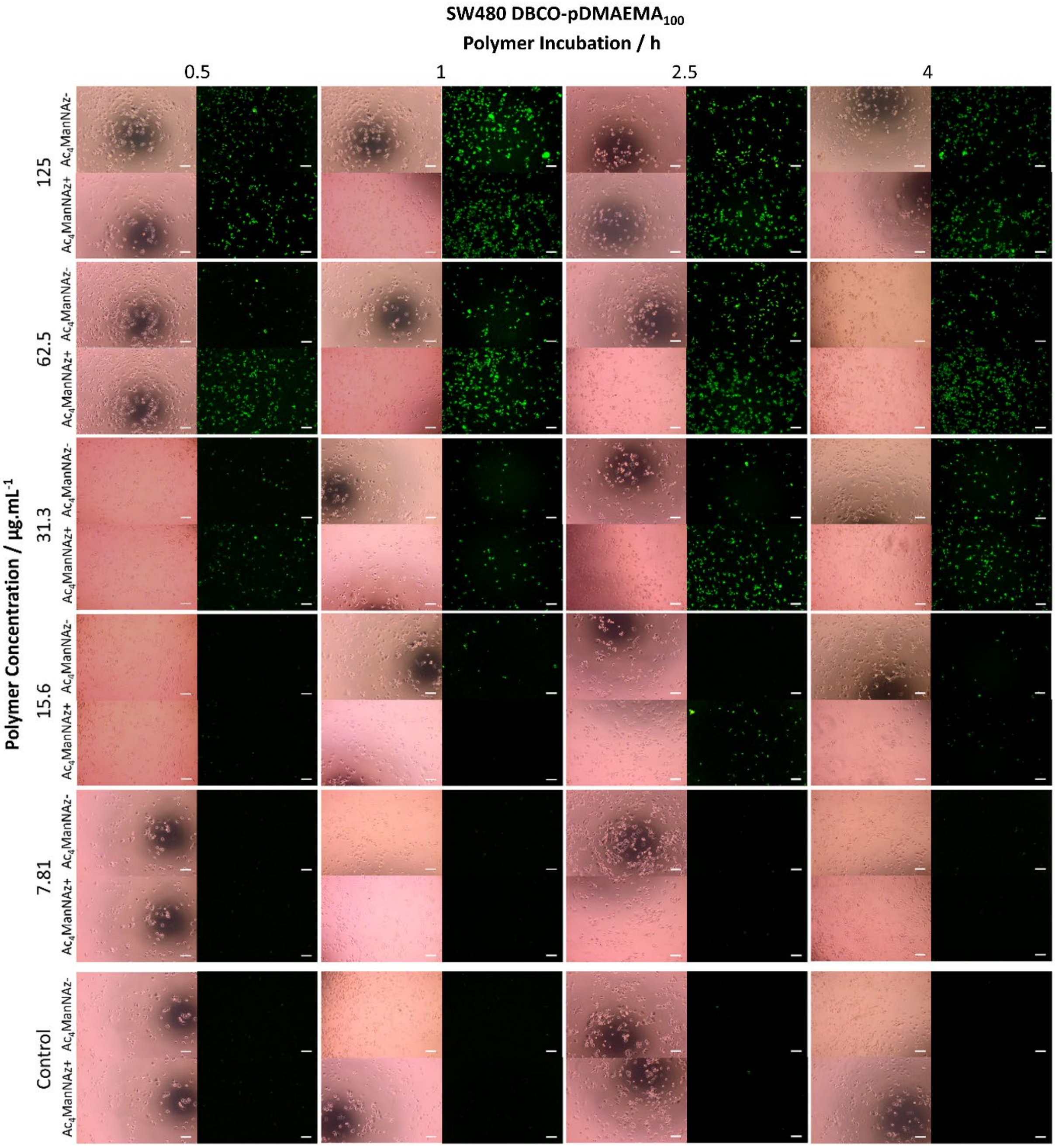

Figure S29. *Sample images of SW480 cells treated (+) or untreated (-) with Ac*$_4$*ManNAz (40 µM, 96 h) and incubated with DBCO-pDMAEMA*$_{100}$ *(**DBCO-100**, 0 – 125 µg.ml*$^{-1}$*) and CellEvent Caspase-3/7 Detection Reagent for 4h. Images were taken at 0.5, 1, 2.5 and 4 h time points. Images of cells incubated with CellEvent Caspase-3/7 Detection Reagent alone in cell media were provided as control viable cells. Scale bar = 100 µm. Green = caspase positive cells.*

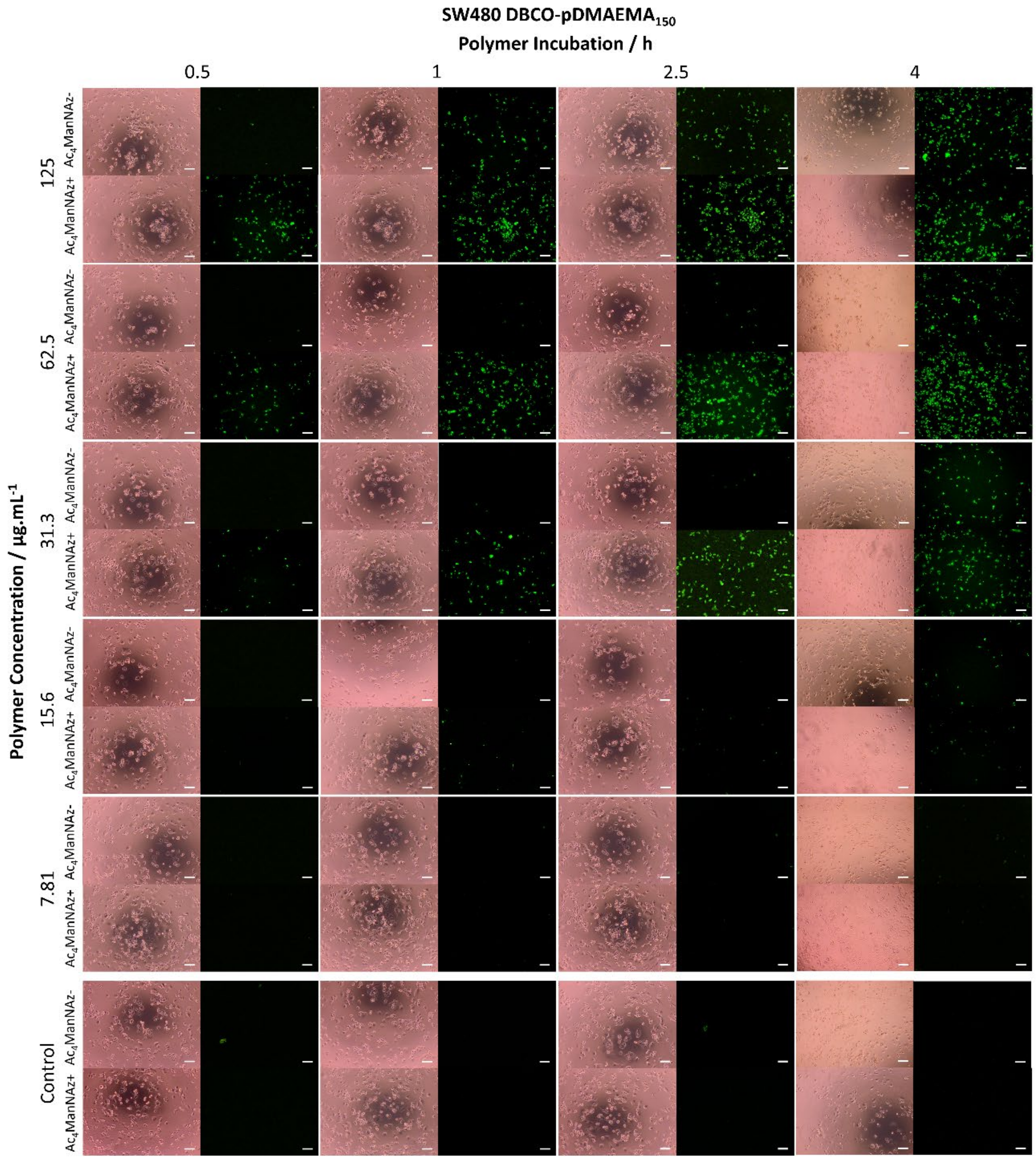

Figure S30. *Sample images of SW480 cells treated (+) or untreated (-) with Ac₄ManNAz (40 µM, 96 h) and incubated with DBCO-pDMAEMA₁₅₀ (**DBCO-150**, 0 – 125 µg.ml⁻¹) and CellEvent Caspase-3/7 Detection Reagent for 4h. Images were taken at 0.5, 1, 2.5 and 4 h time points. Images of cells incubated with CellEvent Caspase-3/7 Detection Reagent alone in cell media were provided as control viable cells. Scale bar = 100 µm. Green = caspase positive cells.*

2.7.2 Caspase EC_{50} Values

Table S6. *EC_{50} values of DBCO-pDMAEMA$_{100/150}$ (2.5 h incubation) against $Ac_4ManNAz$ treated (+) and untreated (-) A549 and SW480 cell lines determined by the percentage of caspase-3/7 positive cells seen in Figures S23 – S26.*

	A549		SW480	
	-	+	-	+
DBCO-100	72.4 ± 4.7	41.5 ± 3.0	68.0 ± 5.2	32.5 ± 1.6
DBCO-150	160.9 ± 10.7	54.4 ± 4.5	134.4 ± 4.2	34.6 ± 2.6

2.8 Polymer Grafting and Degrafting

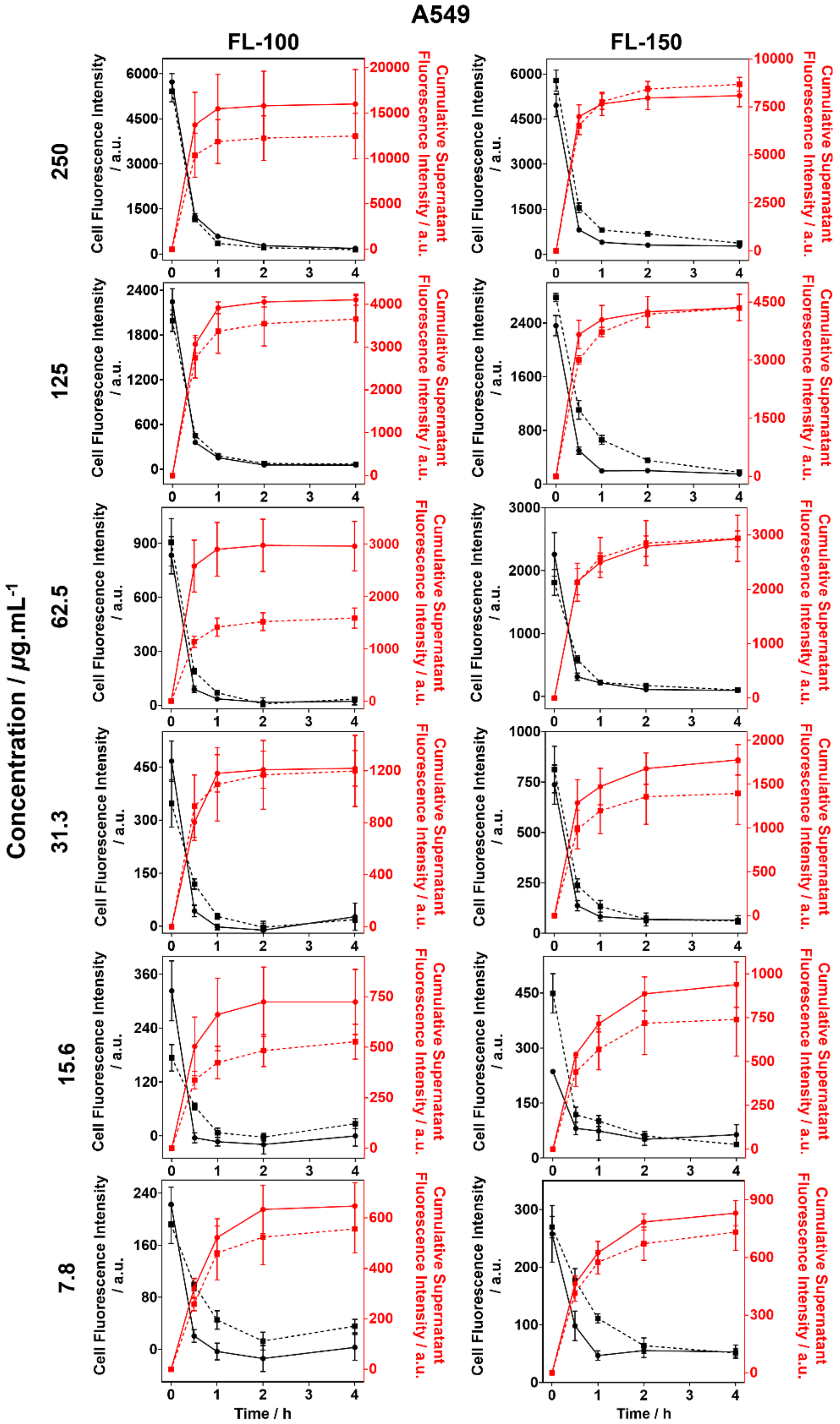

Figure S31. *Polymer degrafting from A549 cell surface. Fluorescence measurements of A549 cells either untreated (**solid line**) or treated (**dashed line**) with Ac$_4$ManNAz (40 µM, 96 h) and treated with DBCO-pDMAEMA$_n$-Fl (7.8 – 250 µg.ml^{-1}, 2.5 h) ranging in length (**FL-100** and **FL-150**) were recorded 0 – 4 h post-treatment (**black**). The supernatant was removed at each time point and the cumulative fluorescence intensity (**red**) was plotted. Data is reported as mean fluorescence intensity ± SEM (n = 4) from four independent repeats.*

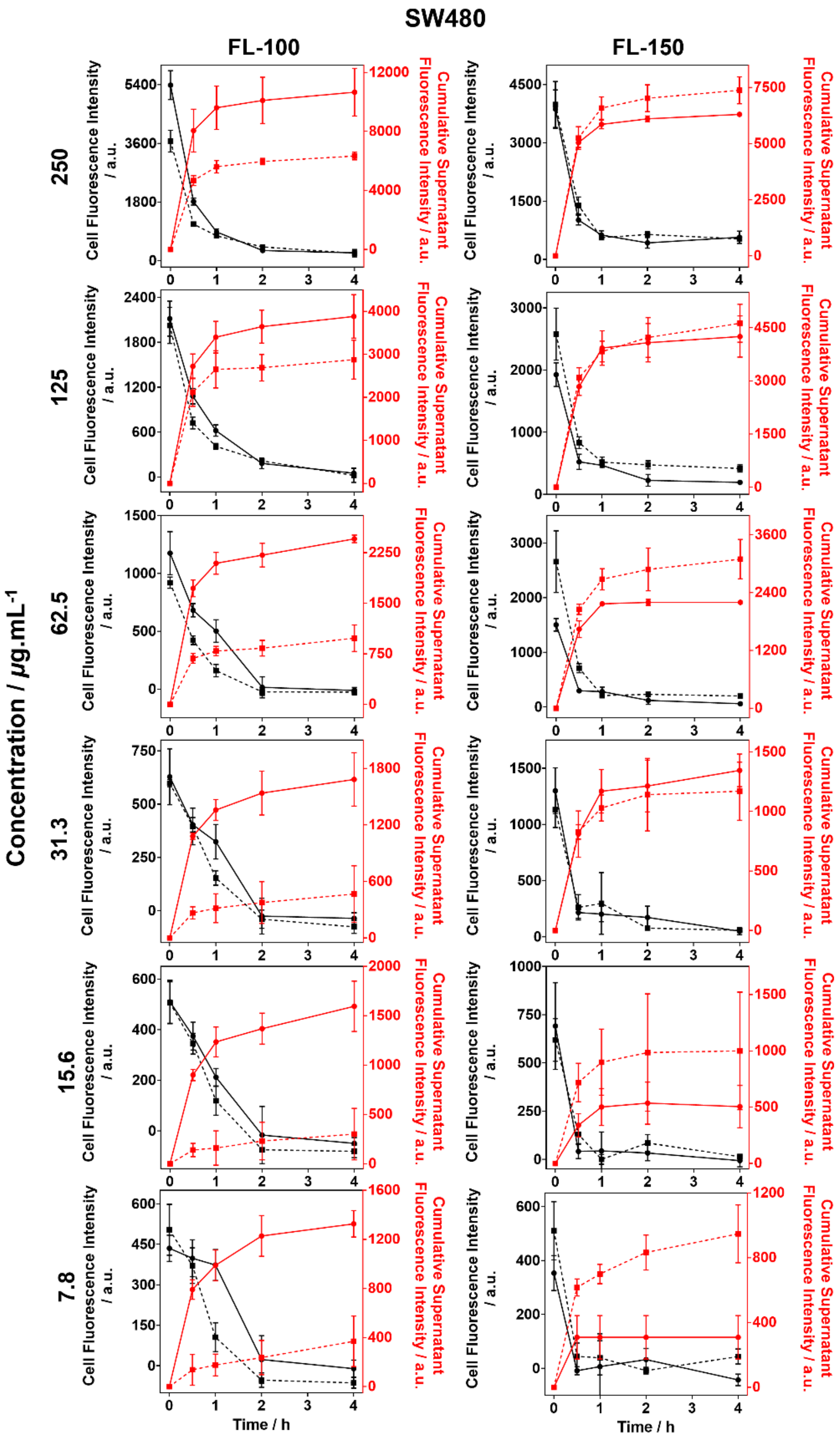

Figure S32. *Polymer degrafting from SW480 cell surface. Fluorescence measurements of A549 cells either untreated* (**solid line**) *or treated* (**dashed line**) *with Ac*$_4$*ManNAz (40 µM, 96 h) and treated with DBCO-pDMAEMA*$_n$*-Fl (7.8 – 250 µg.ml*$^{-1}$*, 2.5 h) ranging in length (**FL-100** and **FL-150**) were recorded 0 – 4 h post-treatment* (**black**)*. The supernatant was removed at each time point and the cumulative fluorescence intensity* (**red**) *was plotted. Data is reported as mean fluorescence intensity ± SEM (n = 4) from four independent repeats.*

2.9 Confocal Monolayer – Polymer Grafting and Membrane Damage

2.9.1 Polymer Grafting Maximum Intensity Projection Images

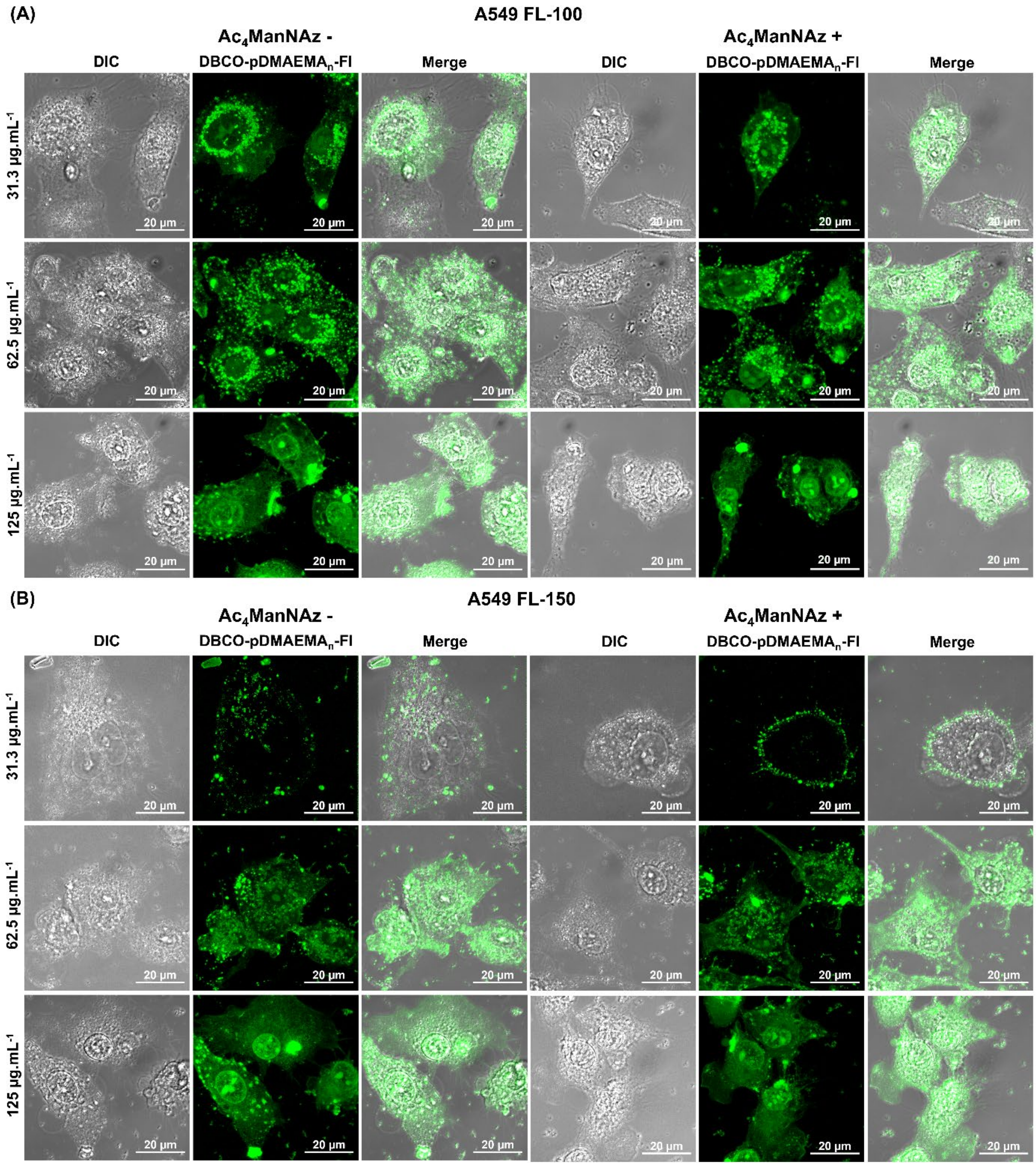

Figure S33. *Maximum intensity projection confocal images of A549 cells treated with (+) and without (-) $Ac_4ManNAz$ (40 µM, 96 h) and treated with either (**A**) DBCO-pDMAEMA$_{100}$-Fl (**FL-100**) or (**B**) DBCO-pDMAEMA$_{150}$-Fl (**FL-150**) (31.3 – 125 µg.ml^{-1}, 2.5 h). Scale bar = 20 µm. Green = DBCO-pDMAEMA$_n$-Fl.*

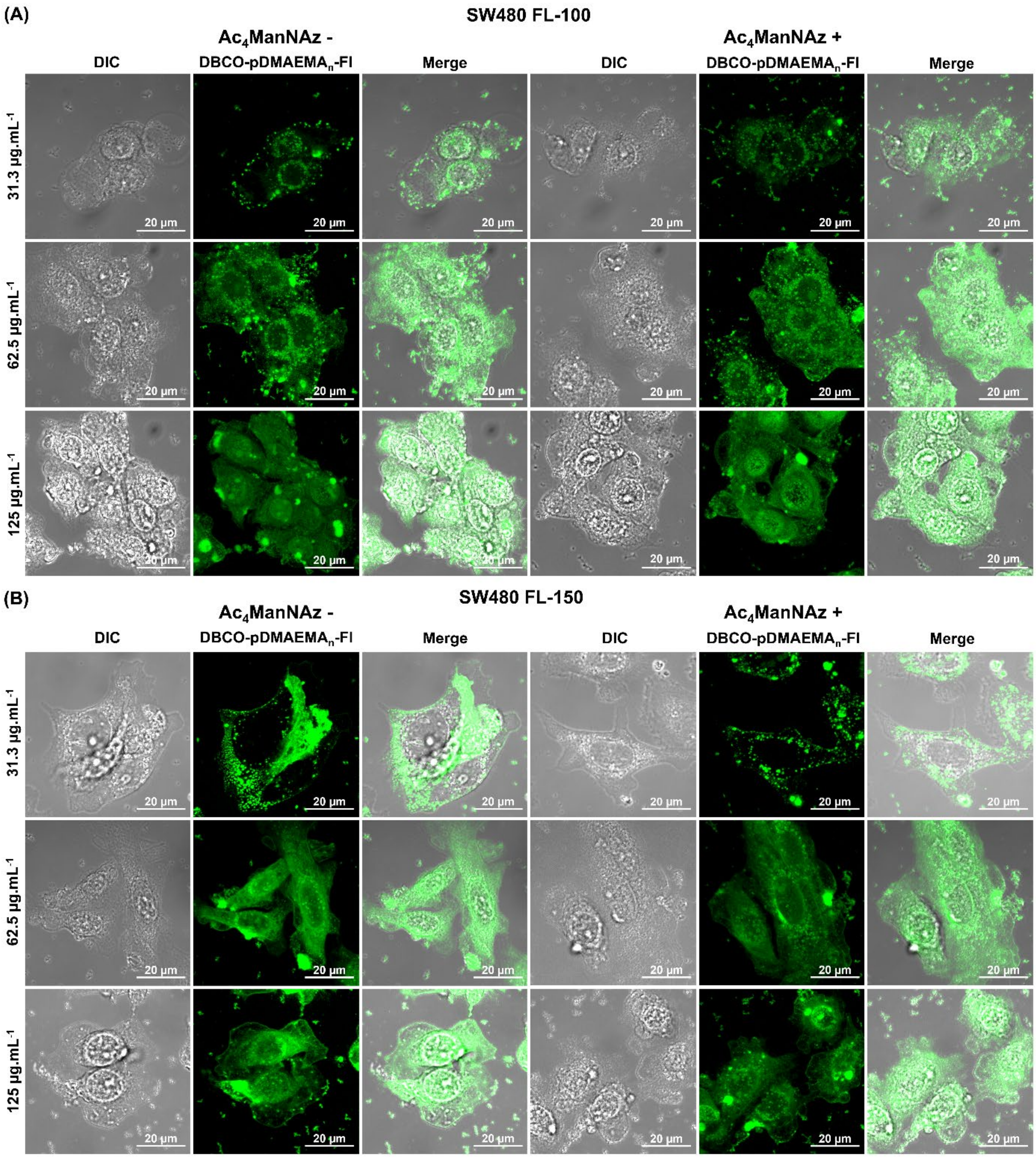

Figure S34. *Maximum intensity projection confocal images of SW480 cells treated with* (+) *and without* (**-**) *Ac₄ManNAz (40 µM, 96 h) and treated with either* (**A**) *DBCO-pDMAEMA*$_{100}$*-Fl* (***FL-100***) *or* (**B**) *DBCO-pDMAEMA*$_{150}$*-Fl* (***FL-150***) *(31.3 – 125 µg.ml*$^{-1}$*, 2.5 h). Scale bar = 20 µm. Green = DBCO-pDMAEMA*$_{n}$*-Fl.*

2.9.2 Membrane Staining Maximum Intensity Projection Images

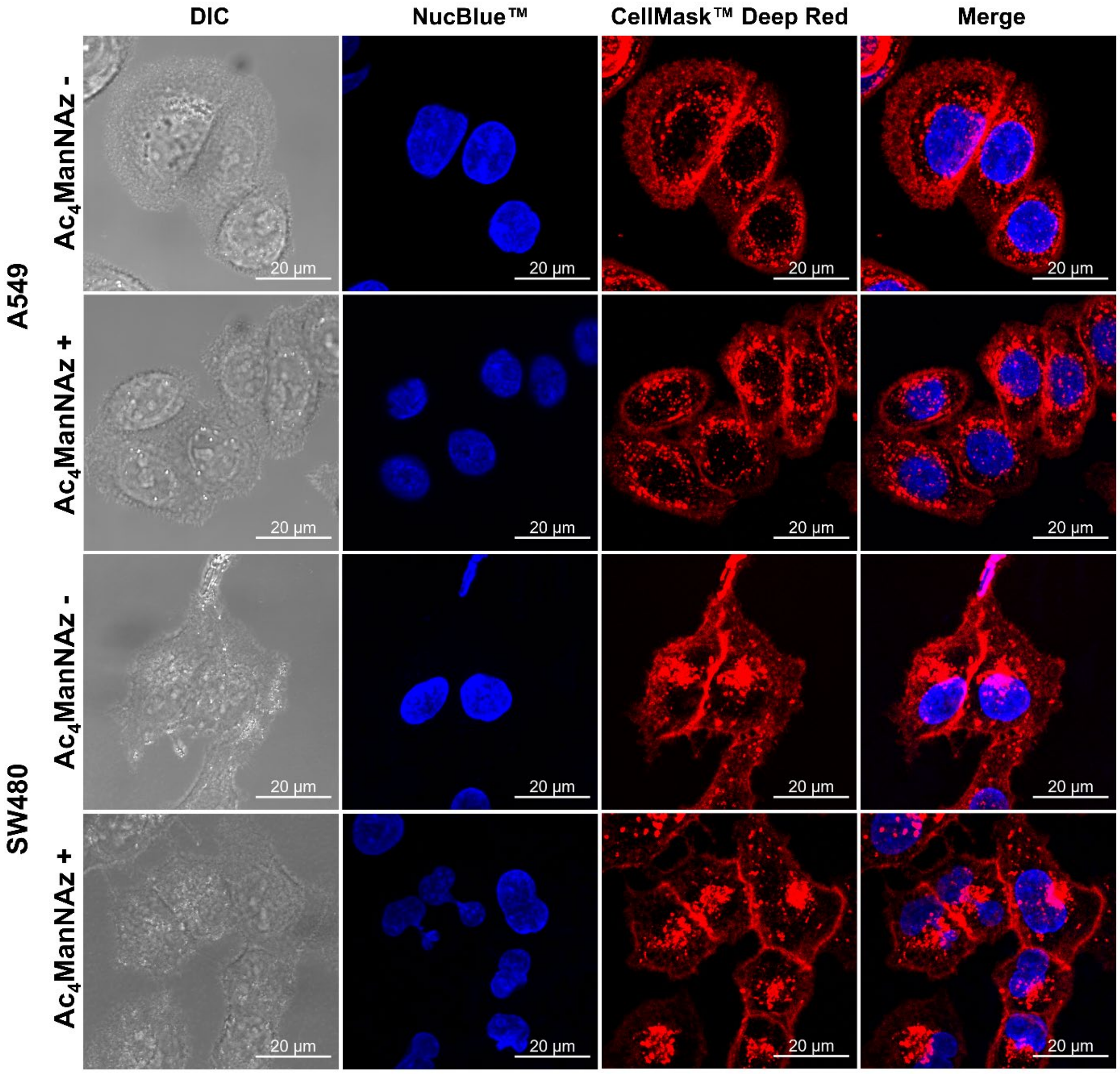

Figure S35. *Maximum intensity projection confocal images of A549 and SW480 cells treated with (+) and without (-) Ac$_4$ManNAz (40 µM, 96 h) and stained with CellMask^{TM} membrane stain (red) and NucBlue^{TM} nuclear stain (blue) as control viable cell images to demonstrate the extent of membrane damage in polymer treated cells. Scale bar = 20 µm.*

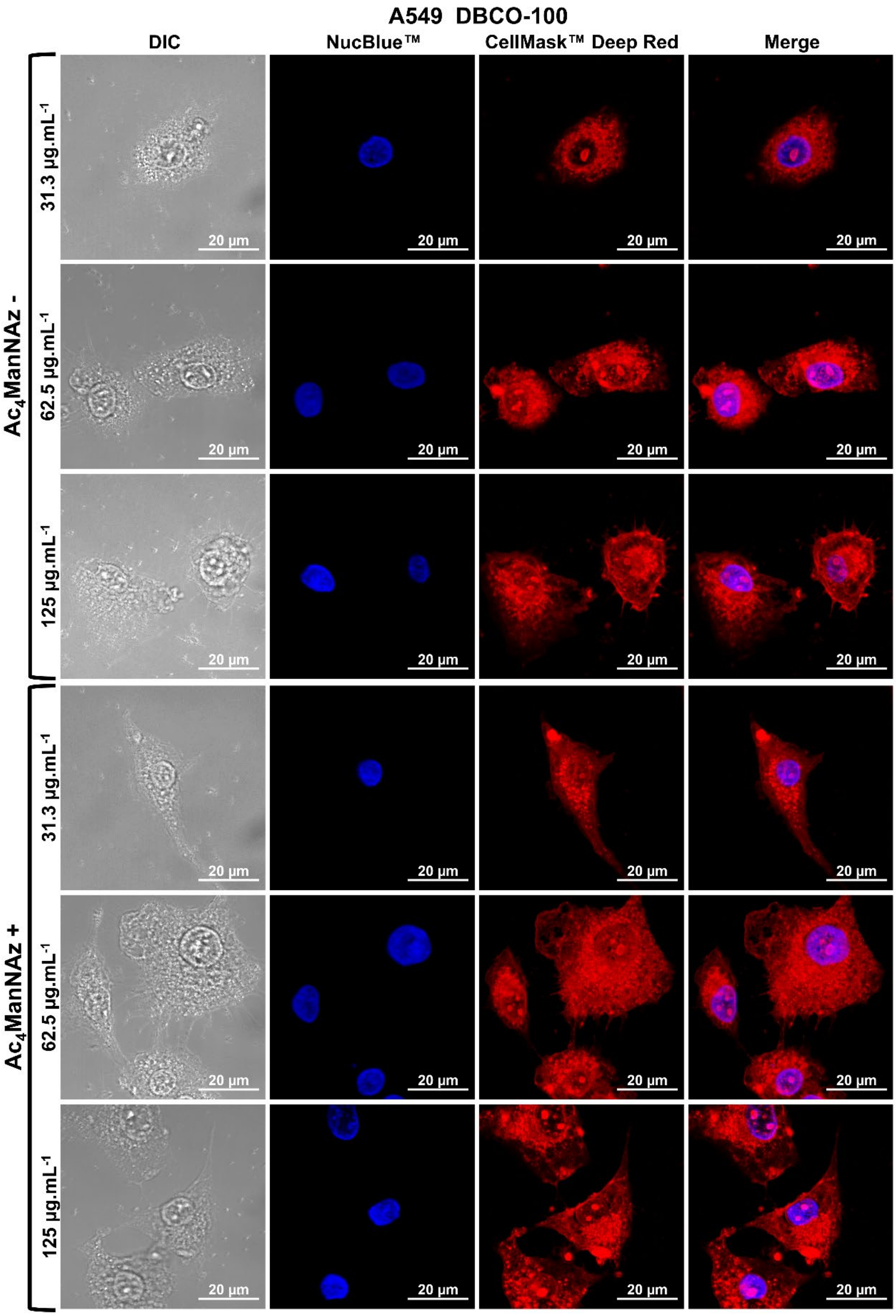

Figure S36. *Maximum intensity projection confocal images of A549 cells treated with (+) and without (-) Ac$_4$ManNAz (40 µM, 96 h) and stained with CellMask^{TM} membrane stain (red) and NucBlue^{TM} nuclear stain (blue) following treatment with DBCO-pDMAEMA$_{100}$ (**DBCO-100**, 31.3 – 125 µg.ml^{-1}, 2.5 h). Scale bar = 20 µm.*

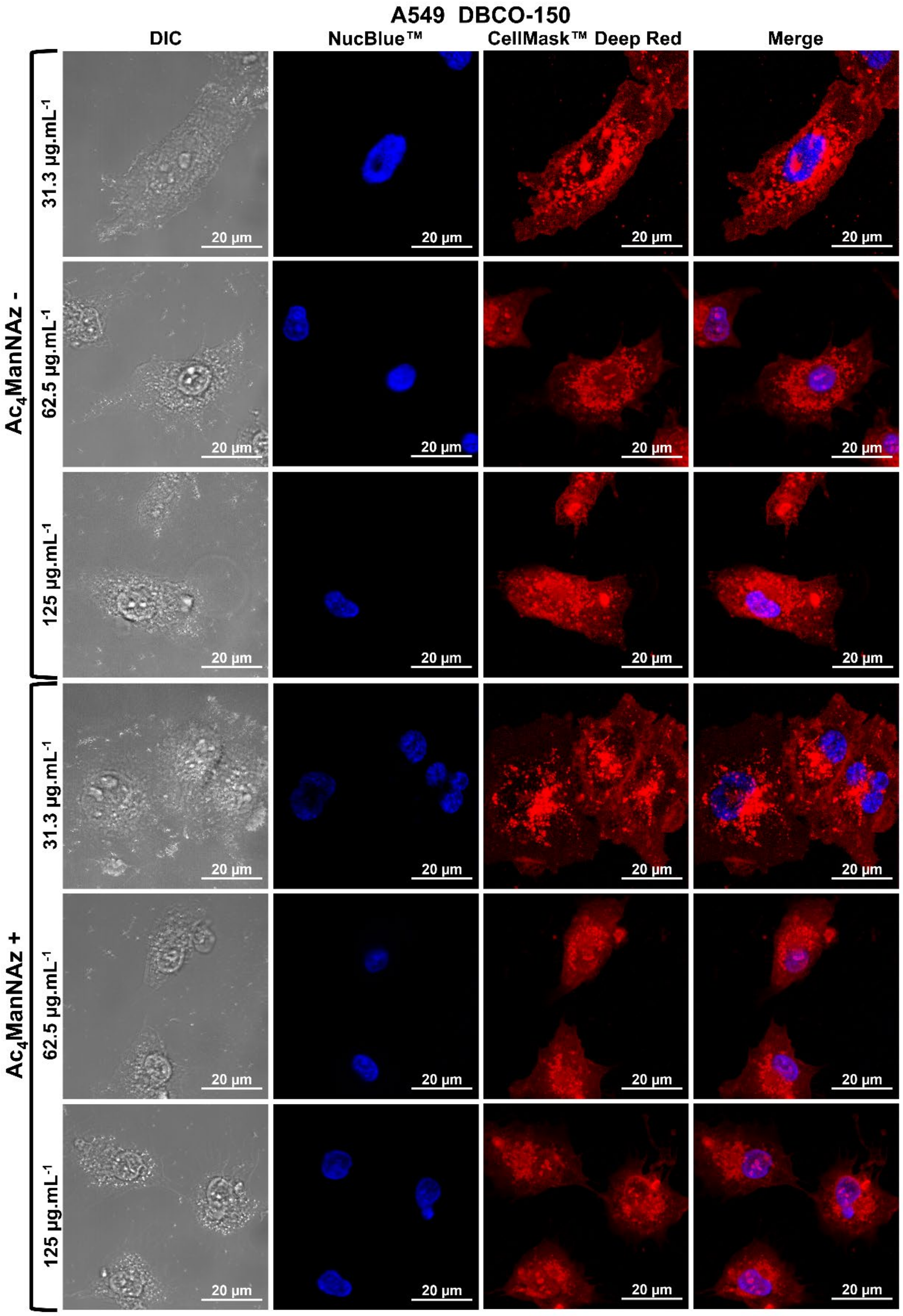

Figure S37. *Maximum intensity projection confocal images of A549 cells treated with (+) and without (-) Ac*$_4$*ManNAz (40 µM, 96 h) and stained with CellMask*^{TM} *membrane stain (red) and NucBlue*^{TM} *nuclear stain (blue) following treatment with DBCO-pDMAEMA*$_{150}$ *(**DBCO-150**, 31.3 – 125 µg.ml*$^{-1}$*, 2.5 h). Scale bar = 20 µm.*

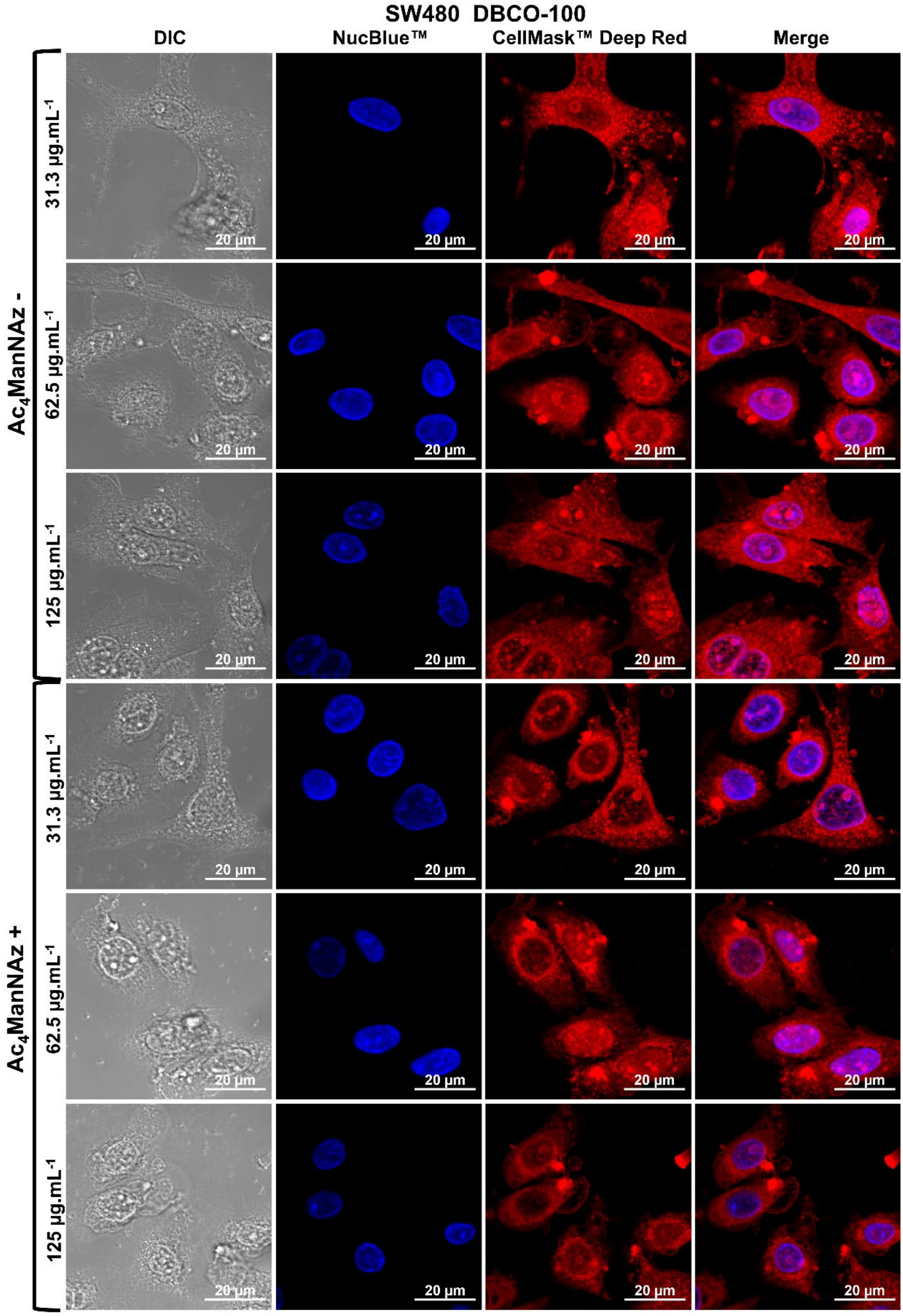

Figure S38. *Maximum intensity projection confocal images of SW480 cells treated with (+) and without (-) Ac4ManNAz (40 µM, 96 h) and stained with CellMask^{TM} membrane stain (red) and NucBlue^{TM} nuclear stain (blue) following treatment with DBCO-pDMAEMA$_{100}$ (**DBCO-100**, 31.3 – 125 µg.ml^{-1}, 2.5 h). Scale bar = 20 µm.*

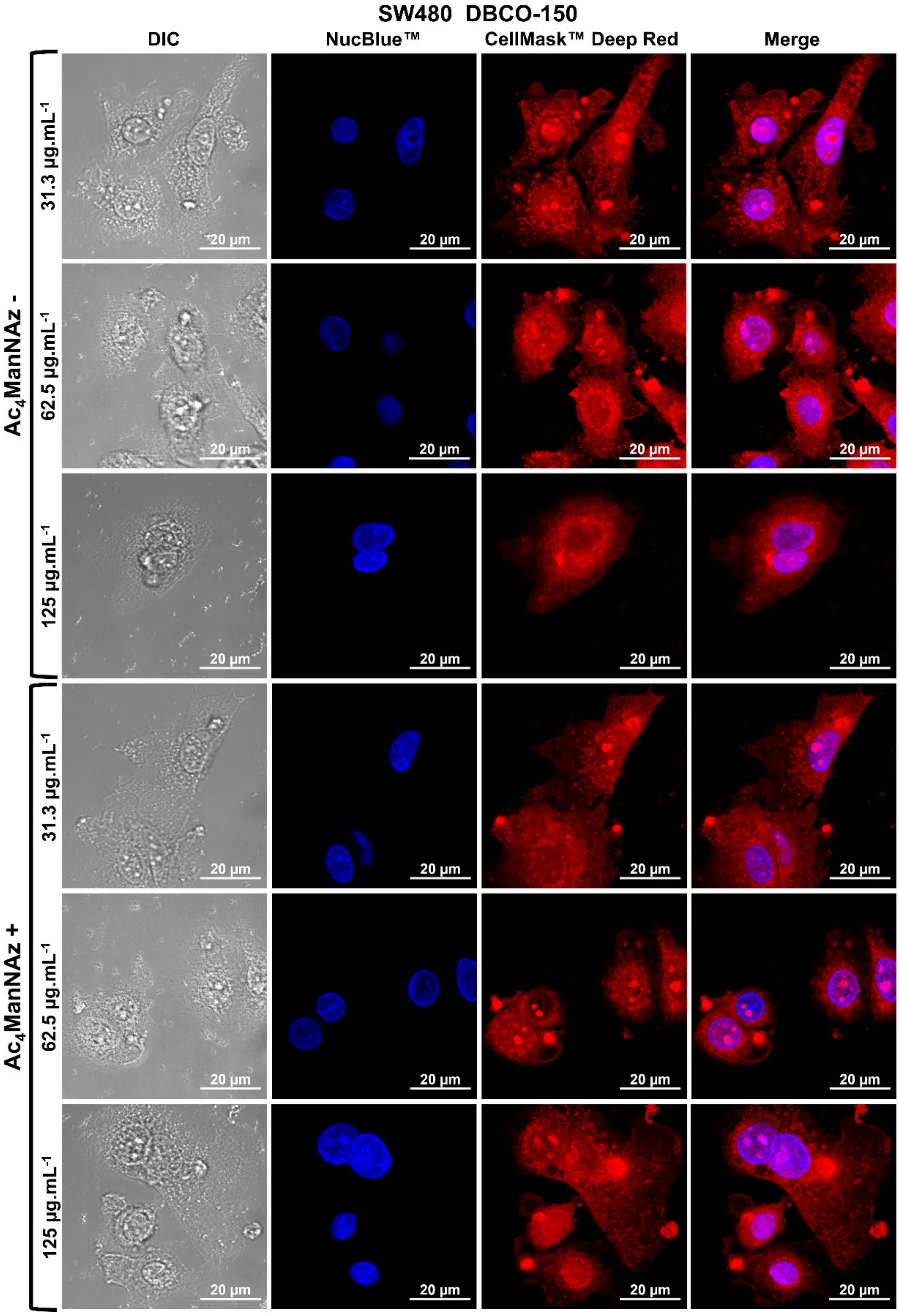

Figure S39. *Maximum intensity projection confocal images of SW480 cells treated with (+) and without (-) $Ac_4ManNAz$ (40 µM, 96 h) and stained with CellMask^{TM} membrane stain (red) and NucBlue^{TM} nuclear stain (blue) following treatment with DBCO-pDMAEMA$_{150}$ (**DBCO-150**, 31.3 – 125 µg.ml^{-1}, 2.5 h). Scale bar = 20 µm.*

2.9.3 Summary Confocal Images and 3D Reconstruction

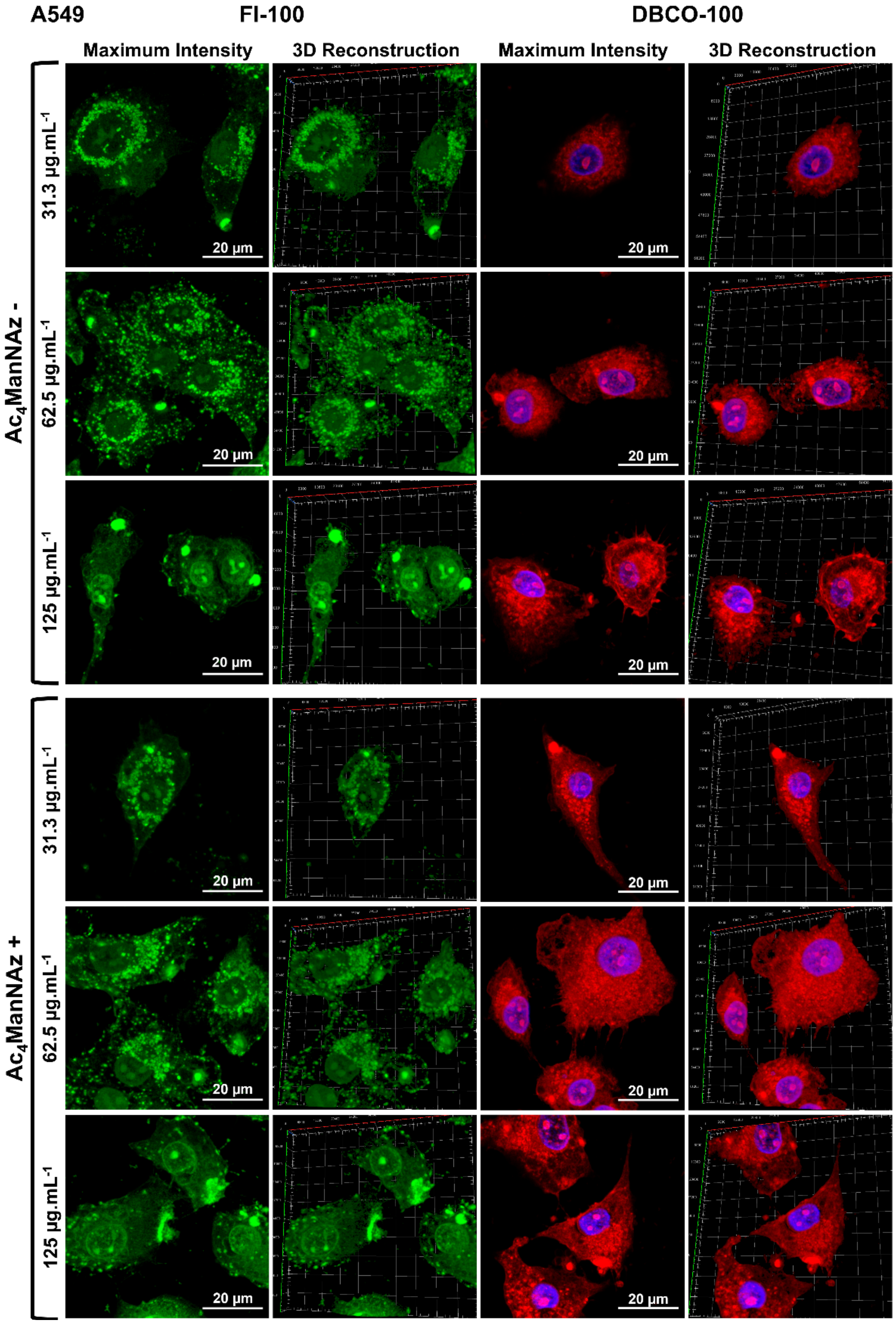

Figure S40. *Summary maximum intensity projection and 3D reconstructed confocal images of A549 cells treated with (+) and without (-) $Ac_4ManNAz$ (40 µM, 96 h) and treated with DBCO-pDMAEMA$_{100}$-Fl (**FL-100**, 31.3 – 125 µg.ml^{-1}, 2.5 h, Green). Images were also taken of cells treated with DBCO-pDMAEMA$_{100}$ (**DBCO-100**, 31.3 – 125 µg.ml^{-1}, 2.5 h) and stained with CellMask™ membrane stain (red) and NucBlue™ stain (blue). Scale bar = 20 µm.*

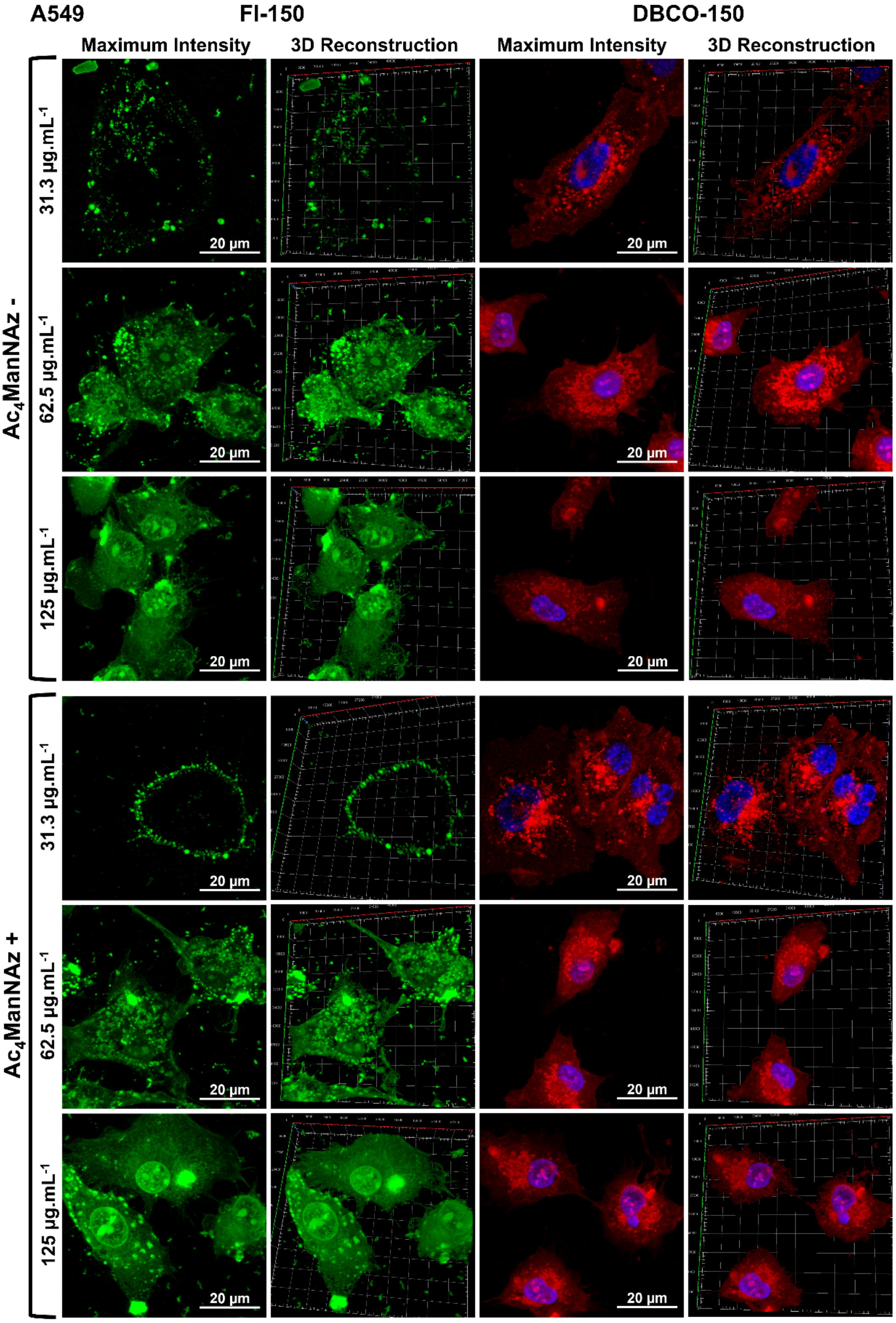

Figure S41. *Summary maximum intensity projection and 3D reconstructed confocal images of A549 cells treated with (+) and without (-) Ac$_4$ManNAz (40 µM, 96 h) and treated with DBCO-pDMAEMA$_{150}$-Fl (**FL-150**, 31.3 – 125 µg.ml^{-1}, 2.5 h, Green). Images were also taken of cells treated with DBCO-pDMAEMA$_{150}$ (**DBCO-150**, 31.3 – 125 µg.ml^{-1}, 2.5 h) and stained with CellMask^{TM} membrane stain (red) and NucBlue^{TM} stain (blue). Scale bar = 20 µm.*

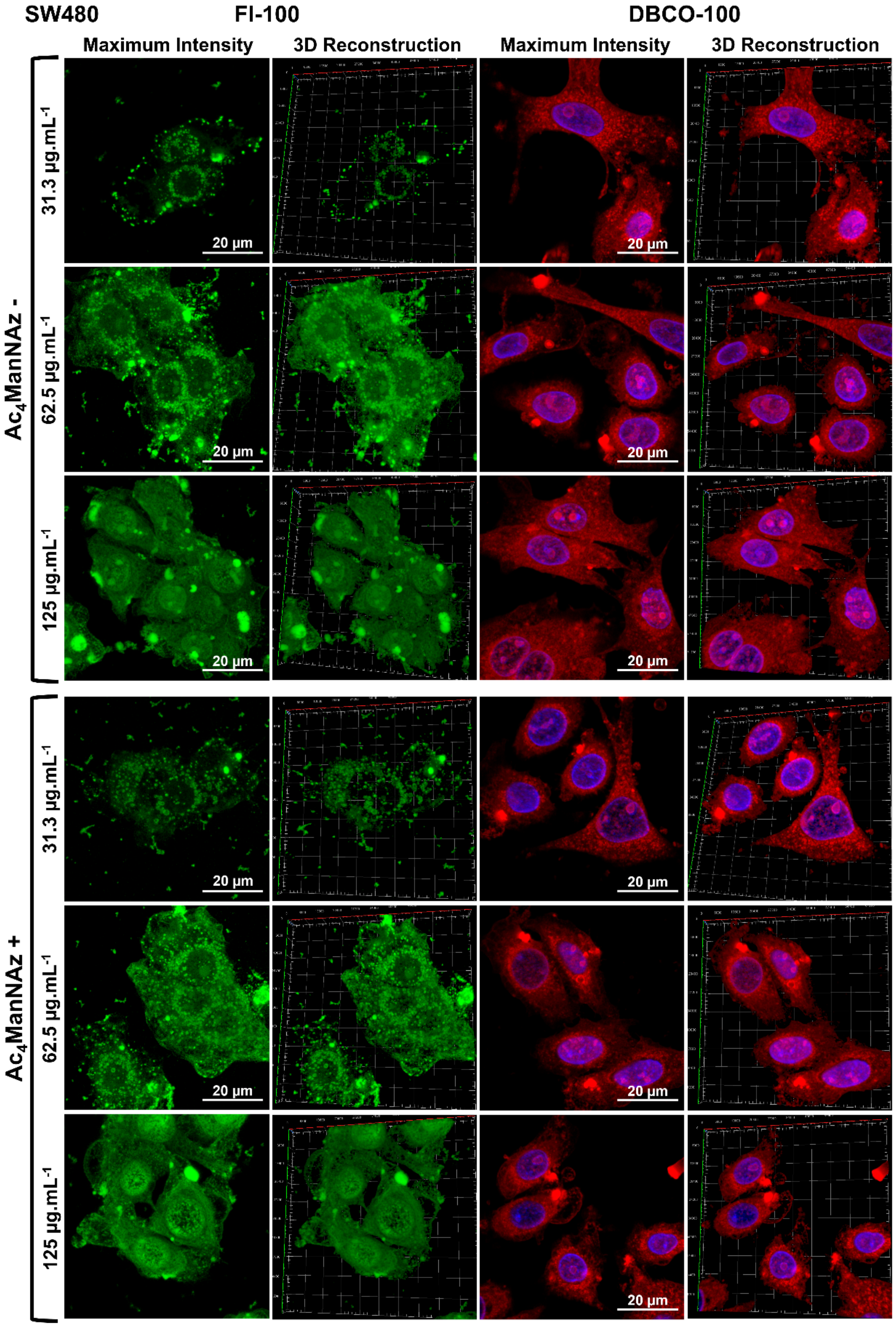

Figure S42. *Summary maximum intensity projection and 3D reconstructed confocal images of SW480 cells treated with (+) and without (-) Ac$_4$ManNAz (40 µM, 96 h) and treated with DBCO-pDMAEMA$_{100}$-Fl (**FL-100**, 31.3 – 125 µg.ml^{-1}, 2.5 h, Green). Images were also taken of cells treated with DBCO-pDMAEMA$_{100}$ (**DBCO-100**, 31.3 – 125 µg.ml^{-1}, 2.5 h) and stained with CellMask^{TM} membrane stain (red) and NucBlue^{TM} stain (blue). Scale bar = 20 µm.*

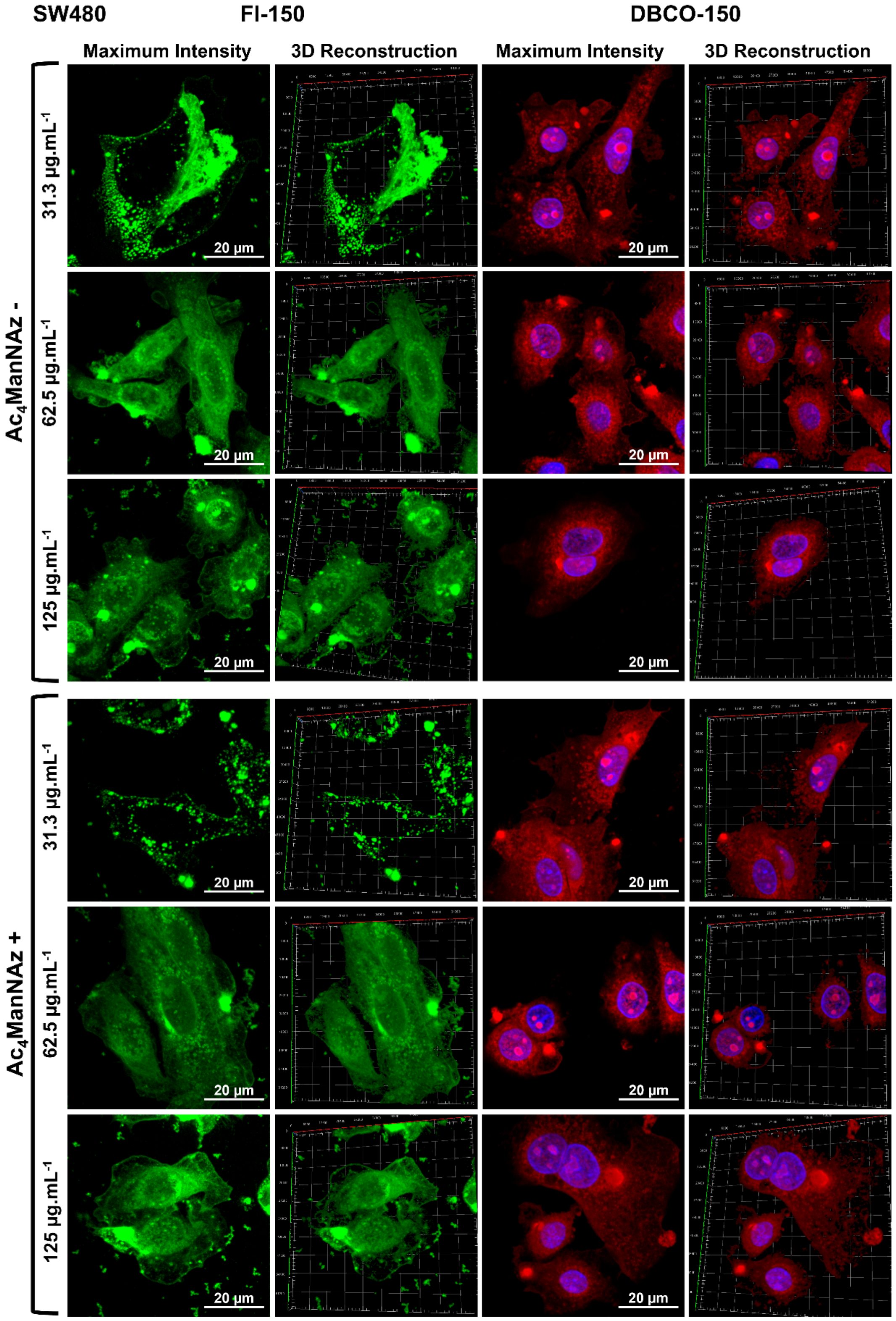

Figure S43. *Summary maximum intensity projection and 3D reconstructed confocal images of SW480 cells treated with (+) and without (-) $Ac_4ManNAz$ (40 µM, 96 h) and treated with DBCO-pDMAEMA$_{150}$-Fl (**FL-150**, 31.3 – 125 µg.ml^{-1}, 2.5 h, Green). Images were also taken of cells treated with DBCO-pDMAEMA$_{150}$ (**DBCO-150**, 31.3 – 125 µg.ml^{-1}, 2.5 h) and stained with CellMask™ membrane stain (red) and NucBlue™ stain (blue). Scale bar = 20 µm.*

2.10 Spheroid Proof Azido Labelling

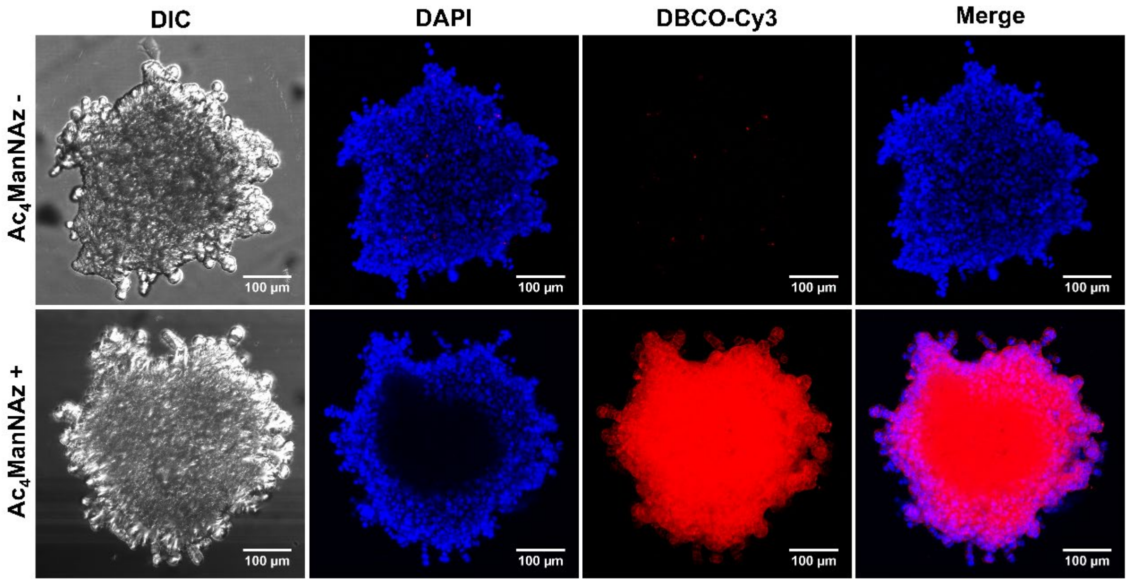

Figure S44. *Maximum intensity projection confocal images of A549 spheroids formed in the presence (+) and absence (-) of* $Ac_4ManNAz$ *(40 µM, 96 h) and treated with DBCO-Cy3 (50 µM, 2 h, red) and NucBlue™ nuclear stain (blue). Scale bar = 100 µm.*

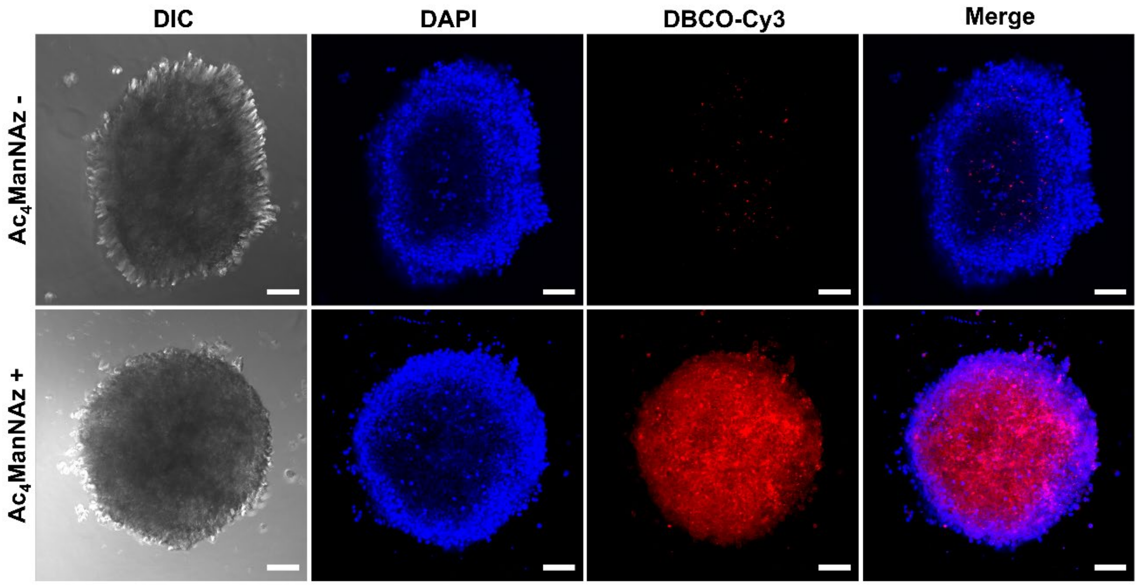

Figure S45. *Maximum intensity projection confocal images of pre-formed A549 spheroids (72 h) and subsequently treated with (+) or without (-)* $Ac_4ManNAz$ *(40 µM, 96 h) and treated with DBCO-Cy3 (50 µM, 2 h, red) and NucBlue™ nuclear stain (blue). Scale bar = 100 µm.*

2.12 Spheroid Cytotoxicity Assays

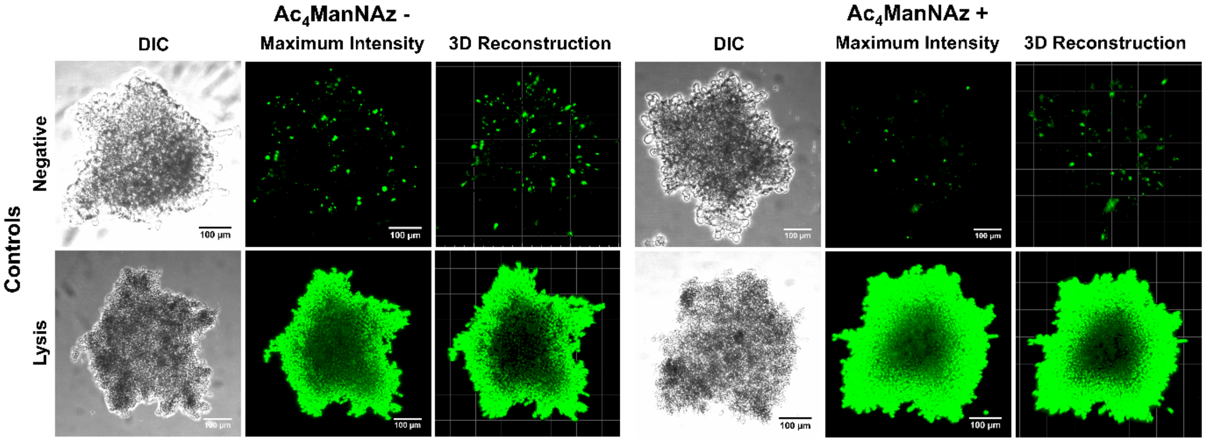

Figure S46. *Ac₄ManNAz spheroid toxicity controls. Spheroids treated with (+) and without (-) Ac₄ManNAz (40 µM, 96 h) were incubated with media (negative control) or lysis buffer (positive control) for 2.5 h and subsequently stained with CellTox™ green. Scale bar = 100 µm.*

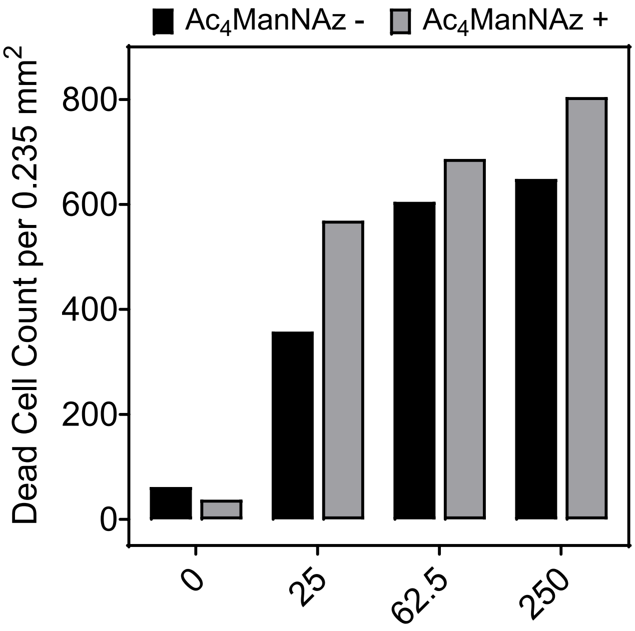

Figure S47. *Spheroids treated with (+) and without (-) Ac₄ManNAz (40 µM, 96 h) and treated with DBCO-pDMAEMA₁₀₀ (0 – 250 µg.ml^{-1}, 3 h) were stained with CellTox™ green to test for toxicity, confocal imaged (Figure S44) and the number of dead cells per average spheroid area (0.235 mm^2) was calculated and plotted.*

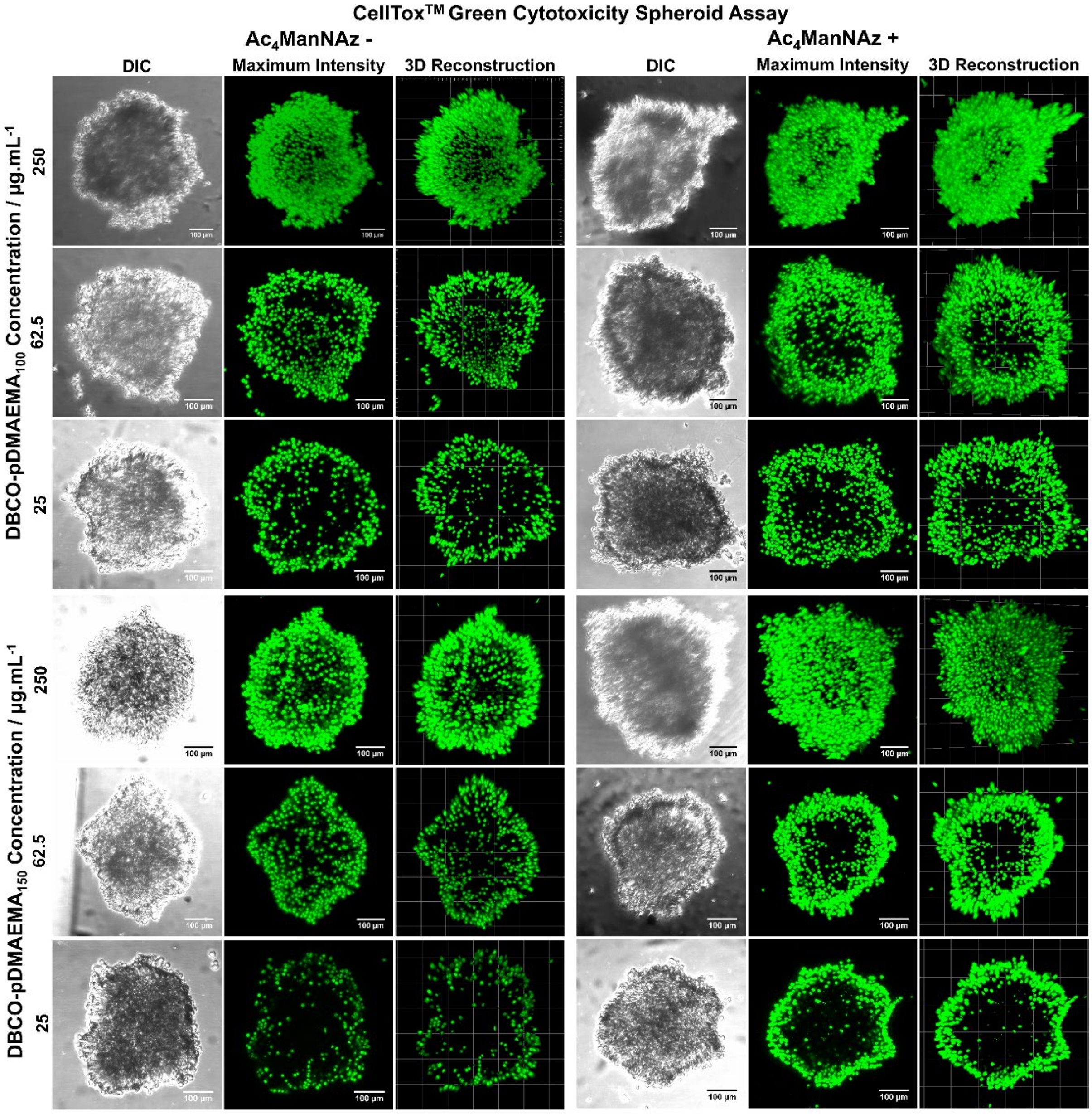

Figure S48. *Spheroids treated with (+) and without (-) Ac4ManNAz (40 µM, 96 h) and treated with DBCO-pDMAEMA100/150 (25 – 250 µg.ml^{-1}, 3 h) were stained with CellTox^{TM} green to test for toxicity. Scale bar = 100 µm.*

3.0 References

1. Richards, S. J., Otten, L. & Gibson, M. I. Glycosylated gold nanoparticle libraries for label-free multiplexed lectin biosensing. *J. Mater. Chem. B* **4**, 3046–3053 (2016).

2. Tomás, R. M. F., Martyn, B., Bailey, T. L. & Gibson, M. I. Engineering Cell Surfaces by Covalent Grafting of Synthetic Polymers to Metabolically-Labeled Glycans. *ACS Macro Lett.* **7**, 1289–1294 (2018).

3. Tomás, R. M. F. & Gibson, M. I. Optimization and Stability of Cell-Polymer Hybrids Obtained by 'clicking' Synthetic Polymers to Metabolically Labeled Cell Surface Glycans. *Biomacromolecules* **20**, 2726–2736 (2019).
